# Mining triggers extensive additional deforestation in sub-Saharan Africa

**DOI:** 10.1038/s41586-026-10551-2

**Published:** 2026-06-03

**Authors:** Oscar Morton, Christopher G. Bousfield, Prince Dégny Valé, Ieuan Lamb, Victor Maus, Robert G. Bryant, David P. Edwards

**Affiliations:** 1 Ecology and Evolutionary Biology, School of Biosciences, The University of Sheffield, Sheffield, UK; 2 Université Jean Lorougnon Guédé, Daloa, Côte d’Ivoire; 3 Centre Suisse de Recherches Scientifiques en Côte d’Ivoire, Abidjan, Côte d’Ivoire; 4 Institute for Ecological Economics, Vienna University of Economics and Business, Vienna, Austria; 5 Advancing Systems Analysis Program, International Institute for Applied Systems Analysis, Laxenburg, Austria; 6 School of Geography and Planning, The University of Sheffield, Sheffield, UK; 7 Department of Plant Sciences, University of Cambridge, Cambridge, UK; 8 Centre for Global Wood Security, University of Cambridge, Cambridge, UK; 9 Conservation Research Institute, University of Cambridge, Cambridge, UK

**Keywords:** Environmental impact, Conservation biology, Tropical ecology

## Abstract

Demand for minerals sourced from sub-Saharan Africa is expanding rapidly^1–5^. If poorly managed, mining expansion poses a key threat to tropical forests across the continent^6,7^. Here we present a spatiotemporal assessment of mining-driven deforestation of dense forests across Africa, using continent-wide data on post-deforestation land uses and a robust difference-in-differences framework to assess 16,627 mines between 2001 and 2020. In total, we find 187,000 hectares of direct mining-driven deforestation, that is, deforestation due to features directly associated with mining operations, such as pits, tailing ponds and spoil heaps. We estimate that mining also triggers an additional 8.0 percentage points (pp; 95% confidence interval (CI): 7.2–8.9 pp) increase in deforestation within 1 km of a mine compared with unmined areas. Increased levels of deforestation (1.1 pp, 95% CI: 0.7–1.5) persist up to 20 km from mines even after ten years. For every hectare of direct deforestation due to the mine footprint, mining triggers, on average, 34 hectares of additional offsite loss within five years through ancillary activities, including agriculture and settlements. Mines extracting cobalt and copper—key energy transition minerals—caused the highest amount of additional deforestation. Embedding offsite deforestation levels into environmental impact assessments for new mining projects will be key to ensuring zero-deforestation or no-net-loss supply chains for critical minerals and reduce future mining-driven forest losses in sub-Saharan Africa.

## Abstract (web summary)

Extensive mining activity leads to large-scale deforestation of dense tropical forests across sub-Saharan Africa, driven particularly by key energy transition minerals such as cobalt.

## Main

Global demand for metal ores and minerals is rising markedly^1,2^. The extraction of metal ores has quadrupled^3^ since 1970, fuelling rapid economic growth. The total, or embodied, volume of material stock used in buildings, infrastructure and machinery increased^4^ 23-fold between 1990 and 2010. Moreover, the conversion of currency into gold during global periods of financial upheaval has increased gold exploitation^5^, and the global shift towards renewable energy has further heightened demand for key energy transition minerals (ETMs), including cobalt, lithium and copper^8^.

Globally, mining directly threatens 8% of vertebrate species and drives widespread habitat loss^9,10^. This is concerning given that more than 20% of the remaining intact tropical forest lies in mineral, gas or oil concessions, many of which are still in the exploration stage^11^. Mine expansion affects forests directly when land is cleared for mining pits or shafts, on-site processing, spoil heaps, or tailing storage facilities. More than 325,000 hectares (ha) of tropical forest was lost directly to industrial mining^6^ between 2000 and 2019. However, focusing solely on mine footprints ignores ancillary offsite anthropogenic activities triggered by mining indirectly, including the construction of ports, roads and railways, settlements and agricultural expansion^12–14^. Each of these activities drives further deforestation^15,16^ and increases forest accessibility, facilitating hunting and trapping^17,18^. National-scale attempts to estimate total offsite deforestation caused by mining vary substantially from no-detectable-net impact in Zambia and Madagascar^19,20^, to significant net-negative impacts in the Democratic Republic of the Congo (DRC) and Brazil^21,22^. Such variability emphasizes the need for continental-scale assessments of the spatial and temporal impact of mining on direct and offsite (indirect) deforestation.

Here we uniquely quantify direct and offsite mining-induced deforestation of dense forest across sub-Saharan Africa by combining 16,627 distinct clusters of mines mapped in forested areas^23^ with 20-years of forest-cover data^24^ and by using recent advances in robust differences-in-differences (DID) inferential approaches^25,26^. We account, at the continental scale, for both direct mining deforestation driven by the mines themselves (such as pits and tailing ponds), and offsite deforestation through other processes (including road construction and agricultural and urban expansion) that may be triggered by mine establishment. Sub-Saharan Africa has the largest known global reserves of many strategically important minerals^7^, including 48% of cobalt and manganese reserves^27^, and has seen surges in foreign investment fuelling a mineral boom and rapid mining expansion^28^. Its forests contain globally vital carbon stocks^29^ and are hotspots of biodiversity^30^; thus, it is imperative that mining expansion be sustainable and well managed across the continent. Specifically, we address four key questions assessing (1) what the spatiotemporal footprint of direct mining-driven deforestation across sub-Saharan Africa is; (2) how much total additional deforestation (summed direct and offsite) is driven by mine establishment; (3) how much of the total additional deforestation triggered by mining occurs offsite through ancillary activities relative to the direct mine footprint; and (4) how the total additional deforestation varies between mined commodities.

## Deforestation footprint of African mines

**Fig. 1:**
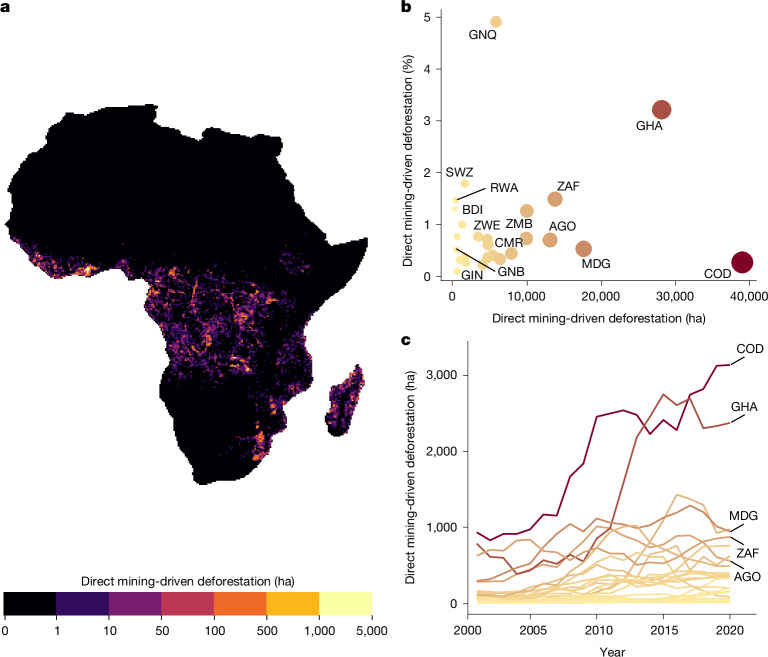
Direct mining-induced deforestation across sub-Saharan Africa between 2001 and 2020. **a**, Area (ha) of direct mining-induced deforestation of dense forest per 30 km^2^ cell. **b**, Direct mining-induced deforestation compared with the percentage of national deforestation accounted for by direct mining-induced deforestation between 2001 and 2020. **c**, Three-year average annual direct mining-induced deforestation per country between 2001 and 2020. Each line represents a single country. The five largest countries in terms of summed direct mining-induced deforestation during the analysed period are labelled. Only countries with more than 100 ha of mining-induced deforestation are shown. A full list of three-letter country codes and the corresponding country name is provided in Supplementary Table 2.

Across sub-Saharan Africa, there was 187,070 ha of direct mining-induced deforestation of dense forest (≥50% tree cover) between 2001 and 2020 (Fig. 1a) in 16,627 distinct clusters of mines in forested areas (>33% forest cover within 0–5 km, where forest is defined as ≥50% tree cover) identified using a pan-African dataset of post-deforestation land uses^23^. One-third (*n* = 6,028) of mines were located in the DRC, where 39,000 ha of direct mining-driven deforestation occurred. The DRC, Ghana (28,000 ha) and Madagascar (17,500 ha) together accounted for 45% of all direct mining-induced deforestation of dense forest across sub-Saharan Africa (Fig. 1b and see Supplementary Table 1 for national values). When considering the proportion of all national deforestation between 2001 and 2020 that was caused by mining (Fig. 1b), the highest losses were in Equatorial Guinea (4.9%), Ghana (3.2%) and Eswatini (1.5%). Of the 35 sub-Saharan African countries where direct mining-induced deforestation was detected frequently (that is, in ten or more years), 25 demonstrated increasing trends (Mann–Kendall test, *P* < 0.05) in annual direct mining-driven deforestation (Fig. 1c). These included four of the five most heavily mined countries (the DRC, Ghana, Madagascar and Angola, but not South Africa).

## New mines drive additional deforestation

**Fig. 2:**
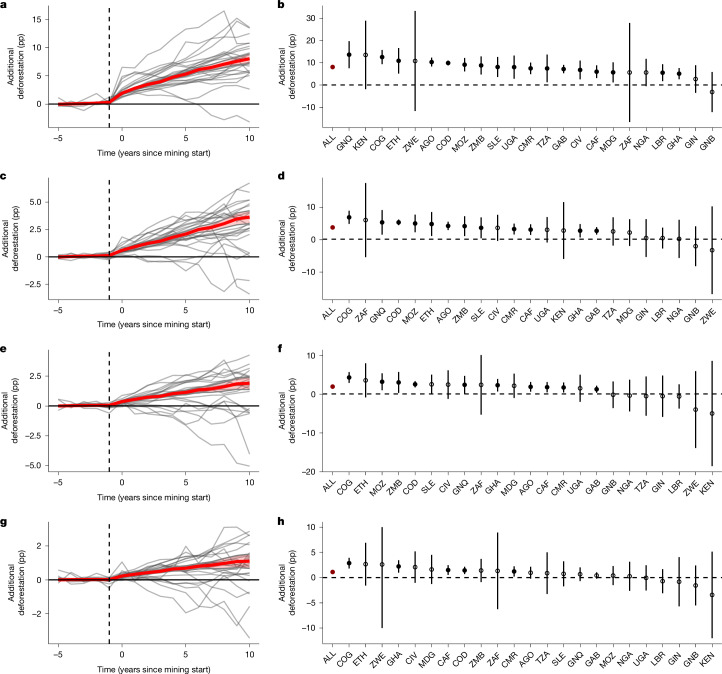
Estimated total additional (direct and offsite) deforestation after mining is detected, across time and space in sub-Saharan Africa. **a**,**c**,**e**,**g**, Additional deforestation within 0–1 km (**a**), 1–5 km (**c**), 5–10 km (**e**) and 10–20 km (**g**) buffers. Individual country mean average treatment effect on the treated (ATT) estimates derived from our DID framework are shown in grey; mean estimates and 95% CIs across sub-Saharan Africa are shown in red and pale red, respectively (*n* = 15,477 clusters). **b**,**d**,**f**,**h**, Summary of estimated additional pp of deforestation after ten years for all included countries (black) and across sub-Saharan Africa (red) within 0–1 km (**b**), 1–5 km (**d**), 5–10 km (**f**), and 10–20 km (**h**). Note, the *y* axis in **h** is cropped to ease visualization, the uncertainty for Zimbabwe (ZWE) extends beyond what is shown. Closed circles denote a statistically significant effect; open circles denote effects that are not significant. Points are mean ATTs and error bars are 95% CIs. Supplementary Fig. 2 includes a covariate-conditioned version of this analysis.

The cumulative additional total deforestation (summed direct and offsite) triggered by mining was estimated using heterogeneity-robust DID methods^25^ to track deforestation in concentric ring buffers around mines before and after mining operations started, using a ‘not yet treated’ quasi-experimental design (Methods). Across sub-Saharan Africa, mining caused an overall mean increase in deforestation of 8.0 pp (95% CI: 7.2–8.9 pp) within 1 km of a mine after ten years compared to unmined areas (Fig. 2a). At 1–5 km from a mine, the region-wide average effect was smaller, but remained substantial, with at a 3.6 pp (95% CI: 3.0–4.2 pp) increase in cumulative deforestation after ten years (Fig. 2c). Between 5–10 km and 10–20 km from mines, cumulative deforestation across ten years increased by 1.9 pp (95% CI: 1.4–2.4 pp; Fig. 2e) and 1.1 pp (95% CI: 0.7–1.5 pp; Fig. 2g), respectively. The greatest impacts in each concentric buffer predominantly occurred in the years immediately after the mine was established (Fig. 2a,c,e,g), and cumulative impacts plateaued after five years in some countries. Thus, mining had the greatest effect on total additional deforestation near mining sites and in the years immediately after establishment. Furthermore, total additional deforestation was correlated with mine size: the largest mines had the greatest additional forest losses (Supplementary Fig. 1).

The amount of additional deforestation within 1 km of a mine ten years after operations started increased in 18 of the 23 countries (Fig. 2b), with increases in deforestation ranging from 5.1 pp (95% CI: 2.5–7.6 pp) in Ghana to 13.6 pp (95% CI: 7.5–19.7 pp) in Equatorial Guinea. At 1–5 km from a mine, increased deforestation persists for 12 of the 23 countries (Fig. 2d), ranging from 2.6 pp (95% CI: 1.4–3.7 pp) in Gabon to 6.7 pp (95% CI: 4.7–8.8 pp) in the Republic of the Congo. Almost half (10 out of 23 countries; Fig. 2f) had a statistically detectable impact at 5–10 km from mining epicentres, varying from 1.2 pp (95% CI: 0.3–2.1 pp) in Gabon to 4.3 pp (95% CI: 2.9–5.7 pp) in the Republic of the Congo. Ghana, Cameroon, the Central African Republic, the Republic of the Congo and the DRC all had statistically significant impacts up to 10–20 km from mining sites (Fig. 2h).

We repeated our main analyses using a recently proposed alternative heterogeneity-robust two-stage imputation-based DID estimator and a weighted stacked DID estimator^31^. The results from these reanalyses support our findings and, in some cases, suggest that the estimates may be conservative (Supplementary Note 1 and Supplementary Figs. 2–8). We also repeated our analyses using both approaches after conditioning on a suite of covariates, the results of which were more conservative but remained comparable (Supplementary Note 1).

## Offsite losses far exceed mine footprint

**Fig. 3:**
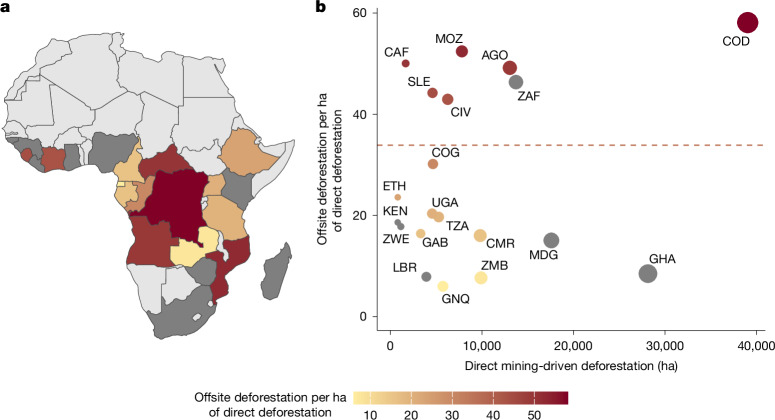
Relative impacts of direct mining-induced deforestation and offsite deforestation (such as agriculture and infrastructure) after mining is detected. **a**, Ratio of offsite to direct dense-forest loss in a 0–5 km buffer, five years after mining detection (that is, the number of ha of deforestation that occurs offsite per ha of direct deforestation). Countries in light grey were not included in the analysis (see Methods), countries in dark grey were included but did not have significant estimates of direct or offsite additional deforestation estimates after five years. **b**, Scatter plot of the total dense-forest area lost directly because of mining and the ratio of mean additional offsite to direct deforestation five years after mining detection. Countries and points are coloured according to this ratio; the point size is relative to the total area deforested directly by mining (2001–2020; see *x* axis for values). Dark grey points are countries where either direct or offsite additional deforestation estimates had an uncertain direction but a positive mean effect; all yellow to red coloured points and countries had significant additional direct and offsite deforestation estimates. For three countries (Nigeria, Republic of Guinea and Guinea-Bissau), we were unable to estimate distinct direct or indirect effects; these are not plotted. The dashed line denotes the sub-Saharan African estimate.

Offsite deforestation triggered by mine establishment (such as road construction and agricultural and urban expansion) varied substantially between countries and was, in most cases, much larger after five years than the losses attributed to the direct mining footprint (Fig. 3a and Supplementary Figs. 9–11). Across sub-Saharan Africa, we estimate that for each hectare (ha) of deforestation attributed directly to the mine footprint, an additional 33.9 ha of dense forest was lost to offsite drivers. Most offsite losses were due to the additional agricultural expansion triggered by mine establishment. Concurrent settlement expansion was an important driver of loss too, with losses from road development representing only a small portion (Supplementary Figs. 9 and 10). The greatest relative offsite impacts were in the DRC, with 58.1 additional ha of total offsite deforestation (Fig. 3). The scale of direct forest loss in the DRC (39,000 ha) means that mining-driven deforestation levels in this country are of severe concern for conservation efforts (Fig. 3b). Large relative offsite impacts were also detected in Mozambique (52.4 ha of offsite loss per direct ha), the Central African Republic (50.0 ha), Angola (49.2 ha), Sierra Leone (44.2 ha) and Côte D’Ivoire (42.9 ha), although the direct mining footprint in these nations is each less than one-third of the loss seen in the DRC. Other nations, such as Equatorial Guinea, had a much lower additional footprint (6.0 ha) coupled with the direct mine footprint having a low total area (Fig. 3b). These estimates agree strongly with the reanalyses performed using an alternative heterogeneity-robust two-stage DID estimator (Supplementary Fig. 4 and Supplementary Note 1).

## High impact of critical mineral mining

**Fig. 4:**
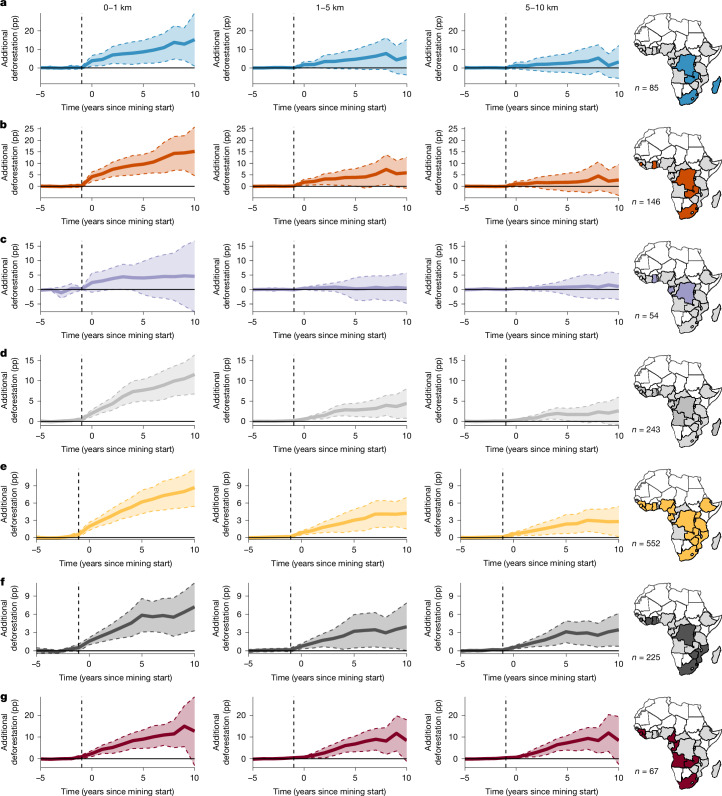
Estimated additional deforestation per key commodity across space and time in sub-Saharan Africa. **a**–**g**, Additional percentage points of deforestation detected in concentric buffer rings of 0–1 km, 1–5 km and 5–10 km concentric buffer rings around mines extracting cobalt (**a**), copper (**b**), manganese (**c**), diamonds (**d**), gold (**e**), silver (**f**) and iron (**g**). Solid lines denote the mean additional deforestation; and the dashed ribbon indicates the 95% CI. Maps show countries with each commodity per row in colour; all included countries are shown in grey. The number of mines for each commodity is indicated. Estimates for titanium and uranium are shown in Supplementary Fig. 13. The *y-*axis scale varies between panels but remains constant within a panel. Commodities are obtained by combining the mine data in this study with a global dataset on mined commodities^32^.

Of the 16,627 mines detected, 1,127 (around 7%) could be linked to at least one specific mineral being extracted using registered mining databases^32^. Additional total deforestation triggered by mining varied substantially depending on the mineral mined (the commodity; Fig. 4). Mines at which cobalt (15.27 pp; 95% CI: 0.9–29.6 pp) and copper (15.16 pp; 95% CI: 4.6–25.7 pp) were extracted were associated with the highest additional cumulative deforestation within 1 km of a mine after ten years (Fig. 4a,b), with reduced impacts remaining sporadically up to 1–5 km from mines; however, no significant impacts were seen in the 5–10 km and 10–20 km buffers (Supplementary Fig. 12). Manganese mines were the only mineral mines to have no statistical evidence of additional cumulative deforestation in any buffer ring or time period (except for a small directional effect after three years within 1 km; Fig. 4f), although the sample size was limited (*n* = 54).

Mines at which high-value minerals were extracted all showed similar responses, triggering additional cumulative deforestation within 1 km after ten years of 11.6 pp (95% CI: 6.8–16.4 pp) for diamond, 8.7 pp (95% CI: 5.5–11.9 pp) for gold and 7.2 pp (95% CI: 3.3–11.2 pp) for silver (Fig. 4c,d,g). Significant cumulative effects persisted for up to 5 km for diamond, silver and gold mines and for up to 10 km after ten years for silver mines. Iron mines had the largest area of effect, with significant additional cumulative deforestation occurring in all concentric ring buffers up to nine years after establishment (Fig. 4e). In the 0–1 km buffer, we estimate 14.5 pp (95% CI: 5.6–24.4 pp) of additional deforestation from iron ore mines. This decreases to an additional 11.9 pp (95% CI: 3.4–20.3 pp) in the 5–10 km buffer and 9.7 pp (95% CI: 0.96–18.6) in the 10–20 km buffer (Supplementary Fig. 12). Despite the small number of known mapped mining sites (*n* = 26), uranium mines triggered a significant 16.4 pp (95% CI: 4.5–28.3 pp) increase in cumulative deforestation within 1 km after ten years, but changes in cumulative deforestation at in the 1–5 and 5–10 km buffer zones were mostly uncertain (Supplementary Fig. 13).

Our estimates of additional mining-induced deforestation are based on the best available data, but these data still have caveats. First, although the post-deforestation land-use dataset used is the most comprehensive available for the region, its starting period of 2001 may mean that some of the mines first detected in 2001–2002 were actually established before 2000. However, a sensitivity analysis in which mines identified in the first two years were removed produced highly comparable results to the main analysis (Supplementary Fig. 14). Second, the mining-commodity data used are the most comprehensive available but have key omissions; data are missing particularly for poorly documented regions, unregulated or small-scale mining sites and construction minerals^33^. This restricts our analysis of commodity impacts to a subset of mines that have commodity data available; thus, we encourage caution when generalizing these results. Futhermore, we focus on dense forest (>50% tree cover), which excludes a subset of mining-driven deforestation in regions with drier, more open forest with lower tree cover (for example, areas of southern and eastern Africa). We also do not examine any potential increases in degradation, particularly from small-scale or sub-canopy mining, because such increases are difficult to detect at 30 m resolution^34,35^ and, as such, the study may underestimate the total extent and impact of small-scale mining.

## Discussion

This study provides comprehensive estimates of mining-induced deforestation of dense forest across sub-Saharan Africa, identifying 187,070 ha of direct mining-induced deforestation—for instance, from pits, tailing ponds and spoil heaps—between 2001 and 2020. This is almost four times the direct mining-induced deforestation footprint recorded across Africa in previous studies that were limited to industrial mining^6^. Crucially, this direct deforestation is dwarfed by an 8 pp increase in total deforestation triggered within 1 km of mined areas compared with unmined areas. Moreover, increased deforestation effects persist up to 20 km from mines. This represents a 34-fold increase in additional deforestation relative to on-site loss, through ancillary activities such as agriculture, urban expansion and road construction. Effectively managing and minimizing additional mining-associated deforestation is therefore critical to maintaining the globally vital carbon stocks and biodiversity of sub-Saharan Africa^7,29,30^.

Previous studies that quantify mining-driven deforestation at the regional scale have predominantly focused on quantifying the losses driven by the direct mining footprint^6^. Finer-scale studies focusing on offsite mining impacts have found additional deforestation up to 5 km from 255 artisanal mines in the eastern DRC^22^, up to 10 km from 446 mine clusters in the southern Côte d’Ivoire^36^ and up to 70 km from 50 large industrial mines in the Brazilian Amazon^21^. The substantially larger area of impact in the Brazilian Amazon, relative to this study and prior estimates across the African continent, is likely a product of the larger mine size and the creation of considerable extra road and rail infrastructure to link large mines and ports^21^.

Economic development and the global energy transition will continue to drive a boom in mining activities across Africa, with demand for key ETMs sourced predominantly from Africa expected to grow by up to 40-fold by 2040^8^. Although this represents an important opportunity for socio-economic development in many African countries that rely on artisanal and industrial mining as a key element of their economy^37,38^, our results underscore the severe risks to the continent’s remaining forest habitats under business-as-usual mining practices. Future environmental impact projections for cobalt mining—the DRC alone accounted for 80% of global cobalt production^39^ in 2024—suggest that these impacts will only continue to grow and their reduction is linked tightly to the success of mineral recycling initiatives^40^. Furthermore, the extensive offsite deforestation impacts of mining (Fig. 3 and Supplementary Fig. 4) are considered rarely but must form a key element of environmental impact assessments and licensing procedures for new mining projects^6^. This is particularly important for proposed sites that are close to protected areas or lands of Indigenous populations, given that most ETM projects in Africa are located on or near land belonging to Indigenous peoples or small-holder farmers and pastoralists^41^.

Offsite deforestation risk is further compounded by the diversity of mechanistic pathways for impacts around mines (such as settled workers and agricultural expansion) that require improved understanding. Although we examine the expansion of land uses around mines, how differing mine features may contribute to heterogeneity remains an important research frontier. One important distinction is between industrial and artisanal mining, because these two forms will drive differing amounts of additional deforestation through differing population movements, food requirements and associated infrastructure^22,42^. We show that larger mines have a greater deforestation footprint, but mine size is ineffective at distinguishing between industrial and artisanal mining (for example, very large-scale artisanal mining sites in Kolwezi and Mutanda in the DRC). Further work is required to map the different forms of mining and evaluate their individual offsite deforestation impacts. Mitigating offsite impacts may also be hampered especially in areas with weak governance, lax enforcement or corruption^43,44^. Furthermore, some of the mines included in this study, and many operating across Africa more broadly^45^, are unregistered, informal or illegal and therefore not subject to environmental impact assessments.

Advances in satellite imagery and machine-learning methods^23^ facilitated our analysis of mining-induced deforestation across sub-Saharan Africa. However, at a global scale, comprehensive wall-to-wall data on the spatial distribution of mines are still lacking, and are limited mostly to information about mines reported in online databases^33^. We therefore lack a regional-scale understanding of the additional mining deforestation footprint (both direct and offsite) across specific commodities in other key biodiversity and carbon hotspots that are particularly threatened by mining activity, such as much of South America and the tropical lowland forests of Southeast Asia^3,29,30^. Our understanding of where specific minerals are extracted is limited, and our study is only able to link fewer than 10% of mines to a commodity. This data paucity hampers our ability to distinguish between and address commodity-specific environmental impacts at scale and highlights the need to work with the mining community to develop a better understanding of mining footprints and environmental outcomes^46^. This issue goes beyond the impact of mining on deforestation, as shown here, to include other environmental effects, such as groundwater and air pollution^47^, human health impacts^48^ and degradation of both forest and non-forest natural ecosystems^49^. Quantifying these mining impacts is a key future research direction. An important issue will also be the impact of mining sites being abandoned, whether because continued operations at the mine are not economically feasible or because deposits are exhausted. Evidence suggests that, in addition to mine establishment^22^, mine abandonment also drives a spike in deforestation, too, because communities seek to establish alternative livelihoods^3,50^.

Given the accelerating global demand for minerals, it is essential to embed robust environmental impact assessments at the point of supply, with transparent supply-chain traceability until the point of demand, to assess and mitigate the environmental and societal harms from poorly regulated mining enterprises^51^. Enhanced monitoring and mitigation verification of global mining activities will only be possible in places in which advances in satellite imagery and modelling methods can produce comprehensive maps of mining activities and track onsite and offsite leakage and environmental degradation. This is a key prerequisite for moving towards a system-level understanding, which links the sources of mineral demand to spatially explicit impacts at specific mining sites. This is the only way to shift towards zero-deforestation or no-net-loss supply chains, as already done in the agriculture and food sector^52,53^. Progressing towards this goal is essential to ensure that the ongoing and future green energy transition is not built on a foundation of avoidable deforestation.

## Methods

### Data

To map and analyse the spatial extent of direct mining-induced deforestation of dense forest across sub-Saharan Africa, we used previously published data^23^ that map post-deforestation land use across sub-Saharan Africa between 2001 and 2020 at a resolution of 30 m. The dataset first used the global forest change data^24^ to identify areas of forest loss between 2001 and 2020, before combining an active learning framework with high-resolution (5 m) Planet–Norway’s International Climate and Forests Initiative data to train a deep-learning model that predicts post-deforestation land use. Post-deforestation land use is assigned to one of 15 different classes by the model, one of which is mining. Mining is defined as land used for extractive subsurface and surface mining activities (such as underground and strip mines, quarries and gravel pits), including all associated surface infrastructure as described previously^23^. Mining as a post-deforestation land use is mapped with high accuracy, with a 98% user’s accuracy and an 82% producer’s accuracy (see ref. ^23^ for original accuracy metrics). We used all instances of mining mapped previously^23^ to represent areas of mining activity in this analysis.

The mining data are presented at a resolution of 30 m pixels, with pixels representing either direct mining-induced deforestation or not. It was thus important to group proximate mining pixels together to create distinct ‘clusters’ of mining activity for use in the analysis. We therefore used distance-based density clustering to group together all nearby mining pixels into one cohesive mining cluster. Clustering was performed to group together all pixels within 1 km of another mining pixel, with a minimum of 5 pixels required to form a cluster. Notably, this clustering method does not require any predefined shape or size of clusters, allowing clusters to be created that can accurately reflect the staggered growth of mining activities, which can often spread across long distances and follow particular directions (for example, the growth of mining activities along a riverbank). After performing the clustering process, 67,586 distinct mining clusters remained across sub-Saharan Africa. However, because we were interested in mining-induced deforestation, we then filtered these mining clusters to retain only clusters that were located in densely forested regions, which we defined as having more than one-third dense forest cover (defined as pixels with ≥50% tree cover) in a 5 km buffer from the mine cluster at the start of the analysis period in 2000. We did not consider areas to be forest if they were classified as plantations by the latest version (v.2) of the Spatial Database of Planted Trees (SDPT)^54^. This final filtering step left 16,627 mining clusters in forested areas for analysis.

### Deforestation measures around mines

We define three different forms of deforestation associated with mining activity in and around our mining clusters.

First, direct deforestation defined as annual deforestation caused directly by the mine in the mining cluster footprint (such as, pits and tailing ponds). This includes all pixels with ≥50% tree cover in 2000 that became deforested between 2001 and 2020 with the end use classified as mining ^23^.

Second, offsite deforestation defined as annual deforestation through any other processes (such as, road construction, and agricultural and/or urban expansion), outside the mining footprint that may be triggered by mine establishment. This represented all pixels that with ≥50% tree cover in 2000 that became deforested between 2001 and 2020 as described previously^24^ (v1.11) and that were not classified as mining^23^ and were not identified as plantations in the SDPT v.2 data^54^.

Third, total deforestation defined as the annual sum of both the direct and offsite deforestation.

### DID framework

**Extended Data Fig. 1:**
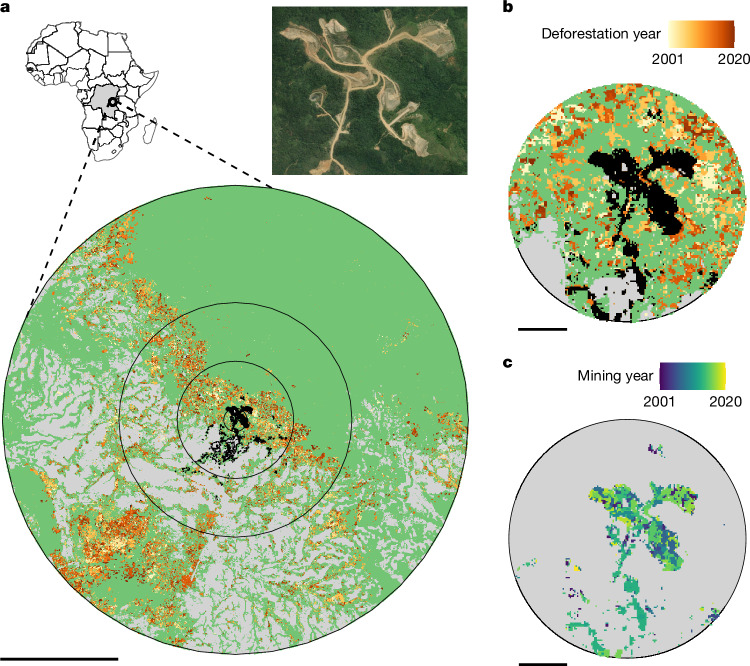
Schematic of our quasi-experimental design. **a**, Location of an example gold mine (central black pixels) in the DRC. Green denotes the forest cover in 2000; yellow to red shading denotes deforestation (of any form) between 2001 and 2020; and grey denotes non-forested areas. Black concentric circles highlight the 1 km, 5 km, 10 km and 20 km buffer rings. Inset, satellite image of the mine in November 2020. The photograph is from ESRI, Vantor, Earthstar Geographics and the GIS User Community. **b**, Magnification of the mine cluster and the year of deforestation of the surrounding forest cover for the example mine. **c**, The year of forest loss for areas of forest that underwent direct mining-driven deforestation between 2001 and 2020. Scale bars, 10 km (**a**) and 1 km (**b** and **c**).

To estimate the additional total deforestation triggered by mine establishment, we used recent advances in heterogeneity-robust DID models. To assess mining-induced deforestation across spatial scales, we created four concentric ring buffers of increasing size (0–1 km, 1–5 km, 5–10 km and 10–20 km) around the centre of each mine (Extended Data Fig. 1). We defined our response variable as the total (sum of offsite and direct) deforestation around clusters in each buffer per year between 2001 and 2020. We calculated the total annual deforestation in each buffer as a proportion of the total forested area (≥50% tree cover) present in 2000.

We leveraged the staggered nature with which mining operations commenced in a DID quasi-experimental design that incorporated not-yet-treated mining sites as controls. Mining clusters are classed as treated from the year 10% of pixels in that cluster are deforested owing to mining in the mining cluster. The preceding not-yet-treated period corresponds to the period before mining commences in a cluster. See the section ‘Sensitivity analyses’ for analyses of alternative cut-offs for defining the start of mining operations. Mining clusters that were always treated (those with mines present in the first year) or only treated in the last year could not be included. Therefore, 15,477 clusters could be used in total for estimation. An alternative paradigm to using not-yet-treated mines would be to use statistical matching to balance covariates that drive variation in either the outcome or assignment between mining clusters and comparable never-treated sites. However, it is possible that even post-matching, never-treated sites may differ systematically from treated sites in both treatment assignment and outcomes in a manner not likely captured by matching variables (such as the presence of appropriate minerals and the type of sediment or rock).

We used a recently proposed group–time average treatment effect DID estimator^25^ that is robust to heterogeneous treatment effects and staggered study designs. This estimator identifies the group–time-specific average treatment effect on the treated (ATT(*g*, *t*) as defined in equation 1), where the group is the year mining clusters are first treated (*g*, mining first detected) and observed in calendar year (*t*). Thus, for mining first detected in year *g* and observed in the year *t*, the estimate is the difference in *Y* (cumulative deforestation as a proportion of forest cover in 2000) years *g* – 1 and *t* across mines that commence in year *g*, minus the same difference for mining clusters in which mining is detected in later years but not in year *t* (termed the not-yet-treated clusters and defined by *G* the time period a unit becomes treated and the binary indicator *D*_*t*_).

1 $${\rm{A}}{\rm{T}}{\rm{T}}(g,t)={\mathbb{E}}[{Y}_{t}-{Y}_{g-1}|G=g]-{\mathbb{E}}[{Y}_{t}-{Y}_{g-1}|{D}_{t}=0,G\ne g]$$

These group–time specific ATTs do not enforce homogenous treatment effects across all time periods or groups (first year of mining detection). Group–time ATTs were then aggregated into dynamic treatment effects relative to the year mining was detected. Standard errors were clustered at the mine cluster level.

We applied this approach at the national level for all countries in sub-Saharan Africa with 30 or more identified mining clusters. We included a total of 23 sub-Saharan countries with sufficient coverage; 6 countries with insufficient numbers of clusters were dropped from the national analyses (South Sudan, *n* = 4; Rwanda, *n* = 9; Malawi, *n* = 4; Comoros, *n* = 24; Burundi, *n* = 15; and Eswatini, *n *= 6). The number of clusters per country included in the models ranged from 32 (Guinea-Bissau) to 5,069 (the DRC). For each country, we applied the group–time-specific estimator on total deforestation in concentric buffer rings of 0–1 km, 1–5 km, 5–10 km and 10–20 km around each mining cluster to estimate the total additional deforestation attributable to the average mining cluster per country. We also estimated an effect across sub-Saharan Africa using all clusters from all countries with mining-driven forest loss detected (29 countries, 15,477 clusters). This estimate followed the same approach as laid out previously for country-level estimates, except we included country as an additional fixed effect. This was repeated for each of the four increasing concentric buffer rings.

We calculated pseudo-ATTs to assess the pre-treatment assumption of parallel trends. We did this for all national concentric ring buffers for a shift of 1–5 years. Across all buffers and time periods, there was strong evidence for parallel trends. Only 1–2 countries out of 23 showed evidence of non-parallel trends, and this was most common for the year before a mine was established in the 1 km buffer.

To assess the relative size of additional direct deforestation compared with additional offsite deforestation, we used the previously outlined DID framework to separately estimate the number of ha of additional direct and offsite deforestation triggered by mine establishment separately. We then post-processed these estimates to estimate the offsite deforestation for each ha of direct deforestation, defined as the additional offsite loss divided by the direct loss. We estimate this for a 0–5 km buffer around all mines at the national and sub-Saharan African scale. We only calculated ratios for countries where the effects of both the direct and offsite model were significant five years after mine establishment. In an additional analysis, we further disaggregated the non-mining deforestation data (indirect) to obtain annual time series specifically for deforestation driven by agriculture, settlements, and roads (as derived previously^23^) in the 0–5 km buffer (Supplementary Information). We then modelled this in the same dynamic DID framework as described above to elucidate the additional impact mine establishment specifically had on agricultural and settlement expansion and road development (Supplementary Figs. 9 and 10).

### Commodity varying impacts

We used a new database that includes 42,799 mine properties and 217,200 polygons, covering a total area of 145,738.1 km² globally^32^. This database links commodity data from^55^ the S&P Global Mine and Metals database^56^ and data from the Global Coal Mine Tracker of the Global Energy Monitor^57^, to previously published mining land-use polygons^33,58,59^. We overlaid this database with our 16,627 mining clusters and assigned commodities to clusters in cases in which the cluster was within 5 km of a mining site with a known commodity. Many mines extract more than one commodity or mineral and it is impossible to estimate at a regional or national scale the relative quantities of each extracted mineral or the relative influence of each mineral on mine expansion and deforestation. Therefore, each cluster was assigned all of the commodities known to be extracted at that site. We also repeated the analysis using only the main commodity listed per mine and these results aligned closely with those presented in the main text (Supplementary Fig. 15). In total, 1,127 clusters could be linked to known commodities. We then ran DID analyses as described previously for each commodity linked to at least 30 mining clusters. Owing to the relatively low sample size per commodity, we did this at the aggregated sub-Saharan African scale, but not at the national scale.

### Sensitivity analyses

We used a comprehensive suite of justifiable alternative approaches and alterations to our main approach that were decided a priori to commencing the main analyses to assess the robustness of our results to analytical choices. These are shown and discussed in detail in the Supplementary Information.

First, we repeated the national-level analysis and included covariates that may influence deforestation dynamics in the first stage of the DID estimator (Supplementary Fig. 2). The covariates included travel time from the nearest settlement with a population of more than 5,000 individuals^60^, population density^61^, elevation and slope^62^. The inclusion of covariates is often necessary to meet the parallel pre-trends assumption; however, we note our models without covariates already met this assumption (Supplementary Fig. 2).

Second, we repeated our whole analysis using an alternative, recently proposed two-stage imputation-based DID estimator^31^, which, similarly to our main text estimator, addresses biases that often hamper conventional estimators used to estimate DID (Supplementary Figs. 3–5). The first-stage model identifies cluster and year-specific fixed effects that would occur in the absence of any treatment from the not-yet-treated observations (such as the cluster- and time-specific effects on cumulative deforestation before the start of mining). Thus, the untreated outcome (cumulative deforestation), accounting for cluster and year fixed effects, can then be imputed and removed from the observed treated outcome. Additional covariates that are likely to affect trends in the cumulative outcome can also be incorporated in this first model (Supplementary Fig. 6). The second-stage model then regresses this residualized outcome on the time since mining operations started to estimate the dynamic ATT. Standard errors of the coefficients were clustered at the mine cluster level. For our data, this approach finds greater levels of deforestation than our main analysis group-time ATT approach and is more certain of these impacts in more countries. However, we note that, when checking the parallel trends assumption of this approach, we found that it failed this assumption for a number of countries and pre-treatment years, particularly in the 0–1 km and 1–5 km concentric buffers.

We also repeated our main analysis at the national and sub-Saharan African scale wide using a second alternative stacked DID trimmed aggregate ATT^63^. In brief, this modifies a standard stacked DID regression approach, which often fails to estimate a defined causal parameter because of improper weighting, to apply corrective weights when stacking to estimate a trimmed ATT focusing around a specific trimmed period before and after treatment. For our analysis, we created trimmed sub-experimental periods with a five-year pre-treatment and five-year post-treatment window. This method generates highly similar results to the approach described in the main text, but when checking the parallel trends assumption of this approach, we found that it performed inconsistently for our data (Supplementary Figs. 7 and 8).

Third, for the analysis described in the main text, we categorize the start of mining operations as when 10% of a mining cluster is deforested because of mining. This minimizes the risk that any single erroneously classified pixel substantially biases our analysis. However, we also reran our main analysis (Fig. 2) using two alternative criteria. The first was less conservative and assumes mining commences the year the very first pixel is lost from the cluster; and the second is more conservative and requires 20% of the cluster to be deforested by mining directly before mining operations are assumed to have commenced (Supplementary Figs. 16 and 17).

Fourth, although the gold standard for comprehensive analyses of mining impacts at scale is a highly accurate wall-to-wall geospatial map of mining sites that maximizes spatial coverage and minimizes omission errors, it is inevitable that, despite the high user accuracy of the data used here^23^, a small number of pixels may be misclassified as mining-driven. Thus, we also repeated our main analysis using an alternative manually verified dataset of mining sites^32,33^, which resulted in a smaller dataset of 2,504 mines in forested landscapes, according to our defined criteria (Supplementary Fig. 18).

Fifth, the spatial patterning and non-random clustering of mines around ore deposits and rivers results in many mines being established in the vicinity of existing mines. Thus, it can be difficult to differentiate between the direct impact of an individual mine and the spillover effects of other nearby mines. This may especially be the case in regions where mining activities have large areas of effect. To address this, we used a proposed modification of the two-stage imputation-based estimator described above to disentangle direct and spillover impacts^64^. This method modifies the first-stage imputation of the outcome in not-yet-treated mines to impute only the outcomes for mines that are both not-yet-treated and are also not exposed to potential spillover from nearby mines. Subsequently, the main treatment year (the year in which the mine became operational) and the spillover treatment year (the year the mines buffer first intersected with that of another mine) are then included in the second-stage regression to isolate both the direct effects of mining operations and the additional spillover effect probably attributable to nearby mines. We classified mines as being exposed to potential spillover effects if a mining cluster had another mining cluster within a 10 km radius. Thus, it is possible to separate the estimated effects directly due to the mining cluster from those of spillover effects from nearby clusters (Supplementary Figs. 19–22).

Finally, to assess whether our results are sensitive to the specific forest-loss data used, we repeated our analysis using the Tropical Moist Forest data from the European Commission Joint Research Centre^65^. We processed the dataset identically to the main analysis, generating mine-cluster-specific deforestation time series analogous to those used in the main analysis, because this dataset covers only a specific biome, unlike the data from ref. ^24^, this led to the analysis focusing on a subset of mines in areas dominated by tropical moist forest (*n* = 7,859). As a comparison, we also repeated the previously described analysis for this subset of mines to check for congruency or systematic differences due to the choice of forest-loss data (Supplementary Fig. 23).

### Reporting summary

Further information on research design is available in the Nature Portfolio Reporting Summary linked to this article.

## Online content

Any methods, additional references, Nature Portfolio reporting summaries, source data, extended data, supplementary information, acknowledgements, peer review information; details of author contributions and competing interests; and statements of data and code availability are available at 10.1038/s41586-026-10551-2.

## Supplementary information

Supplementary Information Supplementary Note 1, Supplementary Figs. 1–23 and Supplementary Tables 1 and 2.

Reporting Summary

Peer Review File

## Data Availability

All data used in this study are freely available for download online. The previously published post-deforestation land use dataset^23^ is available at Zenodo: 10.5281/zenodo.11065705; the tree-cover and forest-loss data are available at: https://storage.googleapis.com/earthenginepartners-hansen/GFC-2023-v1.11/download.html; and the plantation data from the SDPTv2 are available at: https://www.globalforestwatch.org/blog/data-and-tools/updated-planted-trees-map-near-global-coverage/. The processed data outputs needed to run the analyses are available at GitHub (https://github.com/OMorton/AFR_MiningForestLoss).

## Code availability

All code for the analysis is available at GitHub (https://github.com/OMorton/AFR_MiningForestLoss).
