# Supplementary material for: Mining triggers extensive additional deforestation in sub-Saharan Africa
Source: Nature. 2026 Jun 3;654(8120):971–7. doi: 10.1038/s41586-026-10551-2 (PMC13293856; doi:10.1038/s41586-026-10551-2)
Supplement: Supplementary file 1 — Supplementary Note 1, Supplementary Figs. 1–23 and Supplementary Tables 1 and 2. [file 41586_2026_10551_MOESM1_ESM.docx]

**Supplementary Information**

Oscar Morton*^,1+^, Christopher G. Bousfield*^,1+^, Prince Dégny Valé^2,3^, Ieuan Lamb^1^, Victor Maus^4,5^, Robert G. Bryant^6^ & David P. Edwards^7,8+^.

^1^ Ecology and Evolutionary Biology, School of Biosciences, the University of Sheffield, S10 2TN, UK.

^2^ Université Jean Lorougnon Guédé, Daloa, Côte d’Ivoire

^3^ Centre Suisse de Recherches Scientifiques en Côte d’Ivoire, Abidjan, Côte d’Ivoire

^4^ Institute for Ecological Economics, Vienna University of Economics and Business, Welthandelsplatz 1, Vienna, 1020, AT

^5^ Advancing Systems Analysis Program, International Institute for Applied Systems Analysis, Schlossplatz 1, Laxenburg, A-2361, AT

^6^ School of Geography and Planning, University of Sheffield, United Kingdom

^7^ Department of Plant Sciences and Centre for Global Wood Security, University of Cambridge, Cambridge, UK

^8^ Conservation Research Institute, University of Cambridge, Cambridge, UK

*Joint first authors

+Correspondence: [o.morton@sheffield.ac.uk](mailto:o.morton@sheffield.ac.uk), [c.bousfield@sheffield.ac.uk](mailto:c.bousfield@sheffield.ac.uk), [dpe29@cam.ac.uk](mailto:dpe29@cam.ac.uk).

**Supplementary text 1 – *Congruency across reanalyses***

To ensure our conclusions on the additional deforestation impacts driven by mine establishment across sub-Saharan Africa were robust, we also undertook a suite of alternative analyses probing the sensitivity of our main results to various changes in assumptions, methods, or reasonable alternate analytical decisions.

*(1) Covariate conditioning.*

While the DiD estimator used (*1*) passed the pre-trends assumption, we also assessed if conditioning on additional covariates likely to influence treatment assignment and pre-treatment deforestation trends altered our estimates. This analysis highlighted consistent sub-Saharan African wide estimates, and highly comparable national-level estimates at smaller buffer sizes. We note that as the buffer size increased beyond 5 km, conditioning on covariates led to increasingly conservative estimates and no statistical evidence of increased impacts post-mining for most countries (Supplementary Figure 2).

*(2) Alternate DiD estimators.*

We repeated the entire analysis (main text Figures 3-5) using an alternate 2-stage imputation-based DiD estimator (*2*). This yielded highly comparable, yet generally less conservative main effect estimates (Supplementary Figure 3). This was also true for the subsequent direct-offsite ratio of effects and commodity specific impacts (Supplementary Figures 4 and 5). Likewise, additionally conditioning on covariates in the first-stage model minimally affected overall estimates (Supplementary Figure 6). While these estimates are qualitatively similar to the group-time average treatment effects from using the Callaway and Sant’Anna estimator (*1*) we present in the main text (albeit less conservative) we caution against their direct use. Testing the pre-trends assumption of the 2-stage estimator by calculating pseudo-ATTs revealed many buffer and country combinations particularly at larger buffer sizes failed this key assumption, likely rendering the resulting 2-stage imputation-based ATTs biased and inaccurate.

Similarly, the stacked DiD estimator (*3*) yielded highly comparable estimates to our main text results. However, the parallel trends assumption was inconsistently met across buffers and country analyses and thus we encourage caution when interpreting solely these estimates (Supplementary Figures 7 and 8).

*(3) Alternate triggers for mining start year.*

*Altering* how we classify the commencement of mining had a minimal effect on our main results shifting our sub-Saharan Africa wide by a fraction of percentage point for any given time since mining with all estimated 95% confidence intervals remaining highly overlapping (Supplementary Figures 16 and 17). Broadly, comparing the single pixel, 10% and 20% deforested triggers for the commencement of mining, we note no clear directional shift in estimates becoming greater or lesser as the stringency of the trigger increases. We attribute this to the general high congruency between the years identified by the three triggers (years identified by the three triggers all have pairwise Pearson's correlation statistics >0.7), with many clusters having identical start years identified regardless of methods (e.g. mining begins and expands substantially in the same year).

*(4) Alternate mine location dataset.*

Comparing our results to a smaller but manually verified database of mining sites (*4*) highlighted near exact agreement between the ATTs derived from each source at all times since mining and across all buffers (Supplementary Figure 18). The 95% confidence intervals for our Masolele et al. (*5*) based mining clusters always fell within the intervals identified using the Maus et al. data. The greater uncertainty (although still significant increases in deforestation) derived from the Maus et al. data is likely a product of the much-reduced sample size of manually verified mines compared to the wall-to-wall GIS based inputs of the Masolele et al. data.

*(5) Assessing spatial-spillover.*

We additionally assessed the extent of spatial-spillover (or leakage), which would occur when the proximate location of mines leads to the effect of one mine contaminating the effect of other mines (*6*). This could potentially lead to sample bias by inflating pre-trends and or double counting post-mine detection impacts. We assessed the prevalence and extent of spatial spillover using an extension of the 2-stage estimator previously detailed (see Methods), which allows us to partition the impact of mining into spillover effects from nearby mines and direct impacts from the treated mine itself. From these data we note that the presence of spatial spillover varied considerably between countries (Supplementary Figures 19-22). There was little evidence of spillover in at least half of the 10-years after mining detection for 48% (11/23) of countries within 1 km of mining clusters, but in the largest 10-20 km concentric buffer spillover was detected in 61% (14/23) of countries in at least half of the 10-years after detection. This is largely an unavoidable feature of high-density mining areas.

*(6) Alternate forest loss data.*

Repeating our analysis using the JRC TMF forest loss data yielded highly similar results to using the Hansen et al., forest loss data when restricting this to mines from the tropical moist forest biome. We note very high congruency between mean estimates of forest loss across all buffers, and therefore consistent inference regardless of the forest loss data used (Supplementary Figure 23). However, there is greater uncertainty when using the JRC TMF data set particularly in the 10-20 km buffer zone. We suggest this is likely due to the JRC TMF data only capturing dense forest, typically 90% canopy cover, so the starting area is likely to be smaller and any subsequent losses more uncertain.


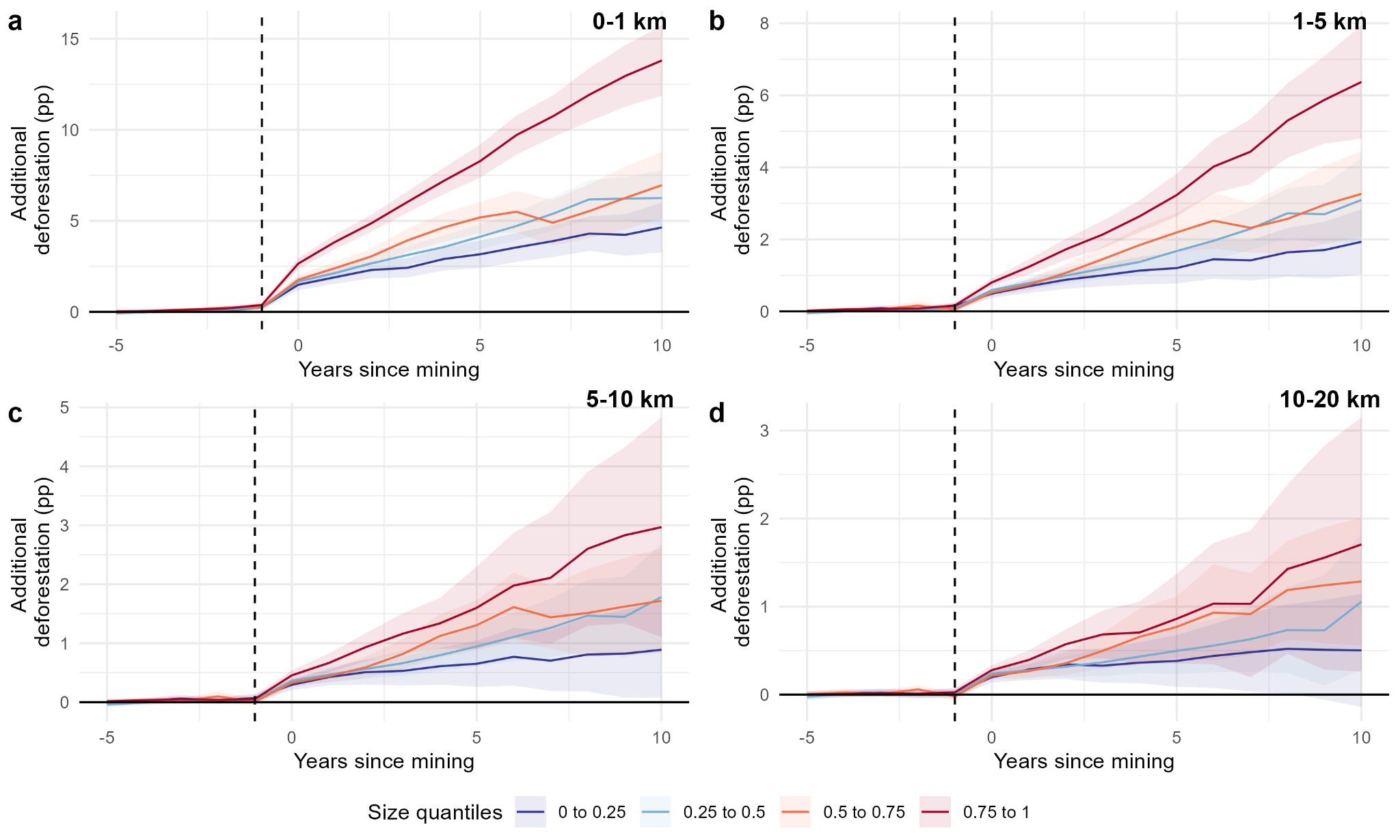


**Supplementary Figure 1. Estimated additional deforestation since mining is detected across mining size classes in Sub-Saharan Africa.** Additional percentage points (pp) of deforestation in the 0 - 1 km (a), 1 - 5 km (b), 5 -10 km (c), and 10 - 20 km (d) buffers. Colours denote mine size quantiles ranging from the bottom 25% (0 to 0.25) up to the largest quarter (0.75 – 1). Lines are mean ATTs and error bars are 95% confidence intervals.

**
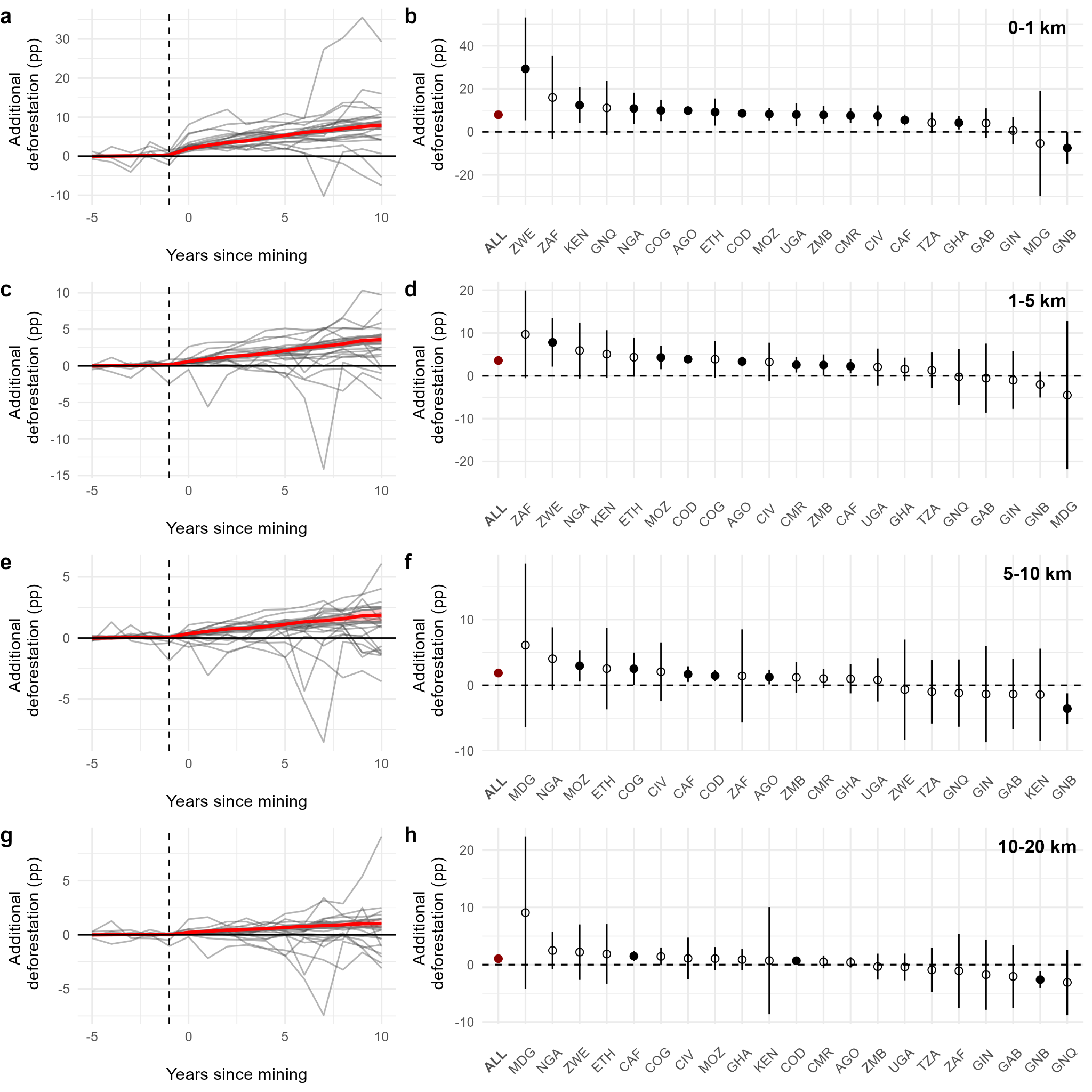
**

**Supplementary Figure 2. Estimated additional deforestation since mining is detected, across space and time in Sub-Saharan Africa after conditioning on covariates.** Additional percentage points (pp) of deforestation in the 0 - 1 km (a), 1 - 5 km (c), 5 -10 km (e), and 10 - 20 km (g) buffers. Individual country mean estimates are shown in grey and the sub-Saharan Africa wide mean estimates and 95% confidence interval (CI) are shown in red and pale red, respectively. Summary of estimated additional pp of deforestation after 10-years for all included countries (black) and the sub-Saharan Africa wide estimate (red) in the 0 - 1 km (a), 1 - 5 km (c), 5 -10 km (e), and 10 - 20 km (g) buffers. Closed circles denote a statistically significant effect; open circles denote statistically non-significant effects. Points are mean ATTs and error bars are 95% confidence intervals. See Supplementary Table 2 for a full list of 3 letter ISO codes and their corresponding country name in full. Note the results for SLE and LBR are not shown due to being unable to fit a model conditioned on the full suite of covariates.

**
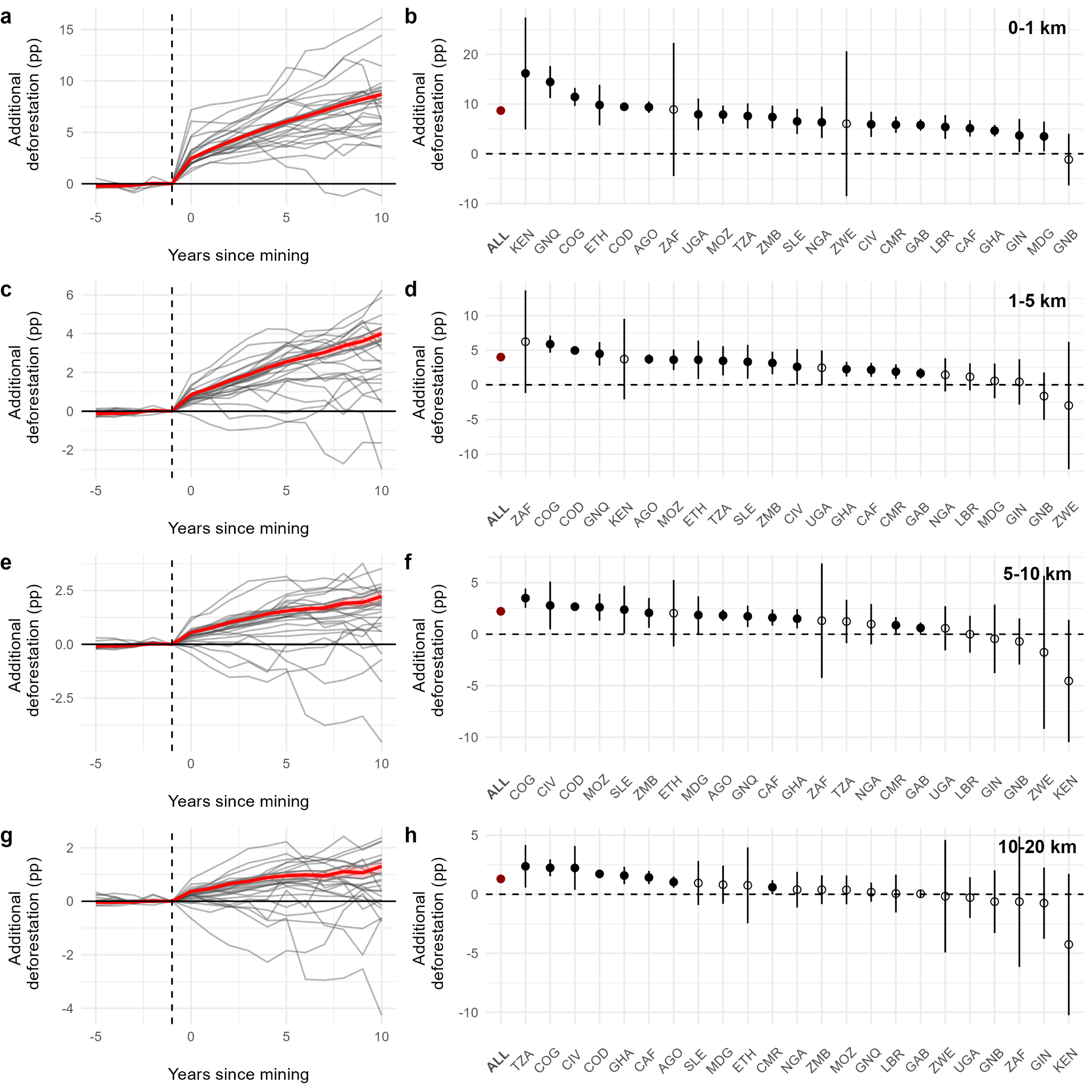
**

**Supplementary Figure 3.** **Estimated additional deforestation since mining is detected, across space and time in Sub-Saharan Africa using the alternate Gardner (2022) 2-stage estimator (repeat of main text Figure 2).** Additional percentage points (pp) of deforestation in the 0 - 1 km (a), 1 - 5 km (c), 5 -10 km (e), and 10 - 20 km (g) buffers. Individual country mean estimates are shown in grey and the sub-Saharan Africa wide mean estimates and 95% confidence interval (CI) are shown in red and pale red, respectively. Summary of estimated additional pp of deforestation after 10-years for all included countries (black) and the sub-Saharan Africa wide estimate (red) in the 0 - 1 km (a), 1 - 5 km (c), 5 -10 km (e), and 10 - 20 km (g) buffers. Closed circles denote a statistically significant effect; open circles denote statistically non-significant effects. Points are mean ATTs and error bars are 95% confidence intervals. See Supplementary Table 2 for a full list of 3 letter ISO codes and their corresponding country name in full.

**
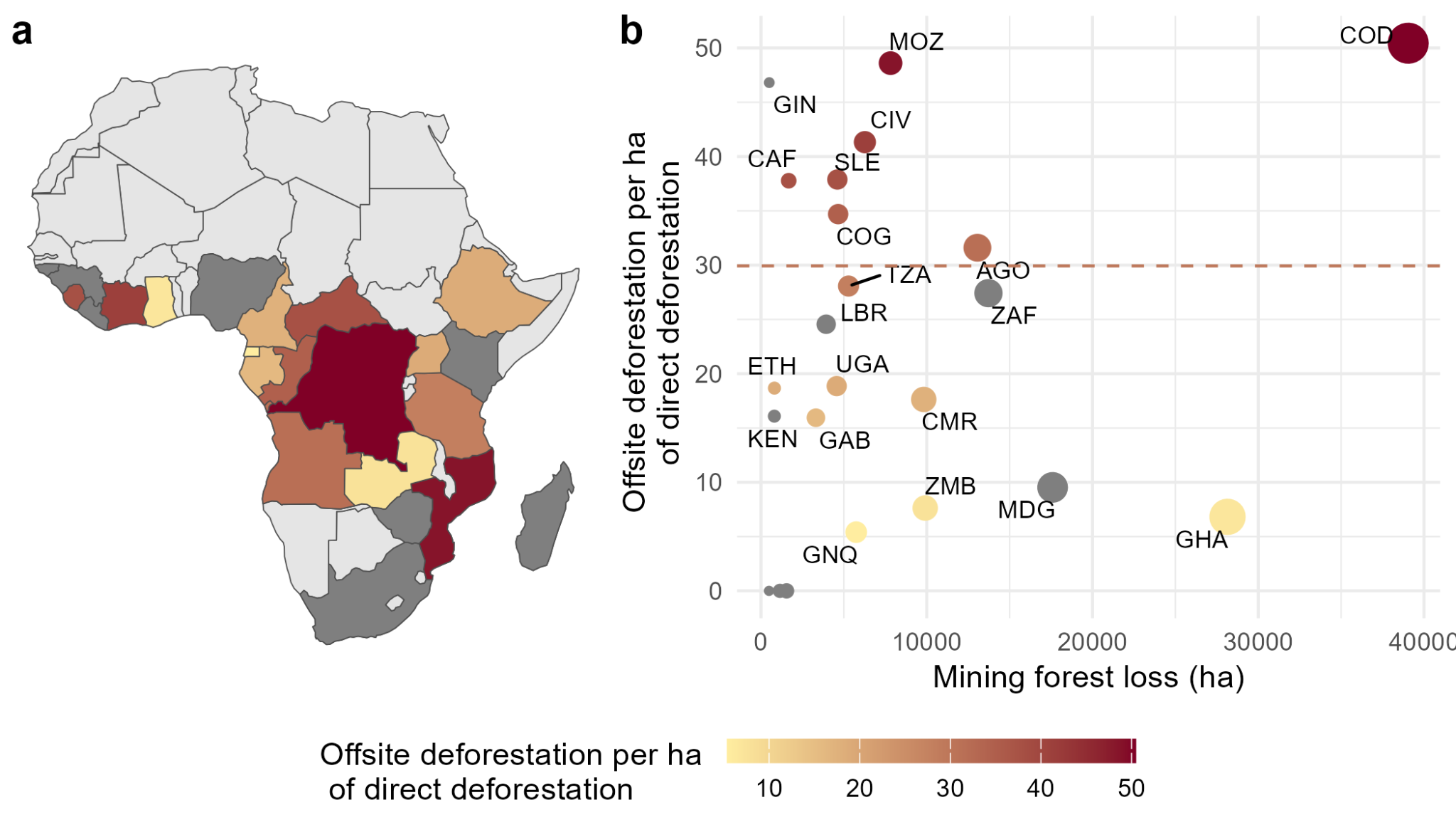
**

**Supplementary Figure 4. Relative impacts of direct deforestation attributed to mining and offsite deforestation (e.g. agriculture, infrastructure etc.) after mining detection using the alternate Gardner (2022) 2-stage estimator (repeat of main text Figure 3).** a - Ratio of offsite to direct forest loss (per 1 hectare of direct deforestation how many hectares of deforestation occur offsite). Countries in light grey were not included in the analysis (see Methods), countries in dark grey were included but had non-significant direct or offsite deforestation estimates after 5-years thus we do not calculate a ratio. b - scatter plot of the total forest area lost directly attributed to mining and the ratio of mean additional offsite to direct deforestation 5-years after mining detection. Countries and points are coloured by this ratio, and point size is relative to the total area deforested directly by mining (2001-2020).

**
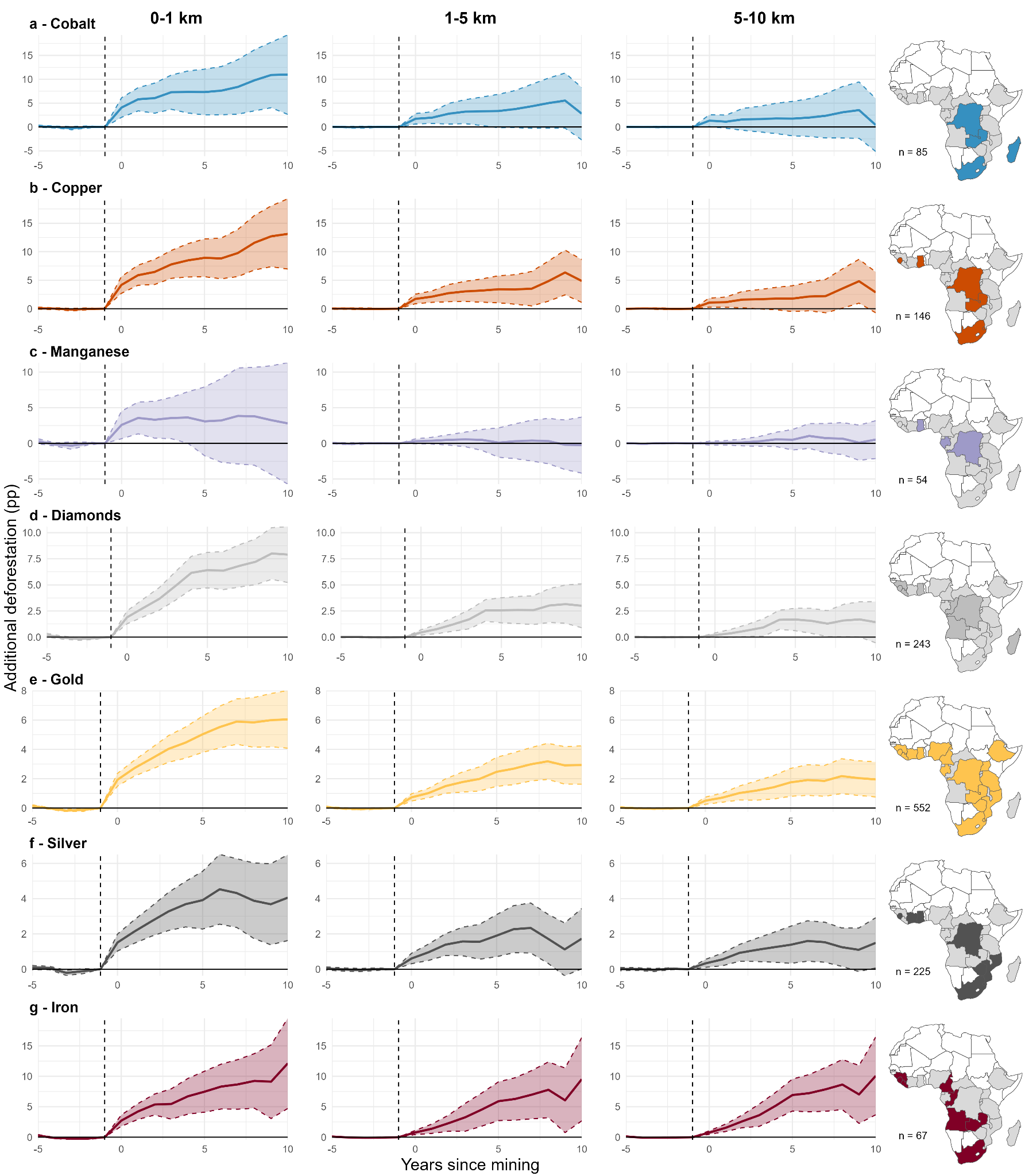
**

**Supplementary Figure 5. Estimated additional deforestation per key commodity across space and time in Sub-Saharan Africa using the alternate Gardner (2022) 2-stage estimator (repeat of main text Figure 4).** a - additional percentage points of deforestation after cobalt mining is detected in 0 - 1 km, 1 - 5 km and 5 -10 km concentric buffer rings. Solid lines denote the mean additional deforestation and the dashed ribbon the 95% confidence interval. Maps show countries containing the commodity per row in colour and all included countries in grey. Inset text denotes the sample size expressed as the number of distinct mining clusters per commodity. Subsequent rows show the same for mines extracting copper (b), manganese (c), diamonds (d), gold (e), silver (f), and iron (g). Note the y-axis scale varies between plots for clarity.

**
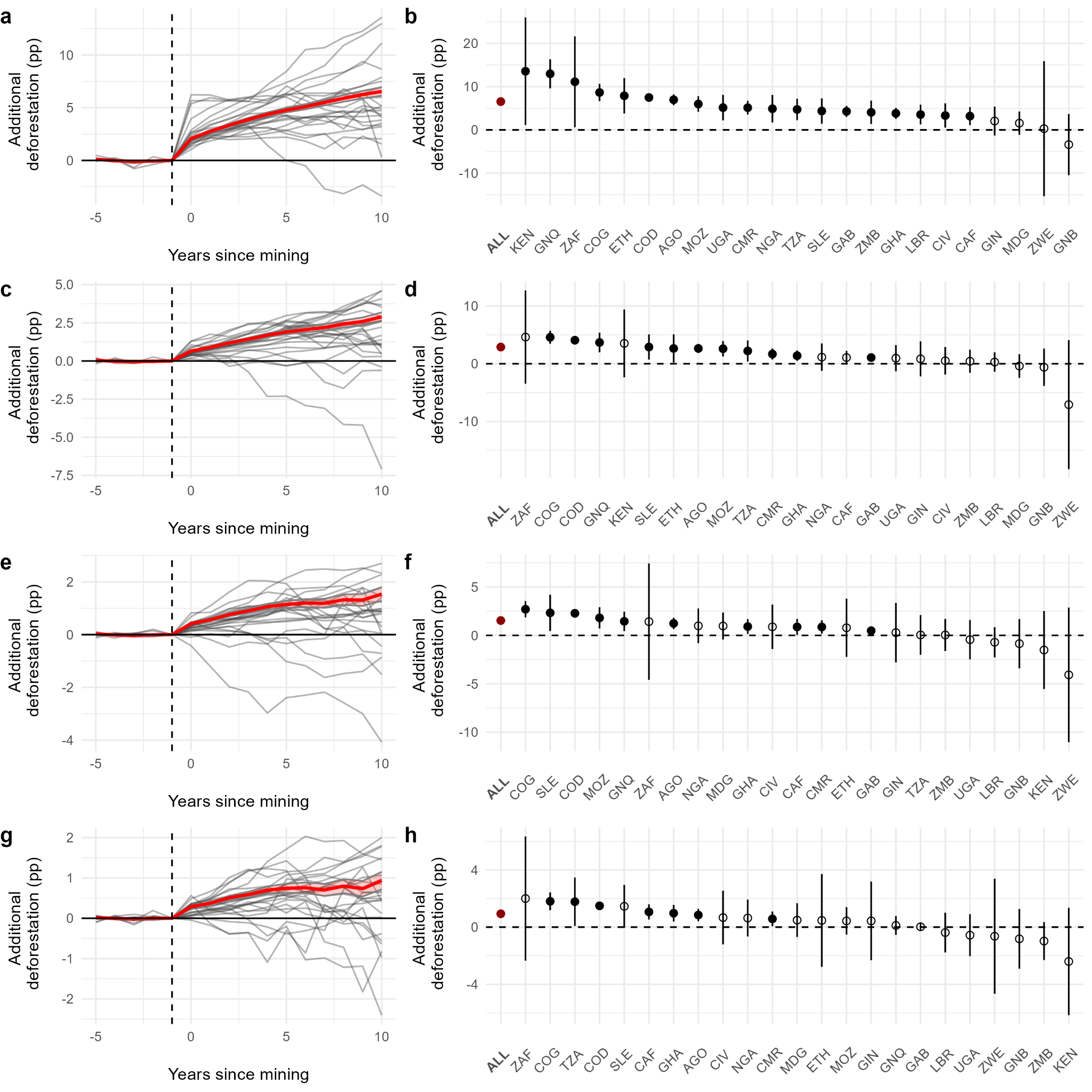
**

**Supplementary Figure 6. Estimated additional deforestation since mining is detected, across space and time in Sub-Saharan Africa after conditioning on covariates using the alternate Gardner (2022) 2-stage estimator (repeat of Supplementary Figure 2).** Additional percentage points (pp) of deforestation in the 0 - 1 km (a), 1 - 5 km (c), 5 -10 km (e), and 10 - 20 km (g) buffers. Individual country mean estimates are shown in grey and the sub-Saharan Africa wide mean estimates and 95% confidence interval (CI) are shown in red and pale red, respectively. Summary of estimated additional pp of deforestation after 10-years for all included countries (black) and the sub-Saharan Africa wide estimate (red) in the 0 - 1 km (a), 1 - 5 km (c), 5 -10 km (e), and 10 - 20 km (g) buffers. Closed circles denote a statistically significant effect; open circles denote statistically non-significant effects. Points are mean ATTs and error bars are 95% confidence intervals. See Supplementary Table 2 for a full list of 3 letter ISO codes and their corresponding country name in full.

**
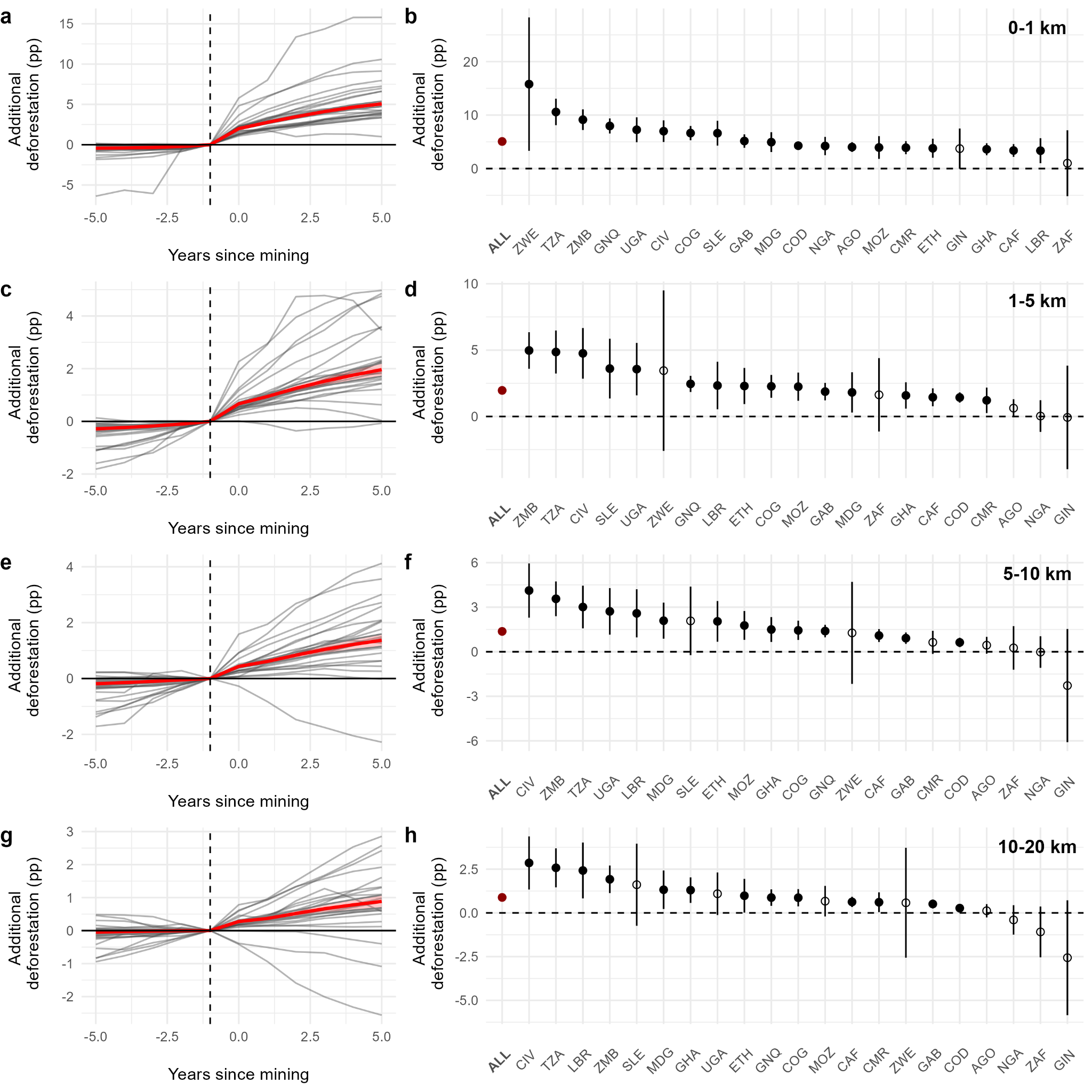
**

**Supplementary Figure 7.** **Estimated additional deforestation since mining is detected, across space and time in Sub-Saharan Africa using the alternate Wing et al (2024) stacked DiD approach (repeat of main text Figure 2).** Additional percentage points (pp) of deforestation in the 0 - 1 km (a), 1 - 5 km (c), 5 -10 km (e), and 10 - 20 km (g) buffers. Individual country mean estimates are shown in grey and the sub-Saharan Africa wide mean estimates and 95% confidence interval (CI) are shown in red and pale red, respectively. Summary of estimated additional pp of deforestation after 5-years for all included countries (black) and the sub-Saharan Africa wide estimate (red) in the 0 - 1 km (a), 1 - 5 km (c), 5 -10 km (e), and 10 - 20 km (g) buffers. Closed circles denote a statistically significant effect; open circles denote statistically non-significant effects. Points are mean ATTs and error bars are 95% confidence intervals. See Supplementary Table 2 for a full list of 3 letter ISO codes and their corresponding country name in full.


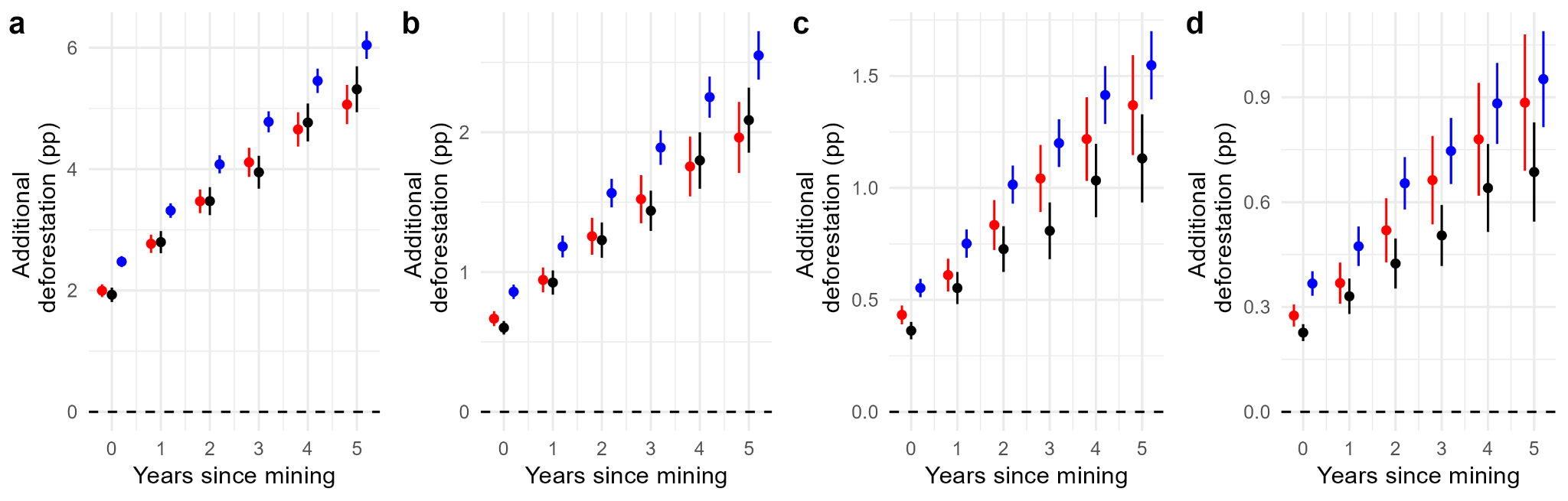


**Supplementary Figure 8. Comparative summary of additional deforestation estimates across three different DiD approaches.** Panels a - d respectively denote the 0-1 km, 1-5 km, 5-10 km and 10-20 km buffers. Points are mean ATTs and error bars are 95% confidence intervals. Points are coloured by method, Callaway and Sant’Anna (2021) - black, Gardner (2022) - blue and Wing et al., (2024) - red.


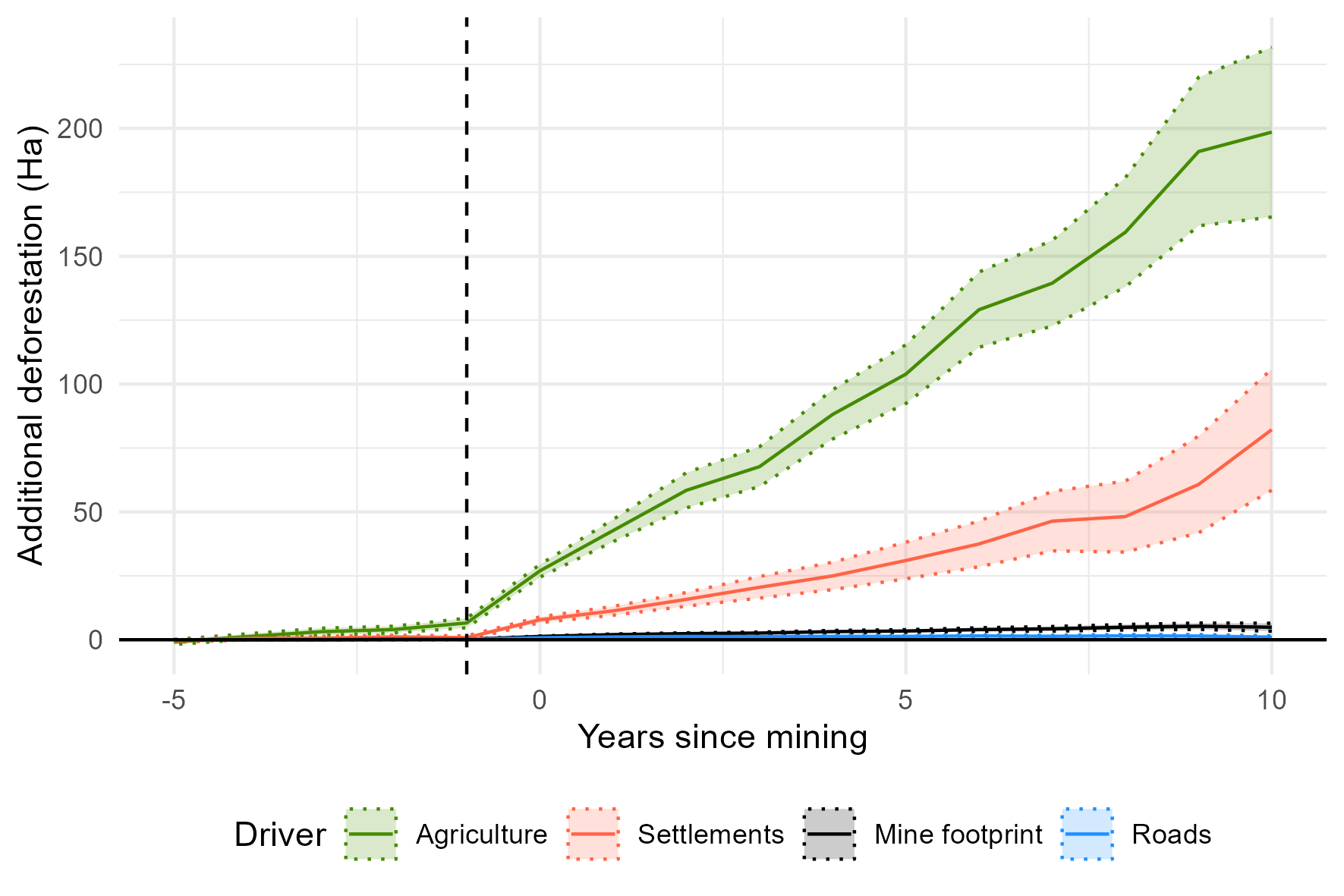


**Supplementary Figure 9. Additional cumulative deforestation in the 0-5 km mining buffer for four post-deforestation land uses across sub-Saharan Africa.** Lines are mean ATTs and error ribbons are 95% confidence intervals.


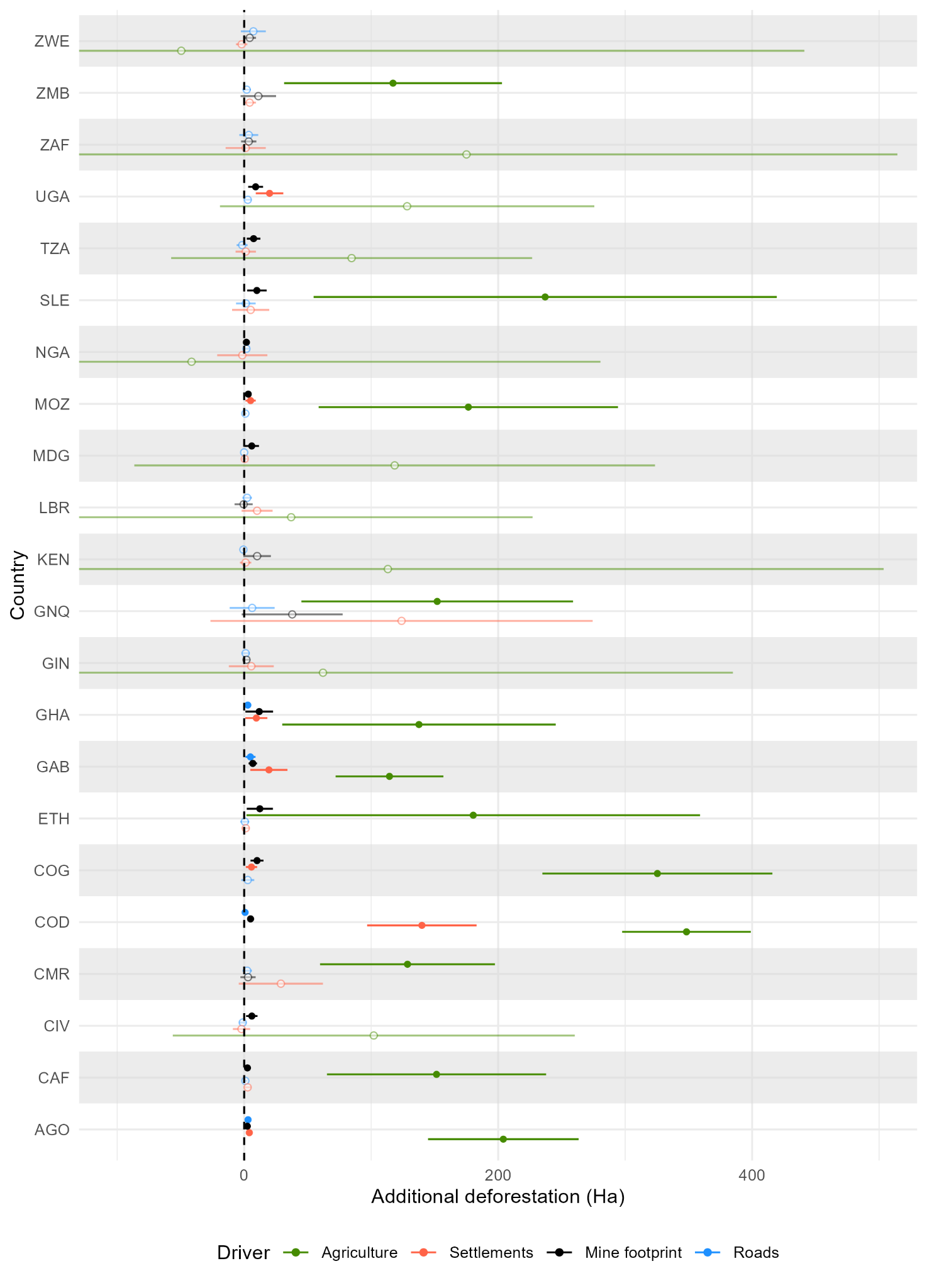


**Supplementary Figure 10. Additional cumulative deforestation in the 0-5 km mining buffer after 5-years for four post-deforestation land uses at the national level.** Points are mean ATTs and error bars are 95% confidence intervals. Closed circles denote a statistically significant effect; open circles denote statistically non-significant effects. The x-axis is manually trimmed for clarity.

**
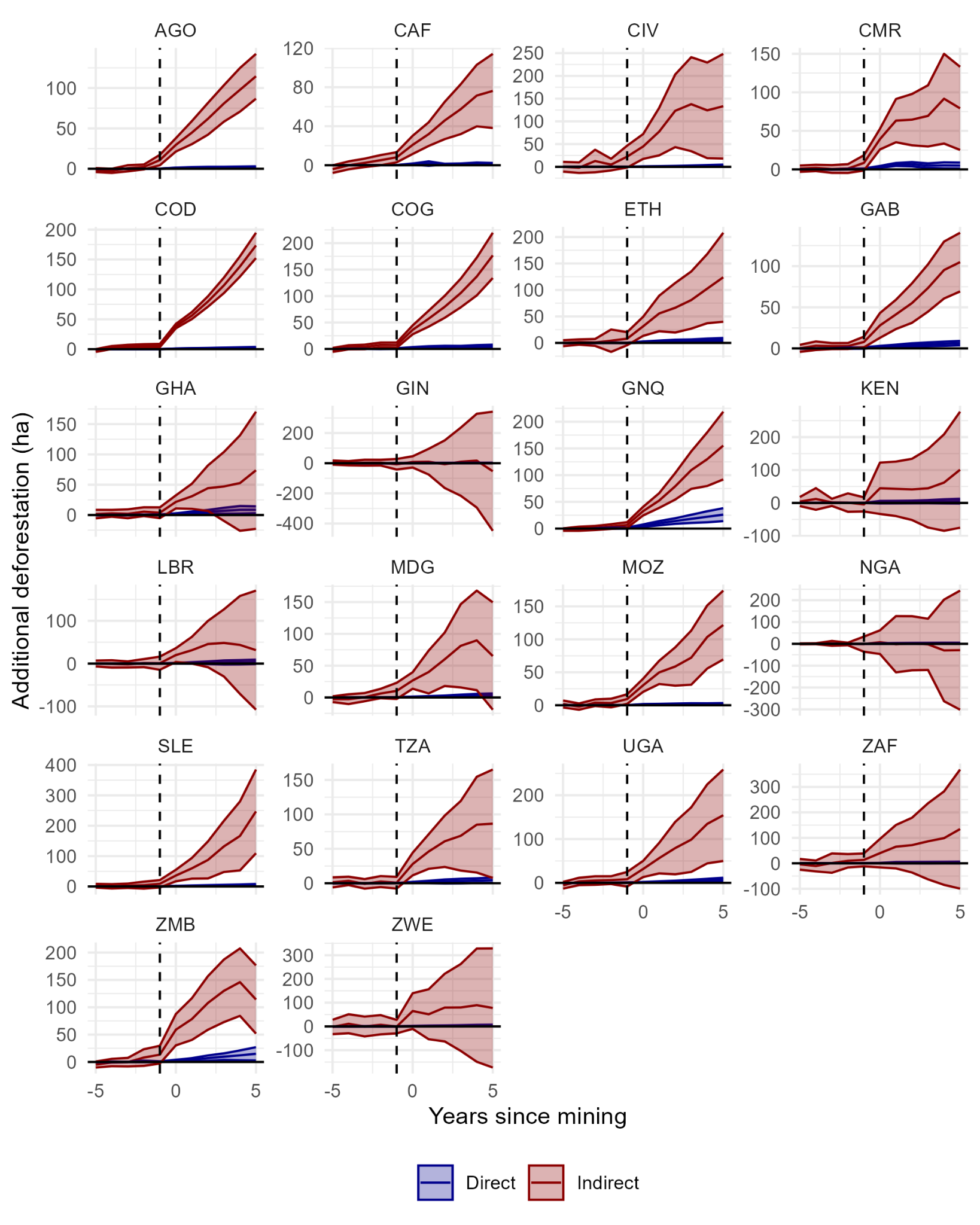
**

**Supplementary Figure 11. Direct deforestation attributed to mining and offsite deforestation (e.g. agriculture, infrastructure etc.) after mining detection.** Each panel represents the national direct and offsite deforestation estimates attributable to mining. Note - the direct loss for GNB failed to converge so is not shown.


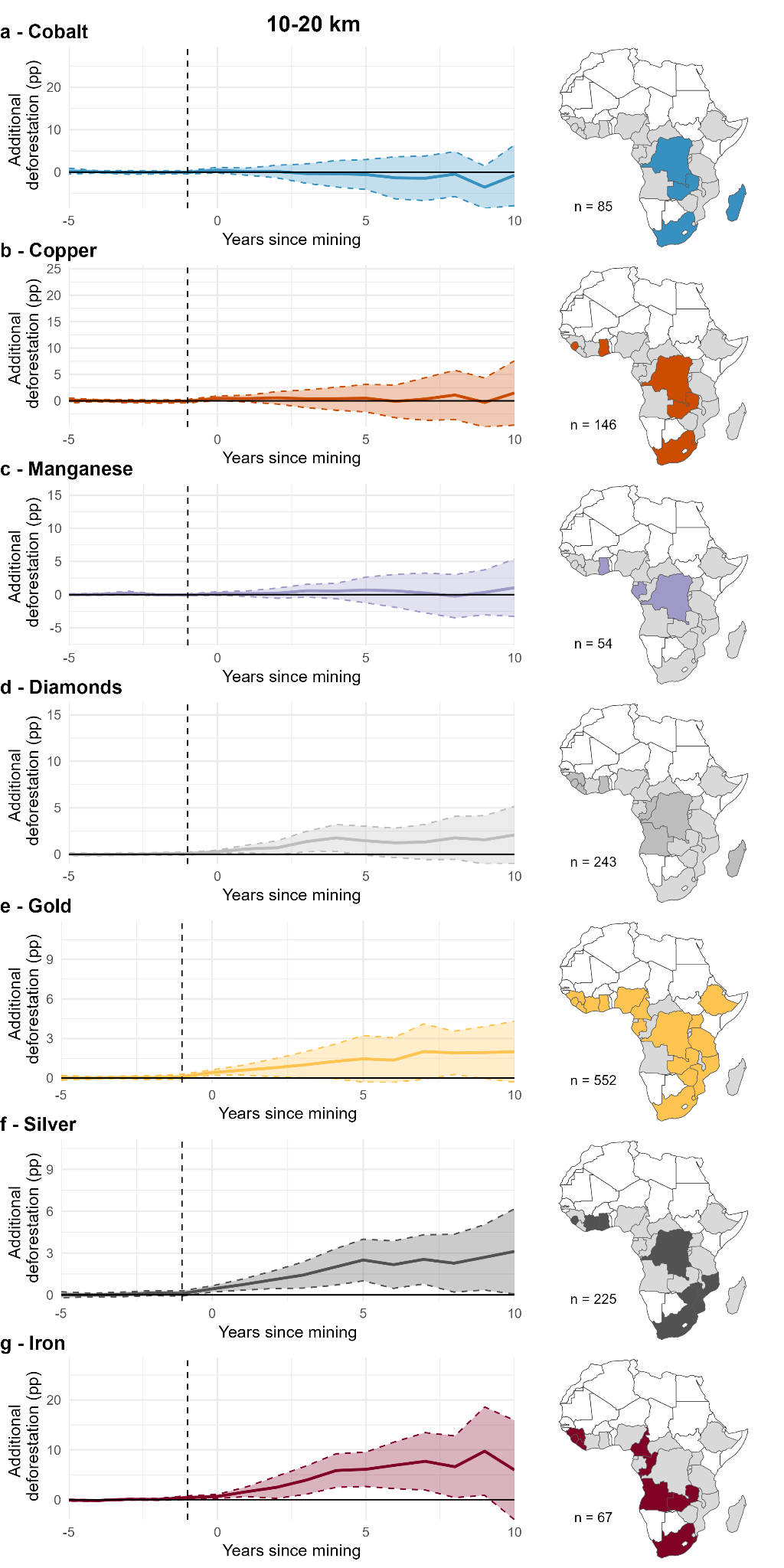


**Supplementary Figure 12. Estimated additional deforestation per key commodity in the 10-20 km concentric ring buffer.** Additional percentage points of deforestation detected in a 10-20 km concentric buffer ring around mines extracting cobalt (a), copper (b), manganese (c), diamonds (d), gold (e), silver (f), and iron (g). Solid lines denote the mean additional deforestation and the dashed ribbon the 95% confidence interval. Maps show countries containing the commodity per row in colour and all included countries in grey. Inset text denotes the sample size expressed as the number of distinct mining clusters per commodity.


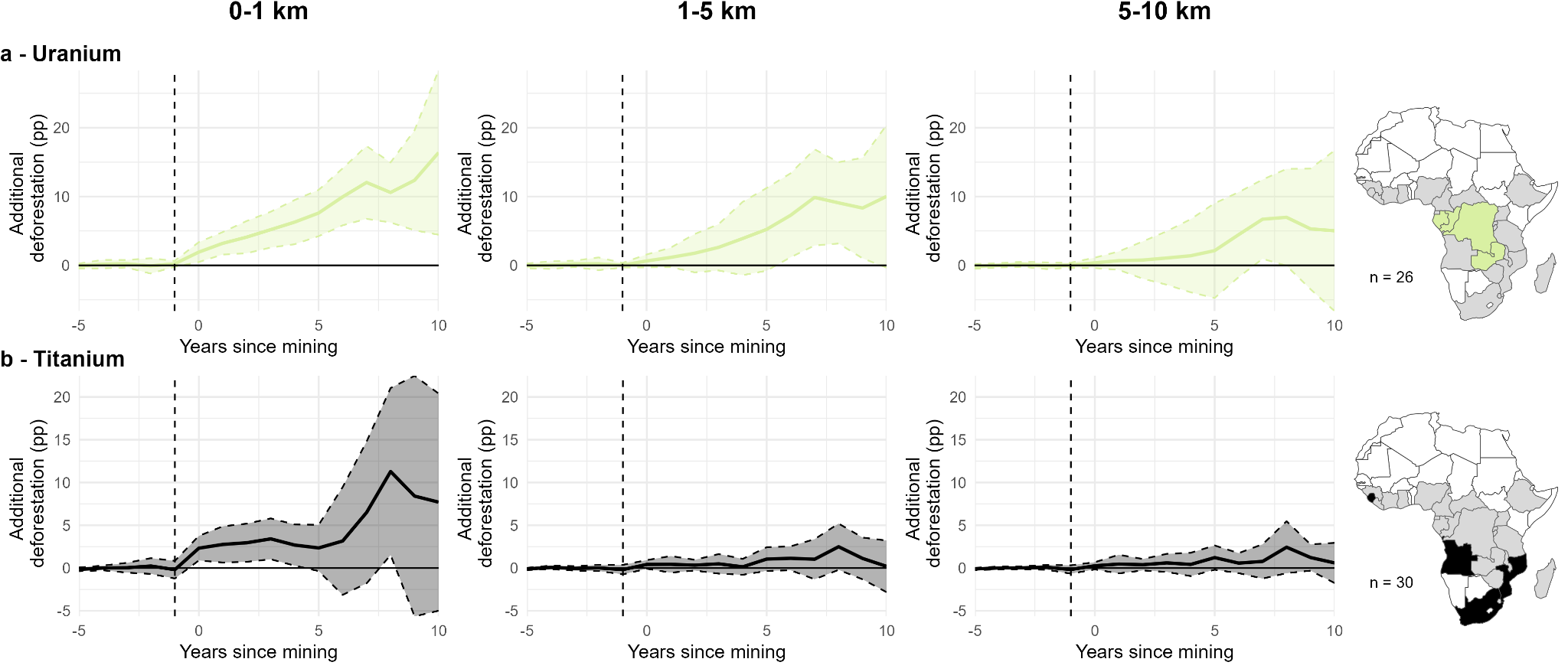


**Supplementary Figure 13. Estimated additional deforestation for uranium and titanium across space and time in Sub-Saharan Africa.** a - additional percentage points of deforestation after uranium mining is detected in 0 - 1 km, 1 - 5 km and 5 -10 km concentric buffer rings. Solid lines denote the mean additional deforestation and the dashed ribbon the 95% confidence interval. Maps show countries containing the commodity per row in colour and all included countries in grey. Inset text denotes the sample size expressed as the number of distinct mining clusters per commodity. B – shows the same for titanium.

**
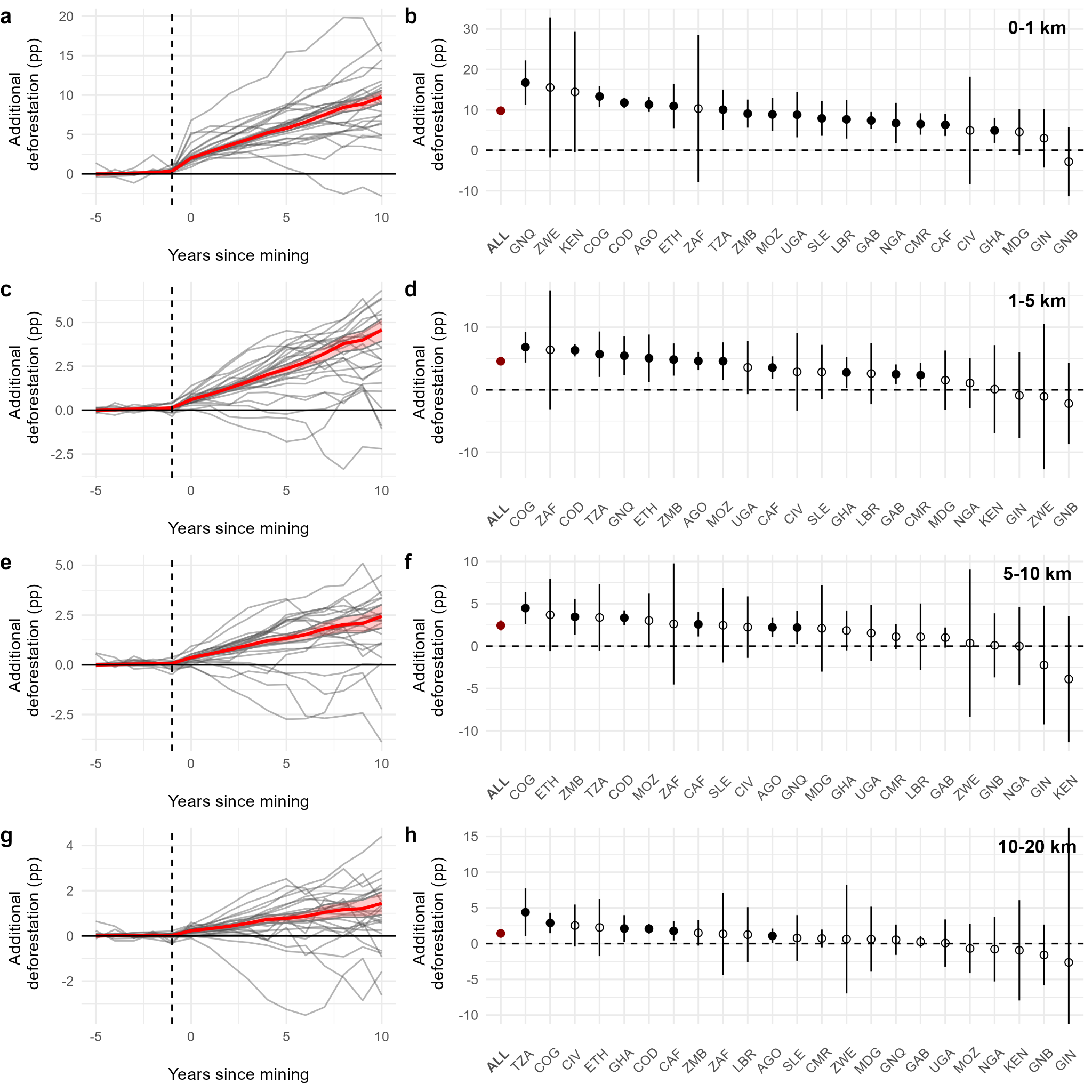
**

**Supplementary Figure 14. Estimated additional deforestation since mining is detected, across space and time in Sub-Saharan Africa after additionally dropping mines detected in 2001/2002.** Additional percentage points (pp) of deforestation in the 0 - 1 km (a), 1 - 5 km (c), 5 -10 km (e), and 10 - 20 km (g) buffers. Individual country mean estimates are shown in grey and the sub-Saharan Africa wide mean estimates and 95% confidence interval (CI) are shown in red and pale red, respectively. Summary of estimated additional pp of deforestation after 10-years for all included countries (black) and the sub-Saharan Africa wide estimate (red) in the 0 - 1 km (a), 1 - 5 km (c), 5 -10 km (e), and 10 - 20 km (g) buffers. Closed circles denote a statistically significant effect; open circles denote statistically non-significant effects. Points are mean ATTs and error bars are 95% confidence intervals. See Supplementary Table 2 for a full list of 3 letter ISO codes and their corresponding country name in full. Note the uncertainty for GIN extends beyond the shown scale.

**
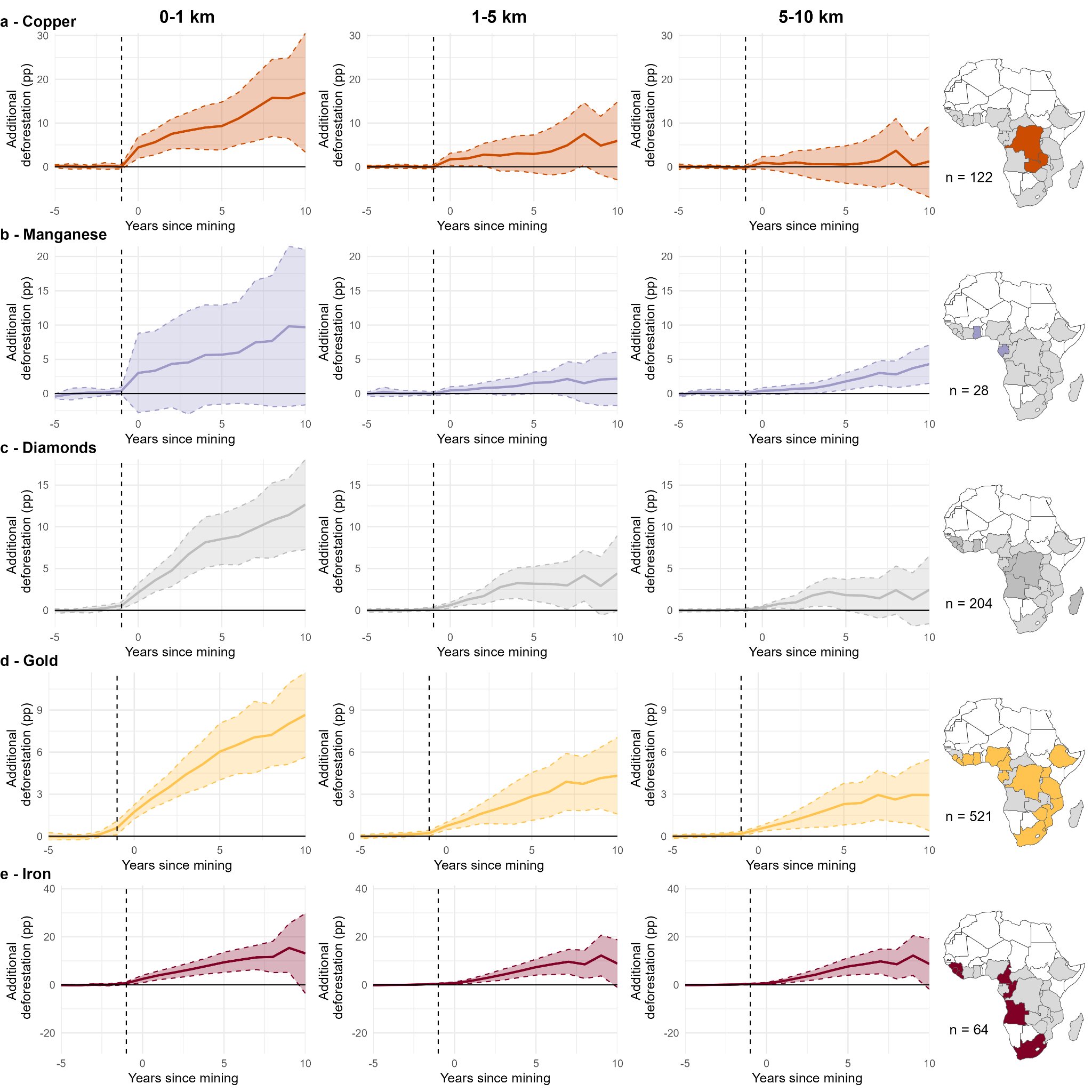
**

**Supplementary Figure 15. Estimated additional deforestation per key primary commodity across space and time in sub-Saharan Africa.** a - additional percentage points of deforestation after copper mining is detected in 0 - 1 km, 1 - 5 km and 5 -10 km concentric buffer rings. Solid lines denote the mean additional deforestation and the dashed ribbon the 95% confidence interval. Maps show countries containing the commodity per row in colour and all included countries in grey. Inset text denotes the sample size expressed as the number of distinct mining clusters per commodity. Subsequent rows show the same for mines extracting manganese (b), diamonds (c), gold (d), and iron (e).

**
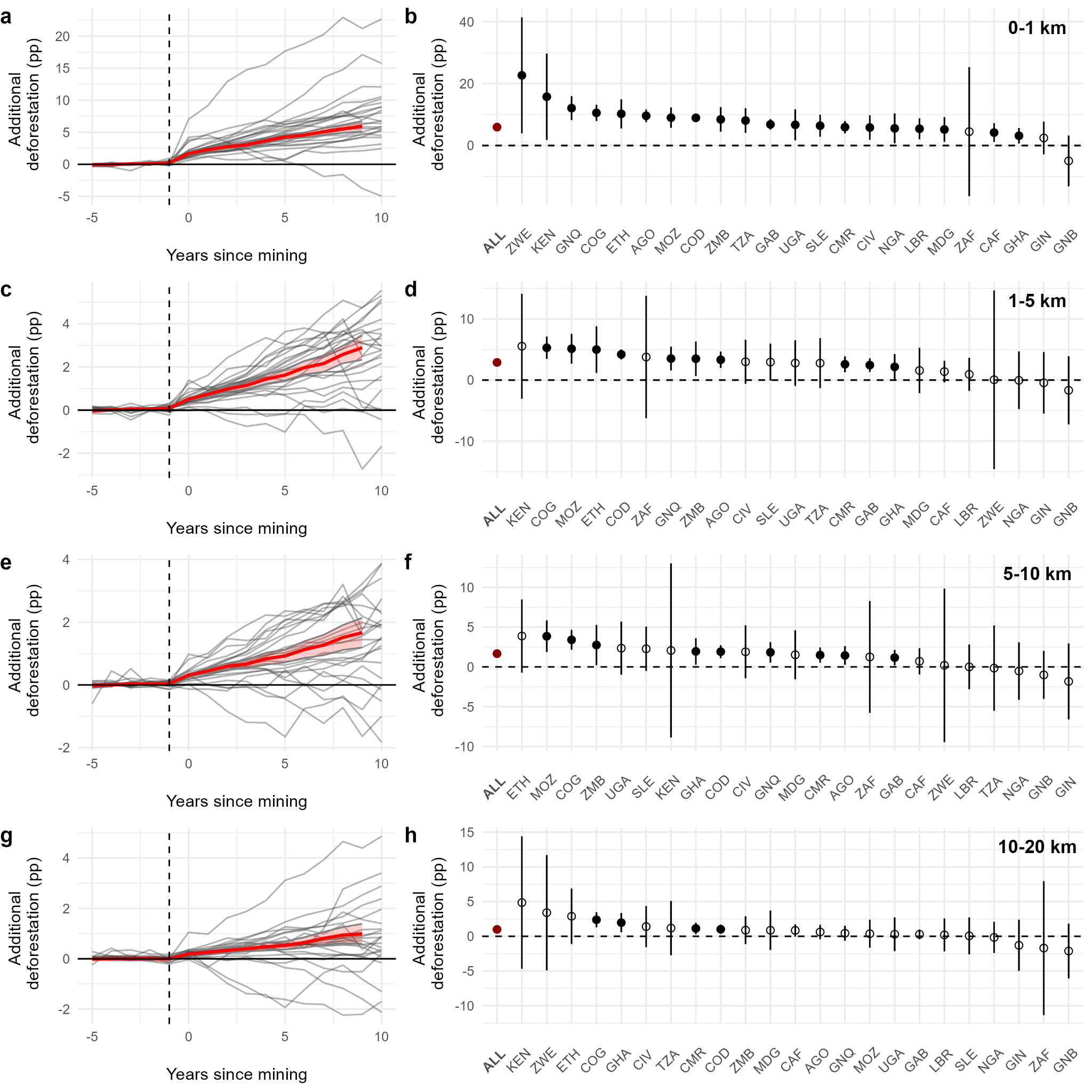
**

**Supplementary Figure 16. Estimated additional deforestation since mining is detected, across space and time in Sub-Saharan Africa using the Callaway and Sant’Anna estimator assuming a single pixel threshold for the commencement of mining.** Additional percentage points (pp) of deforestation in the 0 - 1 km (a), 1 - 5 km (c), 5 -10 km (e), and 10 - 20 km (g) buffers. Individual country mean estimates are shown in grey and the sub-Saharan Africa wide mean estimates and 95% confidence interval (CI) are shown in red and pale red, respectively. Summary of estimated additional pp of deforestation after 10-years for all included countries (black) and the sub-Saharan Africa wide estimate (red) in the 0 - 1 km (a), 1 - 5 km (c), 5 -10 km (e), and 10 - 20 km (g) buffers. Closed circles denote a statistically significant effect; open circles denote statistically non-significant effects. Points are mean ATTs and error bars are 95% confidence intervals. See Supplementary Table 2 for a full list of 3 letter ISO codes and their corresponding country name in full.

**
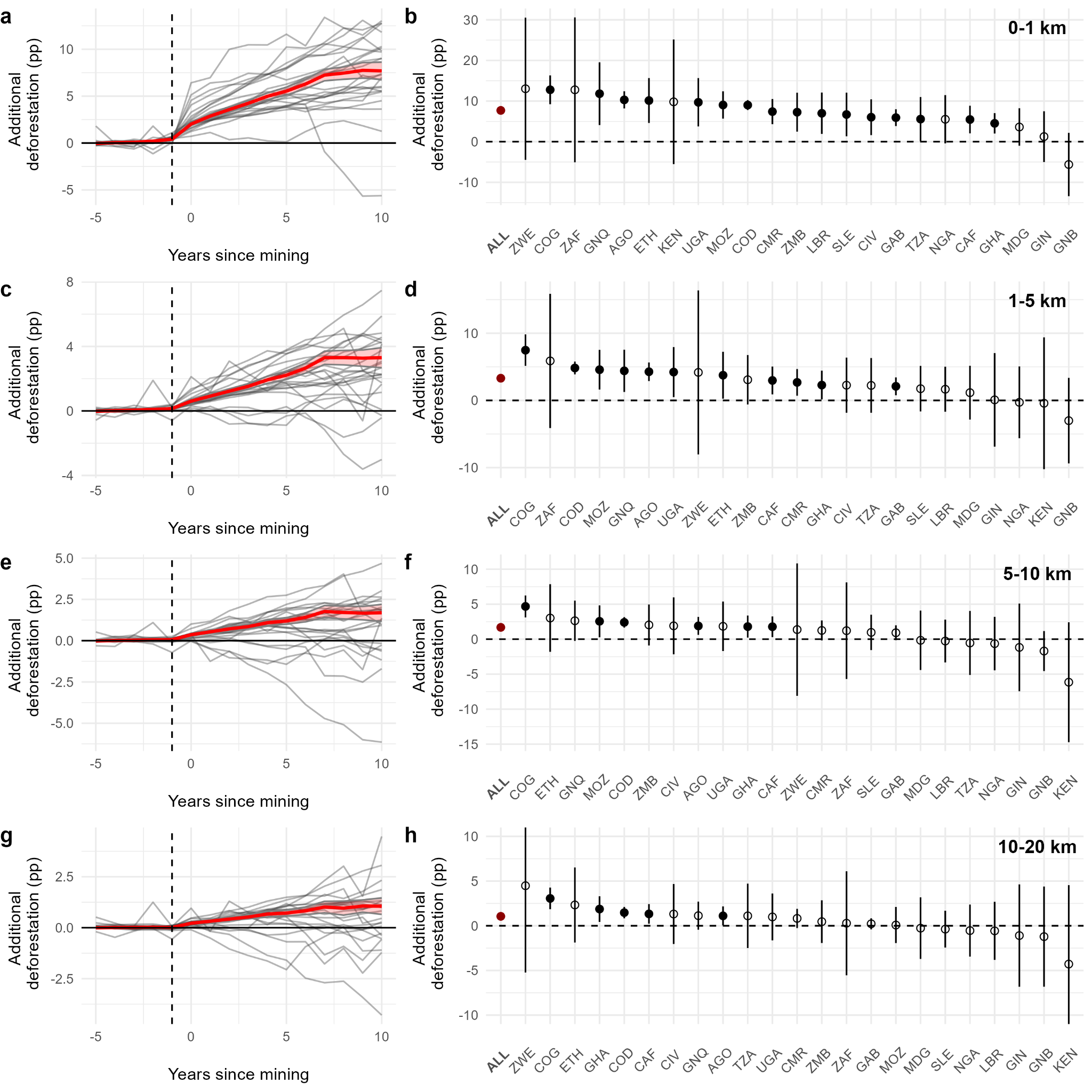
**

**Supplementary Figure 17. Estimated additional deforestation since mining is detected, across space and time in Sub-Saharan Africa using the Callaway and Sant’Anna estimator assuming a 20% threshold for the commencement of mining.** Additional percentage points (pp) of deforestation in the 0 - 1 km (a), 1 - 5 km (c), 5 -10 km (e), and 10 - 20 km (g) buffers. Individual country mean estimates are shown in grey and the sub-Saharan Africa wide mean estimates and 95% confidence interval (CI) are shown in red and pale red, respectively. Summary of estimated additional pp of deforestation after 10-years for all included countries (black) and the sub-Saharan Africa wide estimate (red) in the 0 - 1 km (a), 1 - 5 km (c), 5 -10 km (e), and 10 - 20 km (g) buffers. Closed circles denote a statistically significant effect; open circles denote statistically non-significant effects. Points are mean ATTs and error bars are 95% confidence intervals. See Supplementary Table 2 for a full list of 3 letter ISO codes and their corresponding country name in full.

**
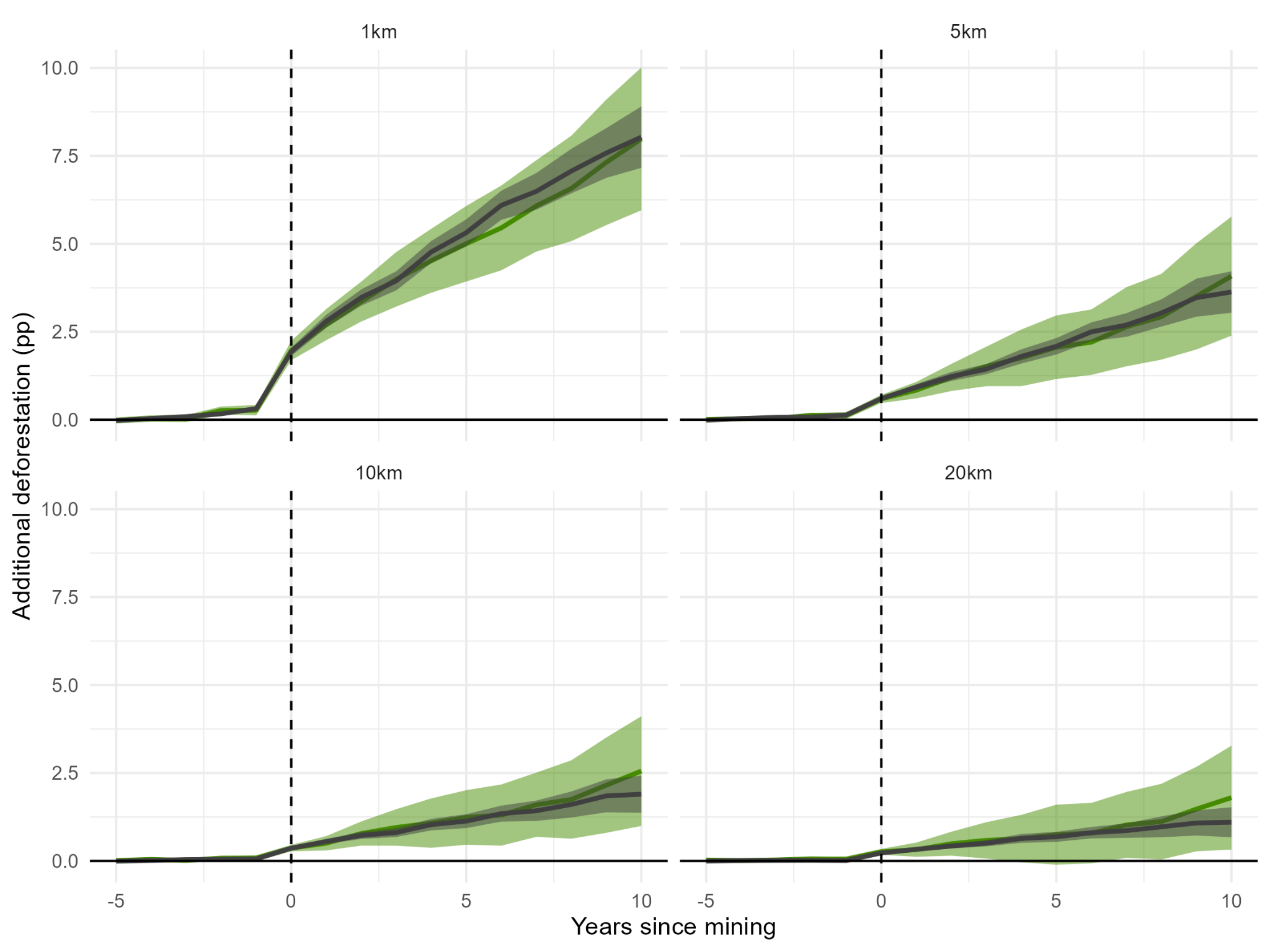
**

**Supplementary Figure 18. Estimated additional deforestation since mining is detected across SSA using our derived clusters from the pan-Africa Masolele et al., data (n = 15,477) compared to an analysis using only mines identified per Maus et al., (n = 2504).** Additional percentage points (pp) of deforestation in the 0 - 1 km, 1 - 5 km, 5 -10 km, and 10 - 20 km buffers. Black lines and ribbons refer to the full cluster-based data, green lines and ribbon denote the analysis based solely on the clusters identified in Maus et al. Solid lines denote means and shaded bands the 95% confidence interval.

**
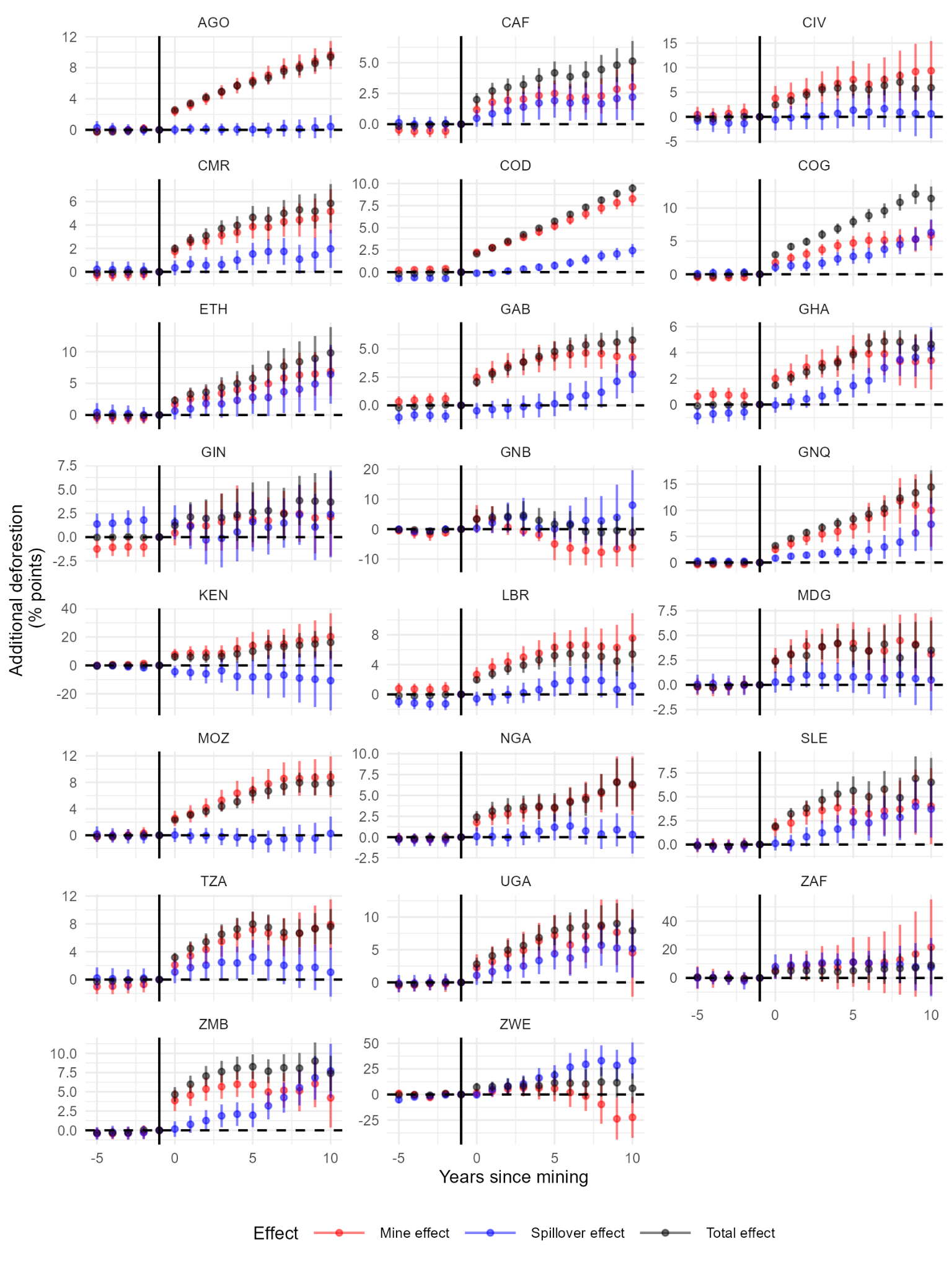
**

**Supplementary Figure 19.** **Sensitivity analysis accounting for spatial spillover from nearby mines through time in the 0 – 1 km concentric ring buffer using an extension of the Gardner (2022) 2-stage estimator.** Points are means and vertical error bars are the 95% confidence intervals.

**
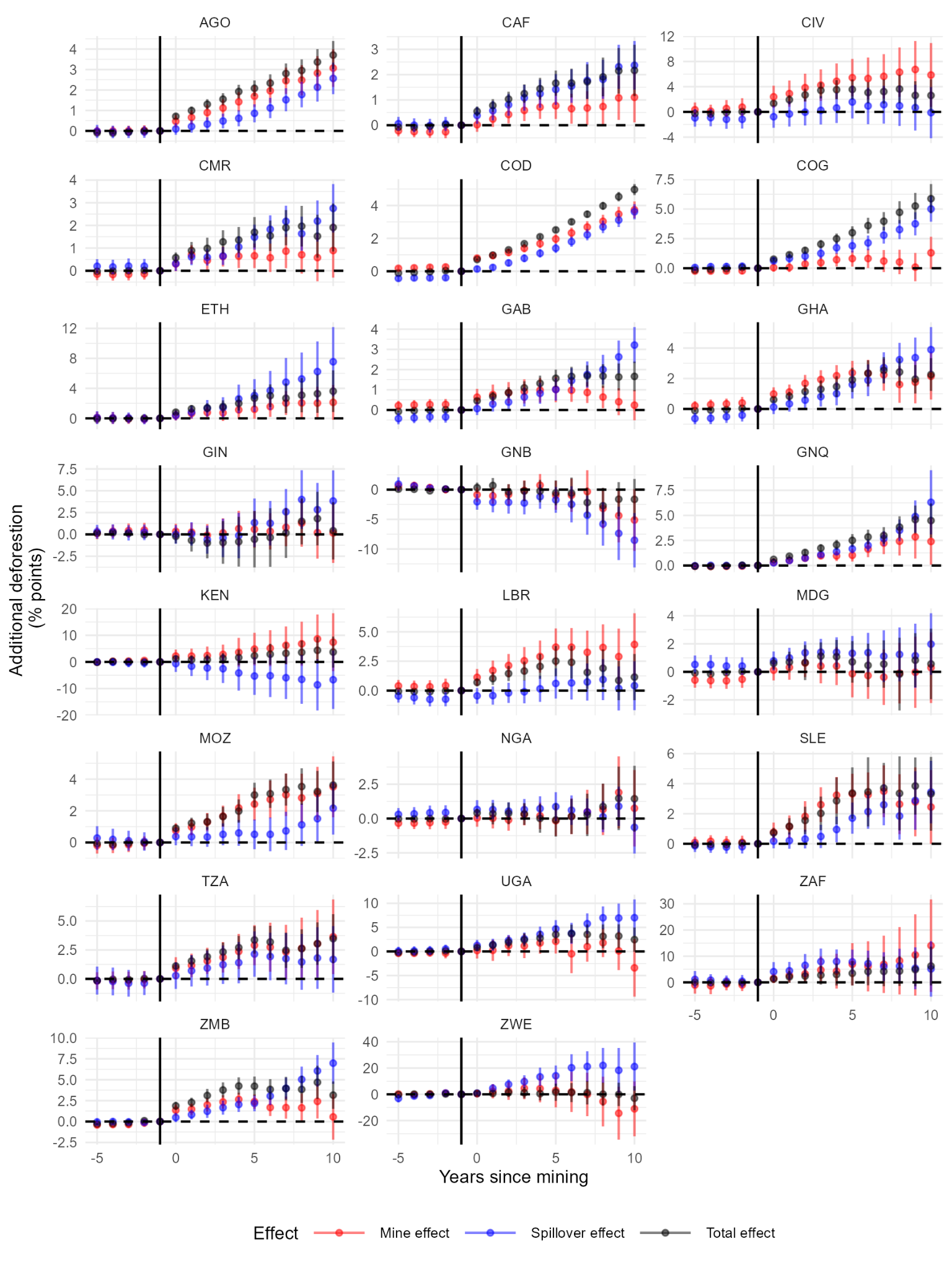
**

**Supplementary Figure 20.** **Sensitivity analysis accounting for spatial spillover from nearby mines through time in the 1 – 5 km concentric ring buffer using an extension of the Gardner (2022) 2-stage estimator.** Points are means and vertical error bars are the 95% confidence intervals.

**
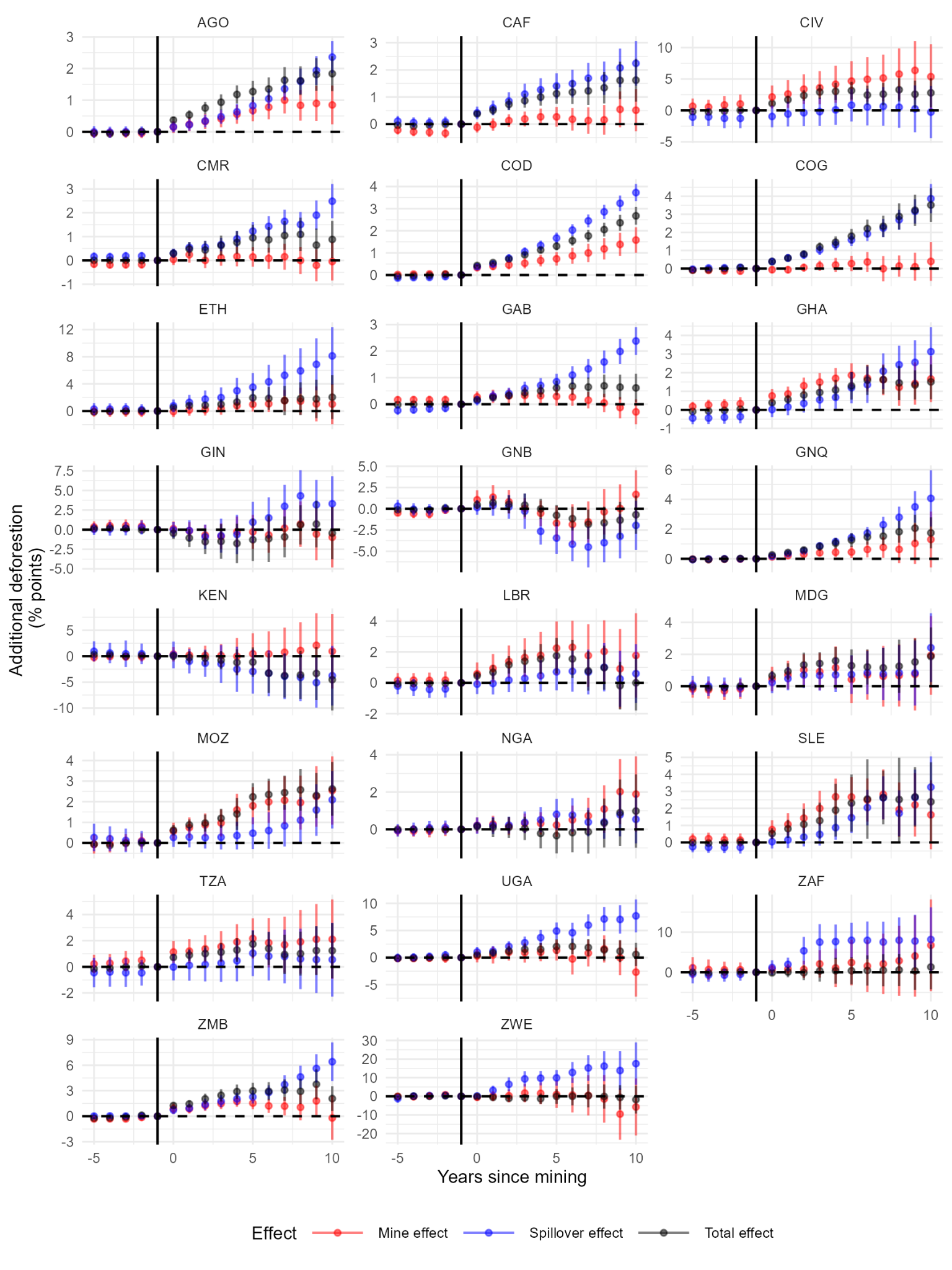
**

**Supplementary Figure 21.** **Sensitivity analysis accounting for spatial spillover from nearby mines through time in the 5 – 10 km concentric ring buffer using an extension of the Gardner (2022) 2-stage estimator.** Points are means and vertical error bars are the 95% confidence intervals.

**
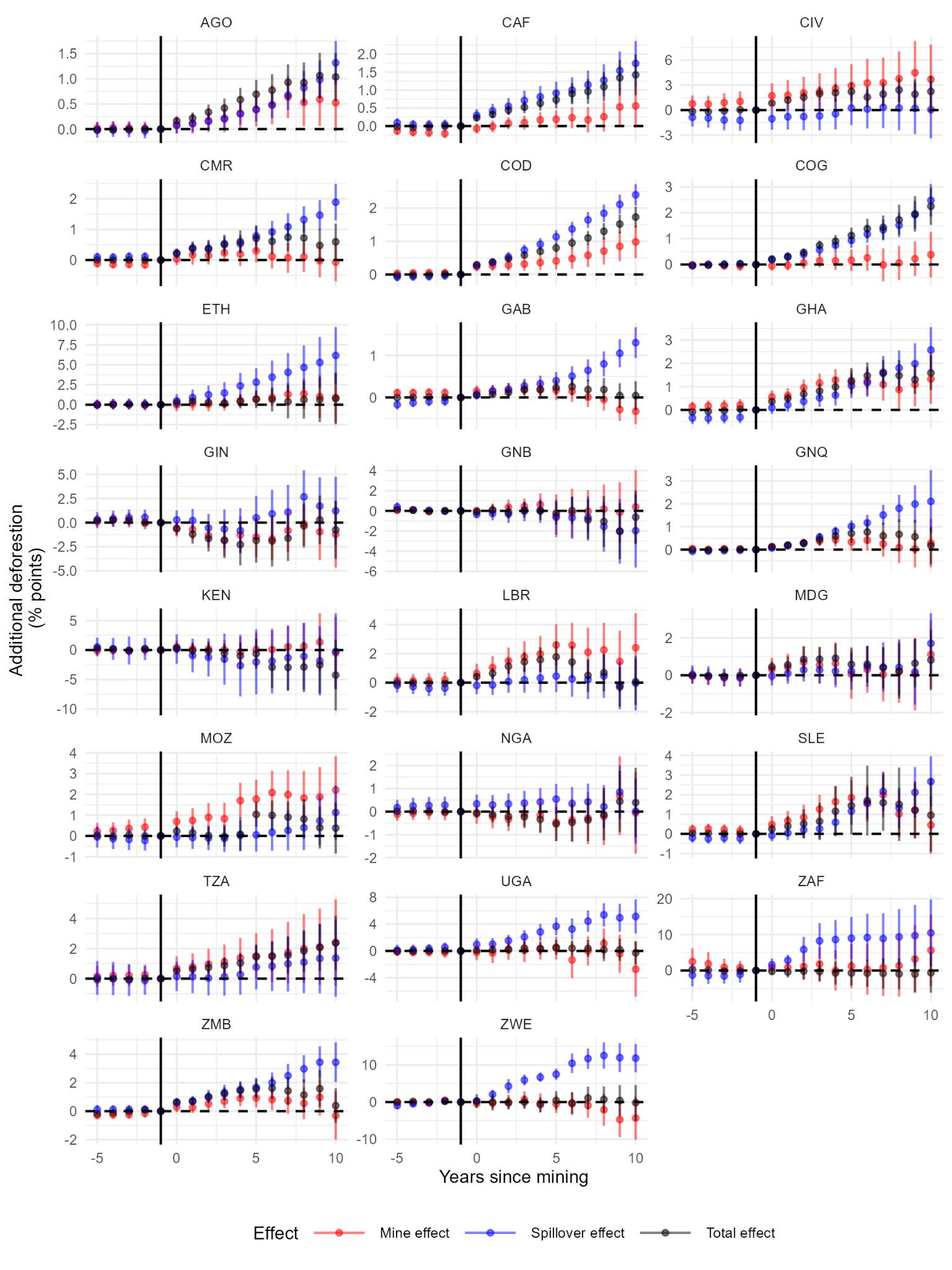
**

**Supplementary Figure 22.** **Sensitivity analysis accounting for spatial spillover from nearby mines through time in the 10 – 20 km concentric ring buffer using an extension of the Gardner (2022) 2-stage estimator.** Points are means and vertical error bars are the 95% confidence intervals.


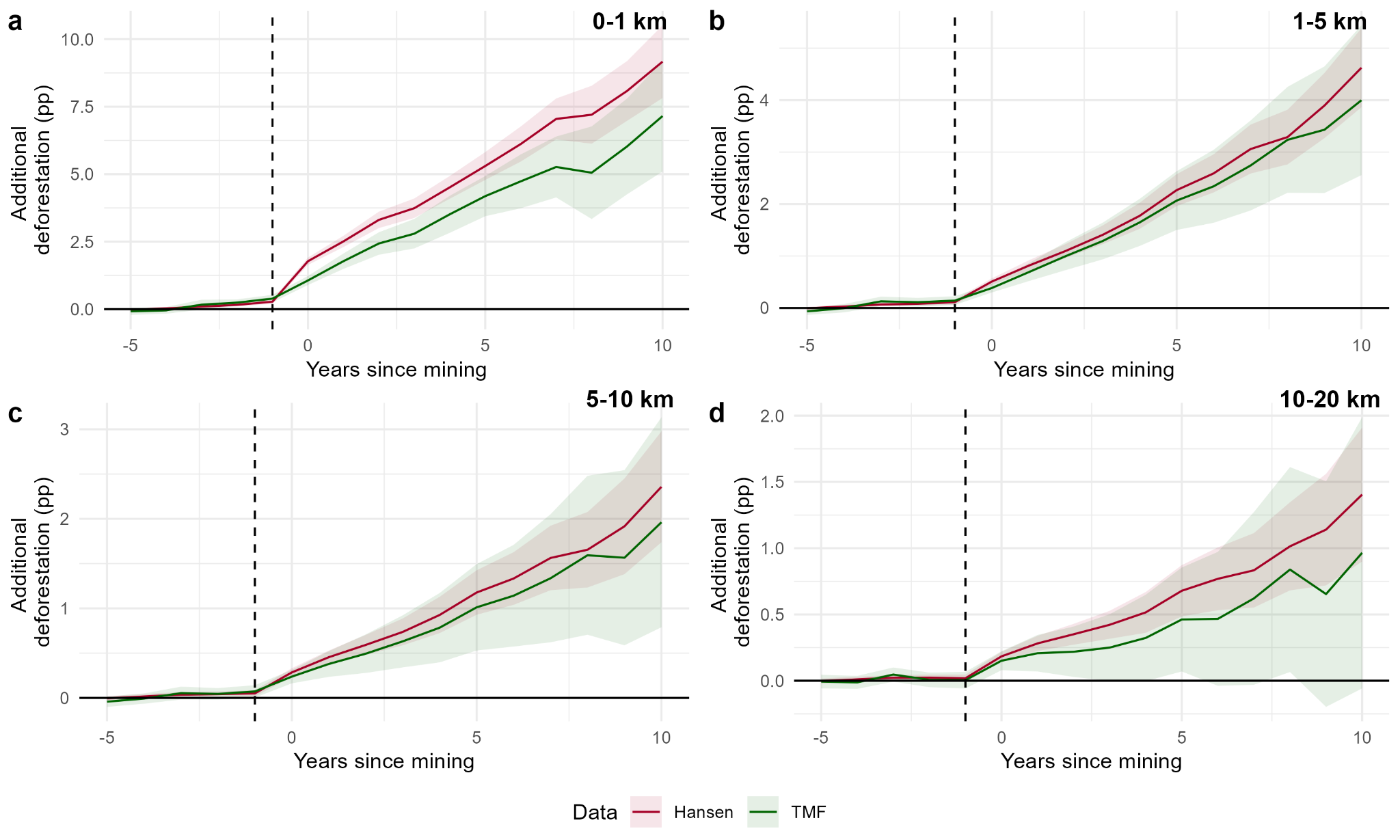


**Supplementary Figure 23. Sensitivity analysis comparing the Hansen et al., and JRC TMF forest loss data, restricted to the tropical moist forest biome.** Solid lines denote the mean additional deforestation and the dashed ribbon the 95% confidence interval.

**Supplementary Table 1. National estimates of direct mine-induced deforestation.** Shown are the total mine-induced deforestation (defined as losses in areas >50% tree cover) between 2001 and 2020, the total proportion of pan-African mining deforestation accounted for by each country, and the total proportion of national deforestation due to mining.

| **Country** | **Direct Mining Deforestation (hectares)** | **Share of African Direct Mining Deforestation (%)** | **Share of national deforestation due to mining (%)** |
| --- | --- | --- | --- |
| Angola | 13,057 | 6.98 | 0.69 |
| Benin | 28 | 0.02 | 0.41 |
| Botswana | 2 | <0.01 | 4.55 |
| Burundi | 161 | 0.09 | 1.29 |
| Cabo Verde | < 1 | <0.01 | 1.88 |
| Cameroon | 9,819 | 5.25 | 0.72 |
| Central African Republic | 1,673 | 0.89 | 0.25 |
| Chad | 0.4 | <0.01 | 0.03 |
| Comoros | 135 | 0.07 | 3.79 |
| Côte d'Ivoire | 6,268 | 3.35 | 0.33 |
| DRC | 39,044 | 20.82 | 0.26 |
| Equatorial Guinea | 5,745 | 3.07 | 4.91 |
| Ethiopia | 812 | 0.43 | 0.30 |
| Gabon | 3,315 | 1.77 | 0.76 |
| Gambia | <1 | <0.01 | 4.25 |
| Ghana | 28,144 | 15.04 | 3.21 |
| Guinea-Bissau | 486 | 0.26 | 0.76 |
| Guinea | 501 | 0.27 | 0.09 |
| Kenya | 807 | 0.43 | 0.31 |
| Lesotho | 11 | <0.01 | 44.37 |
| Liberia | 3,934 | 2.1 | 0.21 |
| Madagascar | 17,593 | 9.4 | 0.52 |
| Malawi | 393 | <0.01 | 0.50 |
| Mali | < 1 | <0.01 | 0.02 |
| Mozambique | 7,818 | 4.18 | 0.44 |
| Namibia | 6 | <0.01 | 1.77 |
| Nigeria | 1,545 | 0.83 | 0.36 |
| Republic of the Congo | 4,656 | 2.49 | 0.60 |
| Rwanda | 300 | 0.16 | 1.46 |
| São Tomé and Príncipe | 4 | <0.01 | 7.28 |
| Senegal | 12 | <0.01 | 2.97 |
| Sierra Leone | 4,608 | 2.46 | 0.35 |
| Somalia | 3 | <0.01 | 0.19 |
| South Africa | 13,723 | 7.33 | 1.48 |
| South Sudan | 24 | 0.01 | 0.04 |
| Eswatini | 1,529 | 0.82 | 1.78 |
| Tanzania | 5,283 | 2.82 | 0.40 |
| Togo | 4 | <0.01 | 0.03 |
| Uganda | 4,568 | 2.44 | 0.71 |
| Zambia | 9,908 | 5.30 | 1.26 |
| Zimbabwe | 1,145 | 0.61 | 0.99 |

**Supplementary Table 2. Table of ISO-3 codes used throughout.**

| **ISO-3 code** | **Country** |
| --- | --- |
| AGO | Angola |
| CAF | Central African Republic |
| CIV | Côte d’Ivoire |
| CMR | Cameroon |
| COD | Democratic Republic of the Congo |
| COG | Republic of the Congo |
| ETH | Ethiopia |
| GAB | Gabon |
| GHA | Ghana |
| GIN | Guinea |
| GNB | Guinea-Bissau |
| GNQ | Equatorial Guinea |
| KEN | Kenya |
| LBR | Liberia |
| MDG | Madagascar |
| MOZ | Mozambique |
| NGA | Nigeria |
| SLE | Sierra Leone |
| SWZ | Eswatini |
| TZA | Tanzania |
| UGA | Uganda |
| ZAF | South Africa |
| ZMB | Zambia |
| ZWE | Zimbabwe |

**Supplementary References**

1. B. Callaway, P. H. C. Sant’Anna, Difference-in-Differences with multiple time periods. *Journal of Econometrics* **225**, 200–230 (2021).

2. J. Gardner, Two-stage differences in differences. arXiv [Preprint] (2022). https://doi.org/10.48550/ARXIV.2207.05943.

3. C. Wing, S. Freedman, A. Hollingsworth, “Stacked Difference-in-Differences” (NBER Working Paper No. w32054, 2024); https://papers.ssrn.com/sol3/papers.cfm?abstract_id=4702247.

4. V. Maus, S. Giljum, D. M. da Silva, J. Gutschlhofer, R. P. da Rosa, S. Luckeneder, S. L. B. Gass, M. Lieber, I. McCallum, An update on global mining land use. *Sci Data* **9**, 433 (2022).

5. R. N. Masolele, D. Marcos, V. De Sy, I.-O. Abu, J. Verbesselt, J. Reiche, M. Herold, Mapping the diversity of land uses following deforestation across Africa. *Sci Rep* **14**, 1681 (2024).

6. K. Butts, Difference-in-Differences Estimation with Spatial Spillovers. arXiv [Preprint] (2021). https://doi.org/10.48550/ARXIV.2105.03737.
