# Supplementary material for: Mining triggers extensive additional deforestation in sub-Saharan Africa
Source: Nature. 2026 Jun 3;654(8120):971–7. doi: 10.1038/s41586-026-10551-2 (PMC13293856; doi:10.1038/s41586-026-10551-2)
Supplement: Supplementary file 2 — Reporting Summary [file 41586_2026_10551_MOESM2_ESM.pdf]

nature portfolio

Corresponding author(s): Oscar Morton, Christopher Bousfield, David Edwards

Last updated by author(s): 2026/03/27

Reporting Summary

Nature Portfolio wishes to improve the reproducibility of the work that we publish. This form provides structure for consistency and transparency in reporting. For further information on Nature Portfolio policies, see our Editorial Policies and the Editorial Policy Checklist.

Statistics

For all statistical analyses, confirm that the following items are present in the figure legend, table legend, main text, or Methods section.

n/a	Confirmed	
☐	☒	The exact sample size (n) for each experimental group/condition, given as a discrete number and unit of measurement
☒	☐	A statement on whether measurements were taken from distinct samples or whether the same sample was measured repeatedly
☒	☐	The statistical test(s) used AND whether they are one- or two-sided<br>*Only common tests should be described solely by name; describe more complex techniques in the Methods section.*
☐	☒	A description of all covariates tested
☒	☐	A description of any assumptions or corrections, such as tests of normality and adjustment for multiple comparisons
☐	☒	A full description of the statistical parameters including central tendency (e.g. means) or other basic estimates (e.g. regression coefficient) AND variation (e.g. standard deviation) or associated estimates of uncertainty (e.g. confidence intervals)
☒	☐	For null hypothesis testing, the test statistic (e.g. F, t, r) with confidence intervals, effect sizes, degrees of freedom and P value noted<br>*Give P values as exact values whenever suitable.*
☒	☐	For Bayesian analysis, information on the choice of priors and Markov chain Monte Carlo settings
☐	☒	For hierarchical and complex designs, identification of the appropriate level for tests and full reporting of outcomes
☐	☒	Estimates of effect sizes (e.g. Cohen's d, Pearson's r), indicating how they were calculated

Our web collection on statistics for biologists contains articles on many of the points above.

Software and code

Policy information about availability of computer code

Data collection	All code for the analysis is available at: https://github.com/OMorton/AFR_MiningForestLoss.
Data analysis	All code for the analysis is available at: https://github.com/OMorton/AFR_MiningForestLoss.

For manuscripts utilizing custom algorithms or software that are central to the research but not yet described in published literature, software must be made available to editors and reviewers. We strongly encourage code deposition in a community repository (e.g. GitHub). See the Nature Portfolio guidelines for submitting code & software for further information.

Data

Policy information about availability of data

All manuscripts must include a data availability statement. This statement should provide the following information, where applicable:

- Accession codes, unique identifiers, or web links for publicly available datasets
- A description of any restrictions on data availability
- For clinical datasets or third party data, please ensure that the statement adheres to our policy

All data used in this study is freely available for download online. The post-deforestation land use data from Masolele et al. (2024) is available at: https://zenodo.org/records/11065705, the tree cover and forest loss data is available at: https://storage.googleapis.com/earthenginepartners-hansen/GFC-2023-v1.11/download.html , the plantation data from the SDPTv2 is available at: https://www.globalforestwatch.org/blog/data-and-tools/updated-planted-trees-map-near-global-coverage/. The processed data outputs needed to run the analyses are available at https://github.com/OMorton/AFR_MiningForestLoss.

Research involving human participants, their data, or biological material

Policy information about studies with human participants or human data. See also policy information about sex, gender (identity/presentation), and sexual orientation and race, ethnicity and racism.

Reporting on sex and gender	NA
Reporting on race, ethnicity, or other socially relevant groupings	NA
Population characteristics	NA
Recruitment	NA
Ethics oversight	NA

Note that full information on the approval of the study protocol must also be provided in the manuscript.

Field-specific reporting

Please select the one below that is the best fit for your research. If you are not sure, read the appropriate sections before making your selection.

☐ Life sciences ☐ Behavioural & social sciences ☒ Ecological, evolutionary & environmental sciences

For a reference copy of the document with all sections, see nature.com/documents/nr-reporting-summary-flat.pdf

Ecological, evolutionary & environmental sciences study design

All studies must disclose on these points even when the disclosure is negative.

Study description	The cumulative additional total deforestation (summed direct and offsite) triggered by mining was estimated using heterogeneity robust DiD methods to track deforestation in concentric ring buffers around mines before and after mining operations commence, using a 'not yet treated' quasi-experimental design (See Methods for more details).
Research sample	All pixels where forest was lost and mining was determined as the end use of that land (Masolele et al., 2024). All data used in this study is freely available for download online. The post-deforestation land use data from Masolele et al. (2024) is available at: https://zenodo.org/records/11065705.
Sampling strategy	NA
Data collection	All data are publicly availably spatial layers. One dataset requires permission to share further which is the commodity data set.
Timing and spatial scale	2000-2020, across sub-Saharan Africa, but only in countries with mines identified from our dataset that also met our forest cover criteria.
Data exclusions	Relevant exclusions were applied at the pixel level, including the removal of plantation forests and cells with forect cover but at a percentage lower than our cut-off. Please see the methods for full details of this.
Reproducibility	This was a non- experimental study. However, effort was taken to assess the robustness of our results by completing a number of supplementary analyses testing the robustness of our results to numerous reasonable alterations in our assumptions and methods. All analysis code is freely available and linked in the manuscript.
Randomization	NA
Blinding	NA

Did the study involve field work? ☐ Yes ☐ No

Field work, collection and transport

Field conditions	NA
Location	NA

Access & import/export	NA
Disturbance	NA

Reporting for specific materials, systems and methods

We require information from authors about some types of materials, experimental systems and methods used in many studies. Here, indicate whether each material, system or method listed is relevant to your study. If you are not sure if a list item applies to your research, read the appropriate section before selecting a response.

Materials & experimental systems

n/a	Involved in the study
☒	☐ Antibodies
☒	☐ Eukaryotic cell lines
☒	☐ Palaeontology and archaeology
☒	☐ Animals and other organisms
☒	☐ Clinical data
☒	☐ Dual use research of concern
☒	☐ Plants

Methods

n/a	Involved in the study
☒	☐ ChIP-seq
☒	☐ Flow cytometry
☒	☐ MRI-based neuroimaging

Plants

Seed stocks	NA
Novel plant genotypes	NA`
Authentication	NA
