# Supplementary material for: Mining triggers extensive additional deforestation in sub-Saharan Africa
Source: Nature. 2026 Jun 3;654(8120):971–7. doi: 10.1038/s41586-026-10551-2 (PMC13293856; doi:10.1038/s41586-026-10551-2)
Supplement: Supplementary file 3 — Peer Review File [file 41586_2026_10551_MOESM3_ESM.pdf]

nature portfolio

Peer Review File

Mining triggers extensive additional deforestation in sub-Saharan Africa

Corresponding Author: Dr Oscar Morton

Any redactions in this file are there to maintain patient confidentiality, the confidentiality of unpublished data, or to remove third-party material.

This file contains all reviewer reports in order by version, followed by all author rebuttals in order by version.

Version 0:

Reviewer comments:

Referee #1

(Remarks to the Author)
I was asked to review this manuscript focusing particularly on the 'remote sensing based estimates of deforestation'.

The study is interesting and relevant. It takes advantage of an existing dataset of deforestation location and canopy cover (Hansen et al 2013, whose dataset has been updated annually since), and Masolele et al (2024) which attributed Hansen-detected deforestation events in Africa to particular sources, including mining, in order to do an elegant difference-in-difference analysis to detect whether mining activities cause additional local deforestation. The use of mine sites prior to start date as controls is possible thanks to the very large number of mines analysed, and means the authors can avoid problems of attempting to find suitable matching sites for each mine, that has caused major issues for previous studies of e.g. national park or REDD+ project implementations, tested against similar datasets.

Their findings are unsurprising - firstly that mining causes direct deforestation (because of their methods, mines that did not cause deforestation would not even come up in their analysis, so this is a given); and that mining triggers increased deforestation within 1km of the mine site (a lot of which will be direct infrastructure for the mine and related to increased workers in the area - interesting but unsurprising). But giving quantitative figures (34 hectares forest loss for every 1 hectare of mining loss) to these is useful given how data deficient we are on mining impacts in sub-Saharan Africa. These figures are sufficiently useful for science, conservation and mining impact mitigation plans to justify publication from an interest standpoint.

The more interesting/surprising finding, that deforestation remains slightly elevated up to 20km from mines compared to background, is again useful. I have slightly more concerns about the degree to which this is real, given the background increase in deforestation rates in the key regions (ideally for this analysis deforestation rates country-wide would remain flat, so that before- and after-impact were against the same background). However, the authors have included sensible and comprehensive uncertainty estimation methods, and done various alternative analysis methods and sensitivity analyses - making me convinced the analysis is robust within its confidence ranges.

The findings are highly dependent on the accuracy of the deforestation detection in Hansen et al. (2013), and its attribution in the Malolele et al. (2024) paper. I know both datasets well, and have published previously on the errors and problematic use of the Hansen et al. (2013) dataset. I don't think these concerns apply here, and the datasets have been used appropriately. Issues related to inclusion of plantations in Hansen et al. have been largely covered by use of an independent plantation dataset. And the Malolele et al. dataset finds mine sites well - they have a very distinctive spectral and spatial signature, which the machine learning and ~5m Planet data picks up well.

Some mines will be missed (particularly small or low impact mines), and some deforestation will be missed. The authors account for the missing mines in their Discussion, and there is no reason why forest loss data should be more likely to miss impacts before or after mining starts, so I do not think that will cause a bias.

There is a discontinuity in the Hansen et al. data, with some deforestation missed in the 2011-13 period where there was

little Landsat data, and an increase in deforestation sensitivity after 2013 when Landsat 8 came on board. I have analysed the results graphs and have found no evidence of spikes around these dates - so I don't think this has caused a significant impact.

Overall, I am satisfied the conclusions of the paper are robust.

DETAILED COMMENT

1. Forest definition.

I find the definition of 'forest' as land with >50% forest cover very problematic. Legal definitions of forest are allowed within bounds set by the FAO and UNFCCC treaties (and IPCC reports) to vary between 10% and 30% canopy cover. Most of the relevant African countries, and recent relevant legislation/regulation (e.g. EU's EUDR) set the forest definition at 10% canopy cover.

I think the authors are really talking about something like 'dense forest', which is fine. This is what people often think of as 'forest' and picture deforestation as the loss of trees from an area that is near-closed-canopy forest. This is also the type of forest that is best captured by the Hansen dataset. It does poorly at detecting losses of low canopy cover forest, compared to high canopy cover - this is a major reason for the lower performance statistics in African countries, which have a lot of the world's low canopy cover forests.

I therefore would not suggest re-doing the analysis, but I would prefer the authors talking in the Abstract and relevant text points about 'dense forest' and 'deforestation of dense forest' or similar, rather than just defining forest in a way that does not match local or international definitions or understandings. They could also recognise more in the Discussion that for some of the countries discussed (particularly drier countries of southern and eastern Africa), they are only looking at a small subset of total forest in these countries. They are specifically looking here at mines near 'dense forest', and their impact on 'dense forest'. Mines in low canopy cover savanna that cause extensive losses of that low canopy cover forest are not looked at in this analysis. I think that's okay - but it should be pointed out.

Referee #2

(Remarks to the Author)
1) it would be useful to clarify the distinction in this work versus the work by Sonter et al. (2017). That study looked at a different region (Brazil) and used a matching approach so differs methodologically. Although you cite that study, you do not make any direct comparisons or references to it, which is surprising, given that it attempts to do the same basic assessment (determine how much deforestation in nearby areas is attributable to mining). In fact in the discussion you note "we lack regional-scale understanding… in other key biodiversity and carbon hotspots… such as the Amazon Basin…" This conclusion does not seem warranted in full.

2) The empirical strategy is reasonable in general, but also part of an active research frontier. The authors have used the basic approach in Callaway and Sant'Anna, with robustness assessment using the approach suggested by Gardner. They do not try the stacking approach suggested by Wing et al. The authors also explicitly do not conduct matching analysis, arguing that the un-mined sites would be systematically different. Although the authors include an array of robustness checks and whatnot, it would be useful to show the basic model estimates and parameters, especially for the covariates used in some of the models. I understand there are lots of models for individual countries and whatnot, so maybe only do this for the all-Africa regressions.
Given that an existing result for Brazil exists that uses a matching approach to identify the effects rather than the before/after, it would be useful to also conduct the matching analysis. This is a lot of additional work, I understand, but the identification argument here is that you can identify the increase in deforestation by comparing to not yet treated plots, but if the concern with matching is that the outside locations are known as areas without mining resources, doesn't that mean there is systematic bias in the DID identification strategy used here?

3) The commodity specific estimates are a useful innovation. Given deficiencies in the data that you note, how confident are you in comparing across these results (they are, after all, just a reflection of the main results)?

4) There needs to be more discussion about the 187,000 hectare deforestation number in the abstract. First, the number does not seem all that large given the 20 year period in question and the area. But I guess this is just the land in <1 km? Second, in Figure 3, it would be useful to present the aggregate deforestation number in tabular form for the concentric rings (<1, 1-5, etc.). COD looks enormous for instance, with perhaps >2,000,000 in rings 1-20km? In Figu 3 you should include the aggregate numbers, while int eh supplement table 1, you can do the same thing for each country.

Referee #3

(Remarks to the Author)
The topic is highly relevant and the findings are important. However, the authors make use of existing datasets to derive the patterns, without data modification or evaluation. This also means that the authors seem to fully trust the datasets from references 22 and 23, as they did not put much effort in evaluating their usability for their case. There are sensitivity analyses regarding the analytical methods, but that does not include any impact of uncertainty of the datasets used.

For this level of work, this seems to be too simple, and I would suggest to add a more thorough evaluation of the applied datasets, in particular from reference 22, and also expand the analysis by adding more own contributions. That could be, for example, more detailed information on the mining related deforestation, such as road construction, etc.

-Forest loss, also in reference 22, comes from 30-m Landsat data. That implies that both forest loss and mining areas must have a large size, missing many small ones and also missing forest loss. Are there other products that could be used to evaluate the forest loss data? What about this dataset: https://forobs.jrc.ec.europa.eu/TMF ? Maybe acquire high resolution images for a specific site and see how well the forest loss and the mining mapping are covered?

-Could the mining areas be evaluated? How does the database from Maus et al (2022) match with the mining areas of reference 22? Are there any other studies that have used higher resolution sensors to map mining areas in Africa that could be used for comparison? I noticed Supplementary Fig 9 but it is not a real evaluation of the mining area mapping. I understand that using different datasets, the conclusion remains the same, however, the conclusion that mining triggers deforestation is not entirely surprising, so the numbers are in fact important.

-I understand that for the conclusion of this work, it is less important to map mining areas wall-to-wall, but it would be important to avoid miss-classifications. However, the question should be asked: What does it imply that the mapping resolution is rather coarse and many small mining areas are missed? What about forest degradation? I think it is at least a crucial discussion point that small mining sites are not part of this work, and that only large clear-cuts are mapped as forest loss, which overlooks smaller areas and single tree removal. The numbers on deforestation are thus likely much higher than reported here.

-The mining start year is defined based on reaching 10% mining pixel coverage, but this threshold seems arbitrary? So it could be useful to test the sensitivity of this value.

- It could be good to have more detailed information on what is going on around the mining sites. What is causing these forest losses? Can you quantify the reasons for forest loss? What kind of trees are lost (maybe use canopy height as a measure to get an idea if it is old forest)? Adding information may help to reader to trust that there is in fact causality between the deforestation in the buffer and the mining activities. You could also generate a new mining dataset using NICFI images from 2018–2025, maybe just for a small area to confirm that the access roads were built for mining activities.

-Nevertheless, the question about causality should be asked. Although a pseudo-start method is used, how can you be sure the deforestation is in fact an indirect result of the mining activities?

Referee #4

(Remarks to the Author)
This paper assesses the amount of deforestation associated with mining in Sub-Saharan Africa, differentiating between direct mine-site impacts, and deforestation at different distances from the mine site. The results are presented across countries and where possible across mineral types. The results add to a growing body of literature empirically demonstrating and measuring the impacts of natural resource extraction on forest cover. They are a useful contribution for their focus on the continent.

I do not have any particular quibbles with the findings, but I am unsure what to do with them. While I appreciate that it is not the role of science to give actionable findings, in this instance I think that there is perhaps some value in speaking more cogently to policy both because the focus of the research is chosen deliberately for its relevance to and currency within contemporary policy debates, including some of those recently at the COP-30 in Belem, Brazil. Also, the discussion is framed largely in terms of policy relevance. That discussion speaks mostly to questions of improving Environmental Impact Assessments as a means of addressing deforestation risk. However, the analysis does not tell us what share of the mining considered might even be susceptible to the conduct of EIAs. The discussion refers to “artisanal” and “industrial” mining as two forms of mining in the continent. However, the analysis does not seem to distinguish between the two types of mining. Thus we are unable to know if and how patterns of deforestation on and off-site vary depending on whether the mine is an industrial mine (presumably meaning large scale machinery) or artisanal (presumably referring to mining with rustic tools. One assumes that the off-site effects of these types of mining would differ considerably too because they are associated with quite different forms of population movement, food supply needs, and associated infrastructure. Similarly, there is no distinction between whether the mines are formally registered and owned, or are non-legal mines operated without title and beyond the bounds of government regulation. Only some of these mines would even consider conducting an EIA. And even in those cases that might be candidates for EIAs, whether they do one or not depends on the quality of their governance and oversight by the state. Presumably for the patterns of deforestation identified by the authors to exist, the mines must already be failing in their EIAs and the governments failing in requiring them or holding mines to account on the basis of these prior assessments. This makes a discussion calling for better EIA’s a little bit moot.

I think that not disaggregating patterns by type of mining is also problematic because the paper refers to the impact of “mining” on deforestation when in practice it may be the impact of a particular type of mining. Which type of mining would also have implications for the discussion section, because policy and regulatory responses would presumably vary a lot for different types of mining.

Version 1:

Reviewer comments:

Referee #1

(Remarks to the Author)
I have read the response to reviewers and new draft in full, again with a focus on the remote sensing side of the paper.

I appreciate the paper now refers to 'dense forest' throughout, which is appropriate to the way they are using satellite datasets.

I am satisfied the interesting results they see are real, and not an artefact of the analysis. I have no hesitation in recommending this for publication.

Referee #2

(Remarks to the Author)
The authors have addressed my key concerns adequately including new analysis using parallel methods.

Referee #3

(Remarks to the Author)
The authors have answered my comments and those from the other reviewers. I do not have further comments on the manuscript. The method seems to be sound.

Referee #4

(Remarks to the Author)
1. I really enjoyed, and appreciated, the ways in which the authors engaged the four reviewers' comments. I imagine that some of the reviewers may still not be fully satisfied because of disagreements about preferred methodologies. But this is a very serious response, that included several new runs of the data.

2. I am quite satisfied with the response to my comments, and appreciate the text added in the Discussion.

Open Access This Peer Review File is licensed under a Creative Commons Attribution 4.0 International License, which permits use, sharing, adaptation, distribution and reproduction in any medium or format, as long as you give appropriate credit to the original author(s) and the source, provide a link to the Creative Commons license, and indicate if changes were made.
In cases where reviewers are anonymous, credit should be given to 'Anonymous Referee' and the source.
The images or other third party material in this Peer Review File are included in the article's Creative Commons license, unless indicated otherwise in a credit line to the material. If material is not included in the article's Creative Commons

license and your intended use is not permitted by statutory regulation or exceeds the permitted use, you will need to obtain permission directly from the copyright holder.
To view a copy of this license, visit https://creativecommons.org/licenses/by/4.0/

Referee #1

I was asked to review this manuscript focusing particularly on the 'remote sensing based estimates of deforestation'.

The study is interesting and relevant. It takes advantage of an existing dataset of deforestation location and canopy cover (Hansen et al 2013, whose dataset has been updated annually since), and Masolele et al (2024) which attributed Hansen-detected deforestation events in Africa to particular sources, including mining, in order to do an elegant difference-in-difference analysis to detect whether mining activities cause additional local deforestation. The use of mine sites prior to start date as controls is possible thanks to the very large number of mines analysed, and means the authors can avoid problems of attempting to find suitable matching sites for each mine, that has caused major issues for previous studies of e.g. national park or REDD+ project implementations, tested against similar datasets.

Their findings are unsurprising - firstly that mining causes direct deforestation (because of their methods, mines that did not cause deforestation would not even come up in their analysis, so this is a given); and that mining triggers increased deforestation within 1km of the mine site (a lot of which will be direct infrastructure for the mine and related to increased workers in the area - interesting but unsurprising). But giving quantitative figures (34 hectares forest loss for every 1 hectare of mining loss) to these is useful given how data deficient we are on mining impacts in sub-Saharan Africa. These figures are sufficiently useful for science, conservation and mining impact mitigation plans to justify publication from an interest standpoint.

The more interesting/surprising finding, that deforestation remains slightly elevated up to 20km from mines compared to background, is again useful. I have slightly more concerns about the degree to which this is real, given the background increase in deforestation rates in the key regions (ideally for this analysis deforestation rates country-wide would remain flat, so that before- and after-impact were against the same background). However, the authors have included sensible and comprehensive uncertainty estimation methods, and done various alternative analysis methods and sensitivity analyses - making me convinced the analysis is robust within its confidence ranges.

Thank you, we appreciate the constructive comments and detailed assessment of the remote sensing products and estimates used. We agree that extracting the “real” effects on deforestation is a key challenge for this type of work. Our quasi-experimental design leveraging group-time specific Difference-in-Difference (DiD) estimates of mined sites relative to sites that will become mines over our time frame (yet-to-be-treated design) allow robust estimation of this. Of course, the larger the buffer area analysed, the greater the number of potential secondary factors that could differ between sites, hence our decision to work up to 20 km but not beyond.

The findings are highly dependent on the accuracy of the deforestation detection in Hansen et al. (2013), and its attribution in the Malolele et al. (2024) paper. I know both datasets well, and have published previously on the errors and problematic use of the Hansen et al. (2013) dataset. I don't think these concerns apply here, and the datasets have been used

appropriately. Issues related to inclusion of plantations in Hansen et al. have been largely covered by use of an independent plantation dataset. And the Malolele et al. dataset finds mine sites well - they have a very distinctive spectral and spatial signature, which the machine learning and ~5m Planet data picks up well.

Some mines will be missed (particularly small or low impact mines), and some deforestation will be missed. The authors account for the missing mines in their Discussion, and there is no reason why forest loss data should be more likely to miss impacts before or after mining starts, so I do not think that will cause a bias.

There is a discontinuity in the Hansen et al. data, with some deforestation missed in the 2011-13 period where there was little Landsat data, and an increase in deforestation sensitivity after 2013 when Landsat 8 came on board. I have analysed the results graphs and have found no evidence of spikes around these dates - so I don't think this has caused a significant impact.

Overall, I am satisfied the conclusions of the paper are robust.

We are pleased that the Reviewer considers our use of both Hansen and Masolele data sets to be appropriate and that we have not incorporated substantial bias.

DETAILED COMMENT

1. Forest definition.

I find the definition of 'forest' as land with >50% forest cover very problematic. Legal definitions of forest are allowed within bounds set by the FAO and UNFCCC treaties (and IPCC reports) to vary between 10% and 30% canopy cover. Most of the relevant African countries, and recent relevant legislation/regulation (e.g. EU's EUDR) set the forest definition at 10% canopy cover.

I think the authors are really talking about something like 'dense forest', which is fine. This is what people often think of as 'forest' and picture deforestation as the loss of trees from an area that is near-closed-canopy forest. This is also the type of forest that is best captured by the Hansen dataset. It does poorly at detecting losses of low canopy cover forest, compared to high canopy cover - this is a major reason for the lower performance statistics in African countries, which have a lot of the world's low canopy cover forests.

I therefore would not suggest re-doing the analysis, but I would prefer the authors talking in the Abstract and relevant text points about 'dense forest' and 'deforestation of dense forest' or similar, rather than just defining forest in a way that does not match local or international definitions or understandings. They could also recognise more in the Discussion that for some of the countries discussed (particularly drier countries of southern and eastern Africa), they are only looking at a small subset of total forest in these countries. They are specifically looking here at mines near 'dense forest', and their impact on 'dense forest'. Mines in low canopy cover savanna that cause extensive losses of that low canopy cover forest are not looked at in this analysis. I think that's okay - but it should be pointed out.

The Reviewer raises a very important point. Whilst the 30% (or even 10% in legal cases as pointed out) forest definition is commonplace, we initially restricted our analysis to forest with >50% tree cover to prevent inclusion of tree cover loss in the widespread savannahs and very open woodland that exists across sub-Saharan Africa, since tree cover loss here represents a different issue to deforestation of dense forest (e.g. many savannas have been degraded by woody encroachment). Whilst the 10% number is policy relevant, we agree it would lead to inclusion of areas of savannah that many would not deem forest and introduce more error into our analysis since the Hansen dataset performs poorly at such low canopy cover.

We have now changed the paper to refer to dense forest throughout, and added some additional text into the Discussion to cover the point that in the drier countries of Southern and Eastern Africa, we are considering only a subset of the forest in these countries using this definition:

L236 - "In addition, we focus on dense forest (>50% tree cover) which excludes a subset of mining-driven deforestation in regions with drier, more open forest with lower tree cover (e.g. areas of southern and eastern Africa). We also do not examine any potential increases in degradation, particularly from small-scale or sub-canopy mining which are difficult to detect at 30m resolution (34, 35) and as such the study may underestimate the total extent and impact of small-scale mining."

Referee #2

1) it would be useful to clarify the distinction in this work versus the work by Sonter et al. (2017). That study looked at a different region (Brazil) and used a matching approach so differs methodologically. Although you cite that study, you do not make any direct comparisons or references to it, which is surprising, given that it attempts to do the same basic assessment (determine how much deforestation in nearby areas is attributable to mining). In fact in the discussion you note "we lack regional-scale understanding... in other key biodiversity and carbon hotspots... such as the Amazon Basin..." This conclusion does not seem warranted in full.

The Sonter et al. study is indeed a seminal work in the Brazilian Amazon and we should have made more of a comparison with their findings and our own, notwithstanding that the studies are distinct methodology (e.g. in classification of forest cover, analytical methods, target estimand and counterfactual). We have now added a new Discussion section broadly contextualising our study amongst previous impact assessments.

L254 - "Prior studies quantifying mining-driven deforestation at regional scale have predominantly focused on quantifying the losses driven by the direct mining footprint (*10*). Finer-scale studies focusing on mining impacts offsite have found additional deforestation up to 5 km from 255 artisanal mines in eastern Democratic Republic of Congo (*21*), up to 10 km from 446 mine clusters in the southern Côte d'Ivoire (*36*), and up to 70 km from 50 large industrial mines in the Brazilian Amazon (*20*). The substantially larger area of impact in the

Brazilian Amazon is likely a product of the larger mine size and the creation of significant new road and rail infrastructure linking large mines and ports (*20*)."

Also, in the Discussion sentence highlighted by the Reviewer, we had specifically referred to the lack of wall-to-wall mining occurrence and commodity data, limiting the coverage of current impact assessments. We have now made that clearer in the context of Sonter et al.

L293 – "However, at global scale, comprehensive wall-to-wall data on the spatial distribution of mines is still lacking, being largely limited to mines reported in online databases (*33*). We thus lack a regional-scale understanding of the additional mining deforestation footprint (both direct and offsite) across specific commodities in other key biodiversity and carbon hotspots that are particularly threatened by mining activity, such as much of South America and the tropical lowland forests of South-East Asia (*3*, *29*, *30*)."

2) The empirical strategy is reasonable in general, but also part of an active research frontier. The authors have used the basic approach in Callaway and Sant'Anna, with robustness assessment using the approach suggested by Gardner. They do not try the stacking approach suggested by Wing et al. The authors also explicitly do not conduct matching analysis, arguing that the un-mined sites would be systematically different. Although the authors include an array of robustness checks and whatnot, it would be useful to show the basic model estimates and parameters, especially for the covariates used in some of the models. I understand there are lots of models for individual countries and whatnot, so maybe only do this for the all-Africa regressions.

Thank you and we acknowledge the field is rapidly moving and what can be considered best practice at the present time can and likely will change as future research highlights new pitfalls and biases. We therefore tried to formulate a likely suite of reasonable and "*correct*" alternate modelling specifications to assess if the inference drawn from each approach aligns (see Supplementary Figures 1-8, 11-14, 18-23).

Nevertheless, the Reviewer correctly points out that there is an alternative stacking approach (Wing et al., 2024), so we have now reanalysed our work to include an updated variation of Wing et al.'s approach (as detailed here https://www.nber.org/system/files/working_papers/w32054/w32054.pdf). In brief, the updated approach modifies a standard stacked DID regression approach, which often fails to estimate a defined causal parameter due to improper weighting, to now apply corrective weights when stacking to estimate a trimmed ATT focusing around a specific trimmed period either side of the treatment event.

In sum, using the stacked method (Wing et al.) often produces highly similar (almost identical) estimates to the Callaway and Sant'Anna, and Gardner approaches, and on occasion suggested our results may be conservative. We have added reference to this in the main text and Methods:

Main text, L132 - "We repeated our main analyses using a recently proposed alternate heterogeneity robust two-stage imputation-based DiD estimator and a weighted stacked DiD

estimator (*31*). The results from these reanalyses support our findings, and in some cases suggest that they may be conservative (Supplementary Text 1, Supplementary Figures 2-8)."

Methods, L505 - "We also repeated our main national and sub-Saharan Africa-wide analysis using a second alternate stacked difference-in-differences trimmed aggregate ATT (*63*). In brief, this modifies a standard stacked DID regression approach, which often fails to estimate a defined causal parameter due to improper weighting, to apply corrective weights when stacking to estimate a trimmed ATT focusing around a specific trimmed period pre and post-treatment. For our analysis, we created trimmed sub-experimental periods with a 5-year pre-treatment and 5-year post treatment window. This method generates highly similar results to our main text approach, but when checking the parallel trends assumption of this approach we found it performed inconsistently for our data (Supplementary Figure 7 and 8)."

We have also added visualisation of these findings in Supplementary Figure 6, and a direct comparison of the SSA level results for all methods in Supplementary Figure 7. [Please note that we do not apply the Wing et al. approach to reproduce Figure 3 and 4 in the Supplement, because as shown by Supplementary Figure 6 at the national level the deviation from the parallel trends assumption is substantial]. Regarding what we present in the Figures, we have elected to present the targeted event-study estimates e.g. the estimated differences in years preceding and following treatment. We do not think it is appropriate to present control covariate parameter estimates for the covariate adjusted models as these cannot be interpreted in a causal manner, despite that being how readers may choose to interpret them. A large body of work has been done on the potential damage of interpreting control variables and the general recommendation is that these should not be emphasised and should be omitted from estimate tables and summary plots to prevent misinterpretation (Westreich & Greenland, 2013 Am J Epidemiol 177(4):292-298; Hünermund and Louw, 2023 Organizational Research Methods 28(1):138-151). Determining what mine features or landscape level covariates drive impact heterogeneity is an active field of research and would require an alternate analytic approach. We have added reference to the importance of this in future work:

L277 "Offsite deforestation risk is further compounded by the diversity of mechanistic pathways for impacts around mines (e.g. settled workers or agricultural expansion) that require improved understanding. Whilst we examine the expansion of land uses around mines, how differing mine features may contribute to impact heterogeneity remains an important research frontier."

Given that an existing result for Brazil exists that uses a matching approach to identify the effects rather than the before/after, it would be useful to also conduct the matching analysis. This is a lot of additional work, I understand, but the identification argument here is that you can identify the increase in deforestation by comparing to not yet treated plots, but if the concern with matching is that the outside locations are known as areas without mining resources, doesn't that mean there is systematic bias in the DID identification strategy used here?

We don't believe so. Our results remain consistent even after conditioning on covariates that would be used for matching. Likewise, our main estimator performs well in meeting the parallel trends assumption. Additionally, geological and economic suitability between treatment and

control units is shared by construction using this design, meaning there is less scope for mis-representation of the variables influencing mining impact. Where there is long term data, with treatments occurring across that time period, yet-to-be treated designs offer an elegant approach to impact assessment. These approaches specifically ask what additional impact mines have after establishment in a dynamic DiD or event study framework, this is in contrast to prior studies comparing how much deforestation there is in mined areas compared to unmined areas (Sonter et al., 2017). Both important, but distinct questions.

We have added reference emphasising the results from the Brazil study. However, we have to strongly encourage caution with such inter-study comparison as many things, including the matching/not matching distinction, the estimand, the time period, the scale of mining sites studied, the underlying data (e.g. the Hansen layer and how this was classified) and approaches to inference (e.g. a group-time ATT DiD approach vs a static non-temporal difference in deforested area) differ between the studies. Of course, as spatial data on mine locations becomes more available an overarching methodologically consistent global study will ultimately better facilitate such comparisons.

We have added text contrasting our results with the Brazilian example.

L254 - "Prior studies quantifying mining-driven deforestation at regional scale have predominantly focused on quantifying the losses driven by the direct mining footprint (*10*). Finer-scale studies focusing on mining impacts offsite have found additional deforestation up to 5 km from 255 artisanal mines in eastern Democratic Republic of Congo (*21*), up to 10 km from 446 mine clusters in the southern Côte d'Ivoire (*36*), and up to 70 km from 50 large industrial mines in the Brazilian Amazon (*20*). The substantially larger area of impact in the Brazilian Amazon is likely a product of the larger mine size and the creation of significant new road and rail infrastructure linking large mines and ports (*20*)."

3) The commodity specific estimates are a useful innovation. Given deficiencies in the data that you note, how confident are you in comparing across these results (they are, after all, just a reflection of the main results)?

They are not necessarily a reflection but a disaggregation. We are confident that the commodities mined are correct, as this is a separate and ground-truthed data set (see Maus, 2026). However, we encourage caution as commodity-specific impacts are very much a research frontier and a key point we seek to make with this analysis is the serious lack of robust data for the majority of mining sites. Thus, while we are able to confidently interrogate a sample of mines where the commodity extracted is known, we emphasise that there is a greater number of mines for which we simply do not know what is extracted.

L232 - "Likewise, the mining commodity data used is the most comprehensive available but has key omissions, particularly for poorly documented regions, unregulated or small-scale mining sites, and construction minerals (*33*). This restricts our analysis of commodity impacts to a subset of mines that have commodity data available; thus, we encourage caution when generalising these results."

L299 - “Our understanding of where specific minerals are extracted is limited, with our study only able to link fewer than 10% of mines to a commodity. This data paucity hampers the ability to distinguish between and address commodity-specific environmental impacts at scale, and highlights the need to work with the mining community to develop better understanding of mining footprints and environmental outcomes (*46*).”

4) There needs to be more discussion about the 187,000 hectare deforestation number in the abstract. First, the number does not seem all that large given the 20 year period in question and the area. But I guess this is just the land in <1 km?

The 187,000 hectare value is the direct deforestation driven by the mine footprint itself, so this only includes pits, tailing ponds, spoil heaps etc. This number includes all direct mining-driven deforestation (>50% tree cover) as per Masolele et al. across the entire region. Whilst the number may seem small over the time period and spatial extent, as we show later that the majority of mining footprint is from additional infrastructure (e.g. roads, settlements) and other offsite impacts, which can amount to over 30 times the deforestation and is not included in the 187,000 hectare figure. We have edited the Abstract and Discussion to make this clearer:

Abstract:

L27 “ finding 187,000 hectares of direct mining-driven deforestation (pits, tailing ponds, spoil heaps) across 16,627 mines between 2001 and 2020.”

Discussion:

L243 “This study provides comprehensive estimates of mining-induced deforestation of dense forest across sub-Saharan Africa identifying 187,070 ha of direct mining-induced deforestation—for pits, tailing ponds, spoil heaps, etc —between 2001 and 2020. This is almost four times the direct mining induced deforestation footprint recorded across Africa in prior studies limited to industrial mining (*10*). Critically, this direct deforestation is dwarfed by an 8pp increase in total deforestation triggered within 1 km compared to unmined areas, with elevated deforestation effects persisting up to 20 km from mines.”

Second, in Figure 3, it would be useful to present the aggregate deforestation number in tabular form for the concentric rings (<1, 1-5, etc.). COD looks enormous for instance, with perhaps >2,000,000 in rings 1-20km? In Figure 3 you should include the aggregate numbers, while in the supplement table 1, you can do the same thing for each country.

An aggregate area-based deforestation total is not the specific estimand targeted by our approach. While theoretically this value could be achieved through multiplying the modelled mean national ratios with the national mine footprints, we are cautious to do so. Such an approach, while present in the literature, would overlook uncertainty and shift any emphasis to this derived average estimate. While we undertook comprehensive spillover analyses that highlighted predominantly very low, but heterogenous spillover effects, a small degree may be present thus risking double counting when multiplying these values across landscapes. We would prefer not to emphasise a “back of the envelope” style calculation, that could be picked

up and misused by subsequent readers and/or media, in favour of focusing on the targeted modelled quantities.

Referee #3 (Remarks to the Author):

The topic is highly relevant and the findings are important. However, the authors make use of existing datasets to derive the patterns, without data modification or evaluation. This also means that the authors seem to fully trust the datasets from references 22 and 23, as they did not put much effort in evaluating their usability for their case. There are sensitivity analyses regarding the analytical methods, but that does not include any impact of uncertainty of the datasets used.

Thank you for the comments. The Masolele et al. data has very high accuracy statistics for mining classification (98% User's accuracy and 82% Producer's accuracy) so we are confident in its performance in mapping mining activity across Africa. We have manually verified a sample of mine polygons using ESRI WayBack to confirm mine presence and separation from surrounding landcovers (see more detailed response and illustrative Figures to a later comment).

Regarding the Hansen et al. data, we are using a relatively high tree cover (>50%) cut off which is double the recommended value at which tall woody vegetation can be unambiguously identified (Hansen et al., 2010). In support of our use of the Hansen and Masolele datasets, we also note that Reviewer 1 considers that we have done so appropriately. We are therefore confident that the deforestation presented in this paper is accurate, but should Reviewer 3 like additional clarification, we will be happy to do so.

For this level of work, this seems to be too simple, and I would suggest to add a more thorough evaluation of the applied datasets, in particular from reference 22, and also expand the analysis by adding more own contributions. That could be, for example, more detailed information on the mining related deforestation, such as road construction, etc.

We have now undertaken a separate supplementary reanalysis looking at the different potential drivers of deforestation around mining sites. To do this, we further leveraged the Masolele et al. data (the only data source to our knowledge with this data at this resolution and expanse). We used this to track post-deforestation land uses around mines for three key categories - agriculture, settlements and roads. We then applied the same robust DiD approach to track the additional mining driven change in agricultural land, settlements and roads. This revealed that the majority of post-mining deforestation can be attributed to agricultural expansion, with a substantial portion also stemming from settlement establishment and a tiny portion due to expanding road networks. See the revised main text, Methods and Supplementary Figures 9 and 10.

Main text, L160 - “The majority of offsite losses were due to the additional agricultural expansion triggered by mine establishment, with concurrent settlement expansion also an important driver, and road development representing a small portion (Supplementary Figures 8-9).”

Methods, L456- “ In a Supplementary analysis, we further disaggregated the non-mining deforestation data (indirect) to yield annual time series specifically for deforestation driven by agriculture, settlements, and roads (as derived in Masolele *et al.*) in the 0-5 km buffer. We then modelled this in the same dynamic DiD framework as described above to elucidate the additional impact mine establishment specifically had on agricultural and settlement expansion and road development (Supplementary Figures 9-10).”

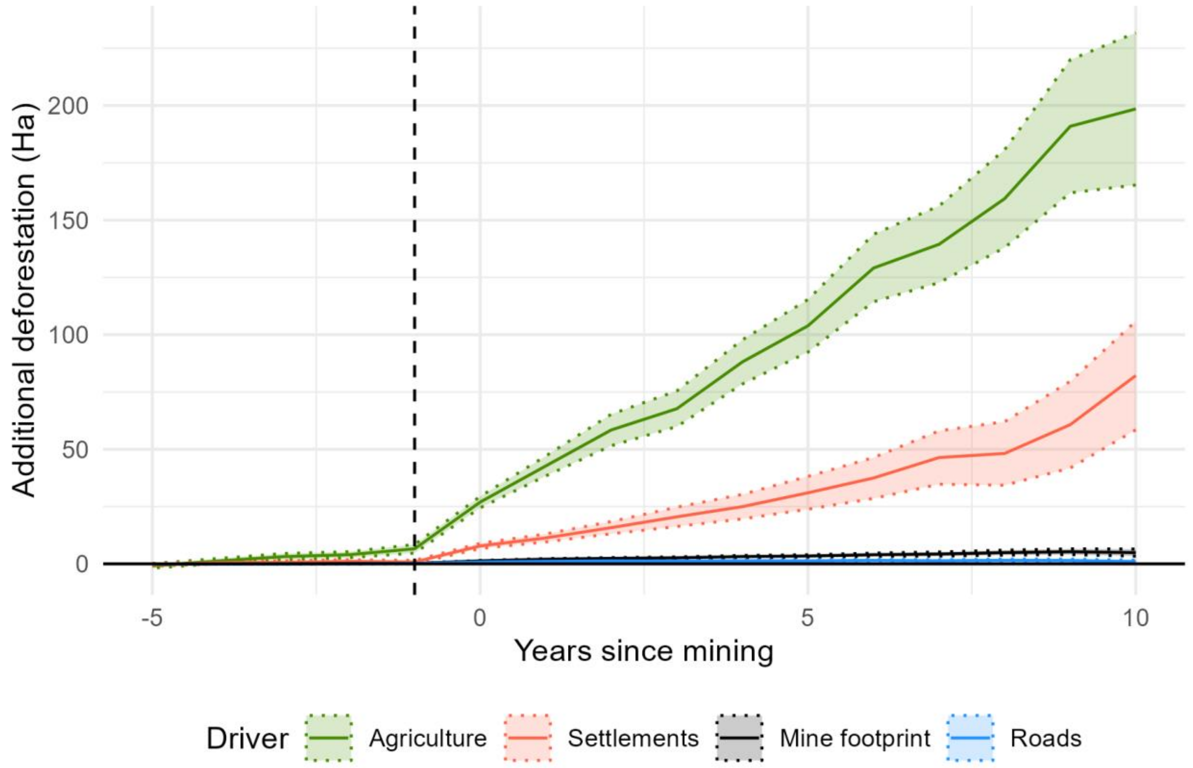

Supplementary Figure 9. Additional cumulative deforestation in the 0-5 km mining buffer for four post-deforestation land uses across sub-Saharan Africa. Lines at Points are mean ATTs and error ribbons are 95% confidence intervals.

-Forest loss, also in reference 22, comes from 30-m Landsat data. That implies that both forest loss and mining areas must have a large size, missing many small ones and also missing forest loss. Are there other products that could be used to evaluate the forest loss data? What about this dataset: https://forobs.jrc.ec.europa.eu/TMF? Maybe acquire high resolution images for a specific site and see how well the forest loss and the mining mapping are covered?

The original data of mining footprints from Masolele et al were derived using the high-resolution (5m) Planet imagery, which is able to distinguish the distinct spectral signature of mining

effectively. The maps were then aggregated up to 30 m by Masolele to conform with the Hansen *et al.* forest loss data, a process which coarsens the resolution and may lose very small and isolated sub-30 m mines. However, accurately detecting and mapping mines of such a small scale remains highly challenging and unattempted at scale. The JRC TMF dataset the Reviewer links is also a very useful tool, but does not represent a step-up in resolution since like Hansen it also uses Landsat imagery and is a 30-m resolution product. We used Hansen over JRC TMF, because the former has global coverage whereas the latter is restricted to the Tropical Moist Forest ecoregion and thus only available across a subset of the countries we analyse (but see extra analysis detailed below).

To further allay the Reviewer's concern, we have investigated some examples of mines using higher-resolution imagery (ESRI Wayback, where available) and find the tree cover loss and Masolele et al mine data to be effective, including distinction in the Masolele data between mining, roads and urban settlements. Please see these below:

[REDACTED]

[REDACTED]

[REDACTED]

As the Reviewer suggests, we have repeated our main SSA-wide analysis using the tropical moist forest (TMF) deforestation data incorporating all mines that occur within the TMF. We contrast the results of the analysis using the Hansen et al. data with results using the TMF data (for those mines located within the TMF biome). This comparison focuses solely on the African tropical moist forest biome so has a much-reduced sample size (n = 7859) relative to the main analysis that incorporates all dense (>50%) forest cover across SSA (n = 16,627).

We find a very high level of agreement between the Hansen and TMF forest loss data. The TMF results are slightly more uncertain at larger distances, but the overall inference drawn and mean additional forest losses remain consistent. The greater uncertainty is likely a product of the relatively smaller extent of the high canopy cover (>90%) forest typically detected in the TMF. We are thus confident in the main text results which extend across biomes in Sub-Saharan Africa.

See edits to Methods;

L543 "(6) To assess whether our results are sensitive to the specific forest loss data used we repeated our analysis using the European Commission Joint Research Centre Tropical Moist Forest (TMF) data (*65*). We processed this identically to the main analysis generating mine cluster specific deforestation time series analogous to those used in the main analysis. As the TMF data only covers a specific biome, unlike the Hansen et al., data this led to the analysis focussing on a subset of mines within areas dominated by tropical moist forest (*n* = 7859). As a comparison we also repeated the Hansen et al., based analysis for this subset of mines to

check for congruency or systematic differences due to the choice of forest loss data (Supplementary Figure 23)."

Supplementary text;

"*(6) Alternate forest loss data.*

Repeating our analysis using the JRC TMF forest loss data yielded highly similar results to using the Hansen et al., forest loss data when restricting this to mines from the tropical moist forest biome. We note very high congruency between mean estimates of forest loss across all buffers, and therefore consistent inference regardless of the forest loss data used (Supplementary Figure 23). However, there is greater uncertainty when using the JRC TMF data set particularly in the 10-20 km buffer zone. We suggest this is likely due to the JRC TMF data only capturing dense forest, typically 90% canopy cover, so the starting area is likely to be smaller and any subsequent losses more uncertain."

And Supplementary Figure 23.

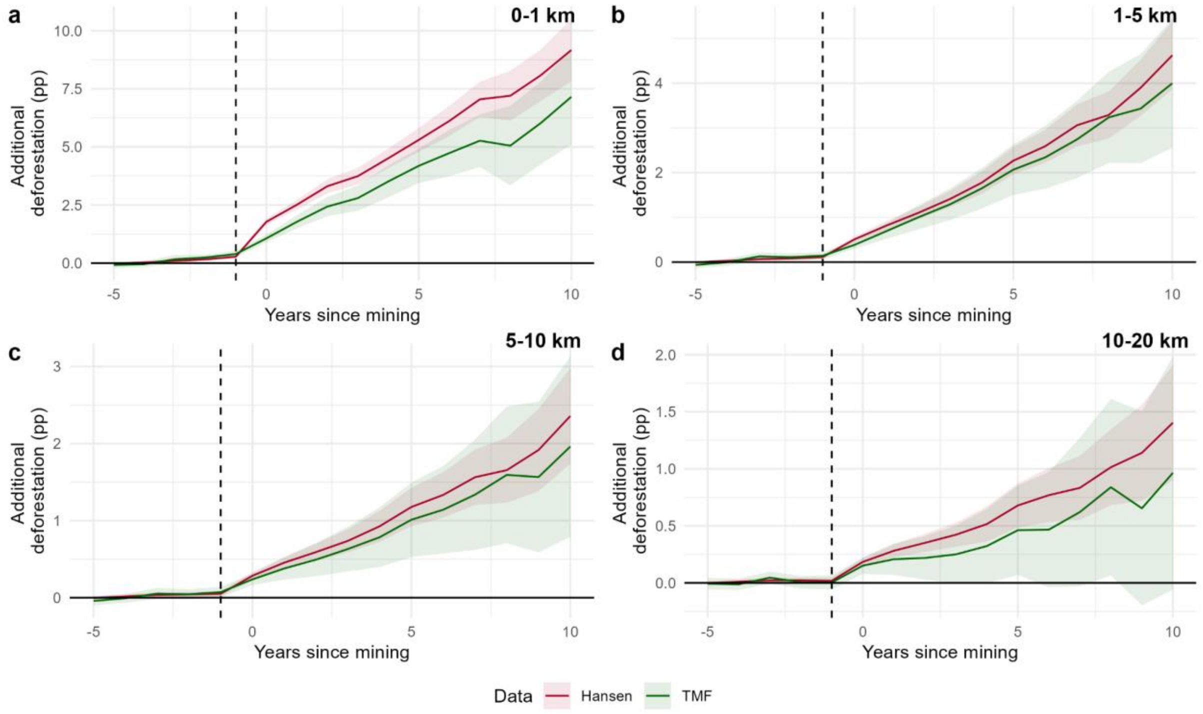

Supplementary Figure 23. Sensitivity analysis comparing the Hansen et al., and JRC TMF forest loss data, restricted to the tropical moist forest biome. Solid lines denote the mean additional deforestation and the dashed ribbon the 95% confidence interval.

-Could the mining areas be evaluated? How does the database from Maus et al (2022) match with the mining areas of reference 22? Are there any other studies that have used higher resolution sensors to map mining areas in Africa that could be used for comparison? I noticed Supplementary Fig 9 but it is not a real evaluation of the mining area mapping. I understand that using different datasets, the conclusion remains the same, however, the conclusion that mining triggers deforestation is not entirely surprising, so the numbers are in fact important.

Our analysis uses the most up-to-date and high-resolution data on the spatial distribution of mines. We are aware of two other assessments, conducted at far smaller spatial scale, of mine locations in Africa: (1) a West African deep-learning-based assessment of mine occurrence (Couttenier et al., 2022, *PLoS One*); and (2) a manual assessment of 469 mining polygons (Ahmed et al., 2021, *Land Degradation and Development*). The methods used in Couttenier et al. yield far lower performance metrics than Masolele et al. (Couttenier recall = 42% vs Masolele et al = ~82%; Couttenier precision= 70% vs Masolele et al = ~97%), thus Masolele achieves a similar goal, more accurately and at the scale of sub-Saharan Africa. The Ahmed et al. approach found <3% of the total number of mines located by Masolele et al. in sub-Saharan Africa.

We also compared the degree of overlap between the mining clusters we produced using Masolele et al., with the mine polygons produced by Maus, which show good agreement and coverage. Specifically, 80% of the total area of mine polygons (per Maus 2026) overlap with a mining cluster identified in this study using Masolele et al. data. The 20% discrepancy is likely caused by the temporal mismatch between the two data sets. Maus integrates various data sources with irregular temporal coverage that will include mines detected beyond the time frame of the study (2000-2020). We are thus confident that we are using the most recent and accurate wall-to-wall remote sensing assessment of mine location available across Africa (Masolele et al.) and that this includes the vast majority of mines created in our study window.

-I understand that for the conclusion of this work, it is less important to map mining areas wall-to-wall, but it would be important to avoid miss-classifications. However, the question should be asked: What does it imply that the mapping resolution is rather coarse and many small mining areas are missed? What about forest degradation? I think it is at least a crucial discussion point that small mining sites are not part of this work, and that only large clear-cuts are mapped as forest loss, which overlooks smaller areas and single tree removal. The numbers on deforestation are thus likely much higher than reported here.

Whilst it is true the resolution of our dataset means that only mines that impact tree cover across a 30m pixel will be detected, this is unfortunately a caveat that we cannot change given this is the best available and highest-resolution deforestation time series dataset at our disposal (and one that has been used 1000’s times in studies quantifying deforestation). The Reviewer raises an important point about degradation, which can also be caused by mining, but this is not something we can strictly test since the only dataset with degradation data available is restricted only to the Tropical Moist Forest ecoregion, and thus would cover less than half our mining data. As for single tree removal, we are not aware of any large-scale products with sufficient temporal resolution capable of detecting such small-scale impacts across our study area. We agree with the Reviewer that this is an important discussion point and caveat, and have now included some additional discussion:

L238 “ We also do not examine any potential increases in degradation, particularly from small-scale or sub-canopy mining which are difficult to detect at 30m resolution (*34*, *35*) and as such the study may underestimate the total extent and impact of small-scale mining.”

L303 - "This expands beyond the impact of mining on deforestation as shown here, to also cover additional environmental effects, including groundwater and air pollution (*47*), human health impacts (*48*), and degradation of both forest and non-forest natural ecosystems (*49*). Quantifying these mining impacts is a key future research direction."

-The mining start year is defined based on reaching 10% mining pixel coverage, but this threshold seems arbitrary? So it could be useful to test the sensitivity of this value.

Apologies if it was not clear, we currently test three different versions of this threshold. These are when the first 30-m pixel is lost to mining, when 10% of pixels are lost to mining, and when 20% of pixels are lost to mining.

We describe this in the Methods:

L514 - "(3) In the main text analysis we categorise the commencement of mining operations as when 10% of a mining cluster is deforested due to mining. This minimises the risk that any single erroneously classified pixel substantially biases our analysis. However, we also reran our main analysis (Figure 2) using two alternate criteria, the first was less conservative and assumes mining commences the year the very first pixel is lost from the cluster, and the second is more conservative and requires 20% of the cluster must be directly deforested by mining before mining operations are assumed to have commenced (Supplementary Figures 12 and 13)."

We present these results in Figure 2, and Supplementary Figures 11 and 12, and we then briefly discuss this in the Supplementary Text:

"*(3) Alternate triggers for mining start year.*

Altering how we classify the commencement of mining had a minimal effect on our main results shifting our sub-Saharan Africa wide by a fraction of percentage point for any given time since mining with all estimated 95% confidence intervals remaining highly overlapping (Supplementary Figures 12 and 13). Broadly, comparing the single pixel, 10% and 20% deforested triggers for the commencement of mining, we note no clear directional shift in estimates becoming greater or lesser as the stringency of the trigger increases. We attribute this to the general high congruency between the years identified by the three triggers (years identified by the three triggers all have pairwise Pearson's correlation statistics >0.7), with many clusters having identical start years identified regardless of methods (e.g. mining begins and expands substantially in the same year)."

- It could be good to have more detailed information on what is going on around the mining sites. What is causing these forest losses? Can you quantify the reasons for forest loss? What kind of trees are lost (maybe use canopy height as a measure to get an idea if it is old forest)? Adding information may help to reader to trust that there is in fact causality between the deforestation in the buffer and the mining activities. You could also generate a new mining

dataset using NICFI images from 2018–2025, maybe just for a small area to confirm that the access roads were built for mining activities.

We have now added a separate supplementary reanalysis looking at the different potential drivers of deforestation around mining sites. To do this, we further leveraged the Masolele et al. data (the only data source to our knowledge with this information at this resolution and expanse). We used this to track post-deforestation land uses around mines for three key categories - agriculture, settlements and roads. We then applied the same robust DiD approach to track the additional mining-driven change in agricultural land, settlements and roads. This specifically asks what is the additional deforestation triggered by mine establishment that subsequently became roads/agriculture/settlement, and thus directly addresses the Reviewer's point by assessing if mine establishment does trigger greater than would be expected road building. This revealed that the majority of post-mining deforestation with 5 km of a mine can be attributed to agricultural expansion, with a sizable portion also stemming from settlement establishment and a small portion due to expanding road networks. See the revised main text, methods and Supplementary Figures 8 and 9, which we included in response to an earlier point by the Reviewer. We feel that generating a new mining data set using the U-net active learning model and the NICFI data would represent an entirely new study in need of substantial methodological interrogation and peer-review.

Regarding the suggestion to explore changes in canopy height, we look over a wide range of forest ecosystems and forest types, and it would be difficult to use canopy height to determine forest age or type given the broad structural attributes of the systems assessed. Alternative data sources for old/undisturbed forest exist, but unfortunately are restricted to subsets of our study area (e.g. humid tropical forest, Vancutsem et al., 2022. Turubanova et al., 2018).

-Nevertheless, the question about causality should be asked. Although a pseudo-start method is used, how can you be sure the deforestation is in fact an indirect result of the mining activities?

We use a quasi-experimental approach specifically designed to elucidate causality by comparing deforestation pre-and post-mine establishment in treated and not-yet treated plots. We are confident this is a robust and appropriate manner to detect causality.

However, the Reviewer is correct, knowing when a mine specifically began operating is not data that currently exists. Thus, we had to use a pseudo start method (see also Ladewig et al., 2024 *Nature Sustainability* 7:1452–1460). To probe the sensitivity of this pseudo start approach, we tested three alternate cut-offs to define mine start years (first pixel, 10% and 20%, see response above). Given the high congruence between the results produced we are confident our inference is robust to uncertainty in the exact timing of mining commencement. See previous response for the in-text changes (namely the Methods, Supplementary Text and Supplementary Figures 12 and 13).

Referee #4 (Remarks to the Author):

This paper assesses the amount of deforestation associated with mining in Sub-Saharan Africa, differentiating between direct mine-site impacts, and deforestation at different distances from the mine site. The results are presented across countries and where possible across mineral types. The results add to a growing body of literature empirically demonstrating and measuring the impacts of natural resource extraction on forest cover. They are a useful contribution for their focus on the continent.

Thank you and we appreciate your constructive feedback.

I do not have any particular quibbles with the findings, but I am unsure what to do with them. While I appreciate that it is not the role of science to give actionable findings, in this instance I think that there is perhaps some value in speaking more cogently to policy both because the focus of the research is chosen deliberately for its relevance to and currency within contemporary policy debates, including some of those recently at the COP-30 in Belem, Brazil. Also, the discussion is framed largely in terms of policy relevance. That discussion speaks mostly to questions of improving Environmental Impact Assessments as a means of addressing deforestation risk. However, the analysis does not tell us what share of the mining considered might even be susceptible to the conduct of EIAs. The discussion refers to "artisanal" and "industrial" mining as two forms of mining in the continent. However, the analysis does not seem to distinguish between the two types of mining. Thus we are unable to know if and how patterns of deforestation on and off-site vary depending on whether the mine is an industrial mine (presumably meaning large scale machinery) or artisanal (presumably referring to mining with rustic tools. One assumes that the off-site effects of these types of mining would differ considerably too because they are associated with quite different forms of population movement, food supply needs, and associated infrastructure. Similarly, there is no distinction between whether the mines are formally registered and owned, or are non-legal mines operated without title and beyond the bounds of government regulation. Only some of these mines would even consider conducting an EIA. And even in those cases that might be candidates for EIAs, whether they do one or not depends on the quality of their governance and oversight by the state. Presumably for the patterns of deforestation identified by the authors to exist, the mines must already be failing in their EIAs and the governments failing in requiring them or holding mines to account on the basis of these prior assessments. This makes a discussion calling for better EIA's a little bit moot.

I think that not disaggregating patterns by type of mining is also problematic because the paper refers to the impact of "mining" on deforestation when in practice it may be the impact of a particular type of mining. Which type of mining would also have implications for the discussion section, because policy and regulatory responses would presumably vary a lot for different types of mining.

As the Reviewer states, we are unfortunately unable to distinguish between artisanal and industrial sites with confidence. To our knowledge, this data does not exist and we believe this

is not something that would be easily detectable via remote sensing since the distinction relates predominantly to the lack of formal employment status and reliance on manual labour rather than a particular spectral signal detectable with satellites. We hypothesize two potential ways to separate out the two mining types (although, we see considerable flaws with both approaches):

(1) The Maus data includes non-artisanal mining sites and thus could theoretically be used to isolate semi-industrial and industrial mining. However, we are aware the Maus data is not wall-to-wall and misses a large number of semi-industrial and industrial mines, meaning a Maus-only analysis would represent only a subset of the mines included in our analysis. We currently show the Maus-only analysis in Supplementary Figure 13 as a 'ground-truthed' comparison to our remote sensed data.

(2) Use some form of size-based distinction and state any mines smaller than a given size are artisanal. However, artisanal mines are not always particularly small sized (e.g. Sicomines/Kolwezi concession, and the Mutanda mine in the DRC, which can be 10's km^2) and we are not aware of any size cut-off having previously been explored or used to define artisanal mines (as the definition relates to the informal nature of the mining and methods employed, rather than size). If the Reviewer can point towards or suggest specific approaches or cut-offs, then we would be happy to explore them.

Nevertheless, we have undertaken an additional size stratified analysis looking how the additional impacts of mining change with mine size, by splitting mines into four quantiles. This demonstrates that across size classes mines drive an increase in deforestation, with that effect positively correlated with mine size. See Supplementary Figure 1.

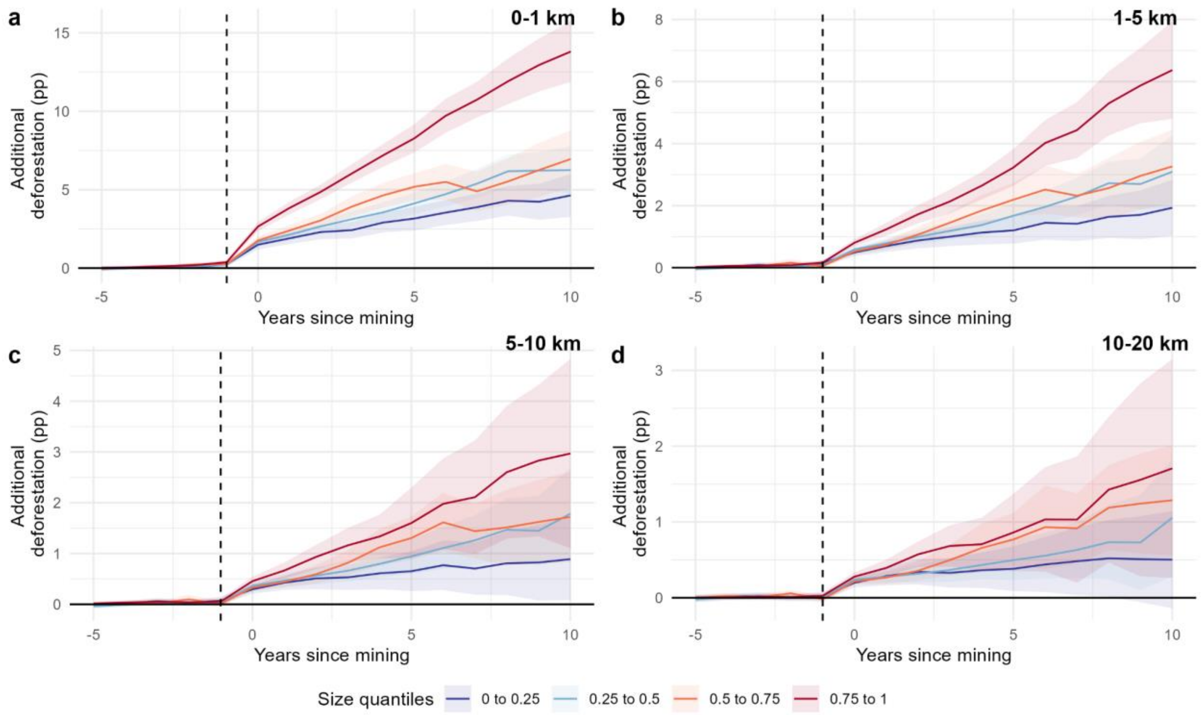

Supplementary Figure 1. Estimated additional deforestation since mining is detected across mining size classes in Sub-Saharan Africa. Additional percentage points (pp) of deforestation in the 0-1km (a), 1-5km (b), 5-10km (c) and 10-20km (d) buffers. Colours denote mine size quantiles ranging from the bottom 25% (0 to 0.25) up to the largest quarter (0.75-1). Lines are mean ATTs and error bars are 95% confidence intervals.

We have also added a new paragraph to the Discussion, detailing some of the difficulties associated with identifying different forms of mining and their associated impacts, as well as the difficulties in implementing EIAs when, as the Reviewer states, many mines are unregistered and therefore not formally registered and therefore not engaging with EIAs:

L277 - "Offsite deforestation risk is further compounded by the diversity of mechanistic pathways for impacts around mines (e.g. settled workers or agricultural expansion) that require improved understanding. Whilst we examine the expansion of land uses around mines, how differing mine features may contribute to impact heterogeneity remains an important research frontier. One important distinction is between industrial and artisanal mining, since these two forms will drive differing amounts of additional deforestation through varying population movement, food requirements and associated infrastructure (*21*, *42*). We demonstrate larger mines to have a greater deforestation footprint, but mine size is ineffective at distinguishing between industrial and artisanal (e.g. very large-scale artisanal mining in Kolwezi and Mutanda in the Democratic Republic of the Congo), and further work is required to map the different forms of mining and evaluate their individual off-site deforestation impacts. Mitigating offsite impacts may also be especially hampered in areas with weak governance, lax enforcement, or corruption (*43*, *44*). In addition, some of the mines included in this study and many operating across Africa more broadly (*45*) are unregistered, informal or illegal and therefore not subject to EIAs."

Editorial Comments

Please can you re-work the first paragraph to provide more context and more information on the methods. Please can you also take out any brackets and end this paragraph with implications that are more clearly tied to the conclusions in the study. Finally, please can you add uncertainty estimates where applicable.

We have now provided an additional sentence of context, removed brackets around text, added uncertainty estimates, and edited our recommendations.

Some specific comments on formatting

1. Please reduce the length of the title to 75 characters (with spaces) or less, so that it fits on two lines in the final layout.

Done.

2. Please add references to the abstract (if applicable).

Done.

3. Please create a separate reference list for any Methods references, making sure that the numbering continues from the main text references.

Done.

4. Please remove the main figures from the article file and re-supply them individually in an acceptable format such as EPS, AI, PS, PDF, PPT, PSD or XLS (for graphs) with editable vector files.

5. Please describe each figure panel in the figure legend.

This was missing in Figure 2 and has now been corrected.

6. Please reduce subheadings to 40 characters (with spaces) or less.

Done.

7. Please provide a Supplementary Information guide as a separate word document.

Done.

8. There are potential third party rights issues in the figures. Please check the sources of all illustrations and clarify whether permissions are needed to adapt or reproduce them. Please make sure to include the relevant details in third party rights table when you resubmit (more information below). If Biorender or a similar software has been used, please also ensure to provide relevant licenses. In particular please check figure/s: Figure 1a, 3a, 4a-g, 5a-c, Supplementary Figure 4a, 5a-g, 16a-g, 17a-b, 18a-e.

We have completed the attached form.

9. Please make sure to provide a third party rights table (more information below) when you resubmit. If Biorender or similar software has been used, please also ensure to provide relevant licenses.

No software requiring licences were used.
10. There are display items in the SI. Should any of these materials be moved to Extended Data?

We have 23 Supplementary Figures, we would strongly prefer to keep them in their current order in a single location in the Supplementary Information, combined with the relevant Supplementary Text before them. These are all re-analyses and robustness checks of our main analyses, to select 10 of them to present as ED would be arbitrary when all are valid alternate specifications of our modelling approach.

11. Please ensure that the text size in all figures is at least 5 pt Arial.

We believe all text is now size 6 or above.

Referee comments

Referee #1
I have read the response to reviewers and new draft in full, again with a focus on the remote sensing side of the paper.
I appreciate the paper now refers to 'dense forest' throughout, which is appropriate to the way they are using satellite datasets.
I am satisfied the interesting results they see are real, and not an artefact of the analysis. I have no hesitation in recommending this for publication.

Referee #2
The authors have addressed my key concerns adequately including new analysis using parallel methods.

Referee #3
The authors have answered my comments and those from the other reviewers. I do not have further comments on the manuscript. The method seems to be sound.

Referee #4
1. I really enjoyed, and appreciated, the ways in which the authors engaged the four reviewers' comments. I imagine that some of the reviewers may still not be fully satisfied because of disagreements about preferred methodologies. But this is a very serious response, that included several new runs of the data.
2. I am quite satisfied with the response to my comments, and appreciate the text added in the Discussion.

We would like to sincerely thank all four reviewers for the helpful and detailed comments that have substantially approved the clarity and robustness of the final manuscript.
